# Engineering well-expressed, V2-immunofocusing HIV-1 envelope glycoprotein membrane trimers for use in heterologous prime-boost vaccine regimens

**DOI:** 10.1371/journal.ppat.1009807

**Published:** 2021-10-22

**Authors:** Emma T. Crooks, Francisco Almanza, Alessio D’Addabbo, Erika Duggan, Jinsong Zhang, Kshitij Wagh, Huihui Mou, Joel D. Allen, Alyssa Thomas, Keiko Osawa, Bette T. Korber, Yaroslav Tsybovsky, Evan Cale, John Nolan, Max Crispin, Laurent K. Verkoczy, James M. Binley

**Affiliations:** 1 San Diego Biomedical Research Institute, San Diego, California, United States of America; 2 School of Biological Sciences, University of Southampton, Southampton, United Kingdom; 3 Scintillon Institute, San Diego, California, United States of America; 4 Cellarcus BioSciences, La Jolla, California, United States of America; 5 Theoretical Biology & Biophysics, Los Alamos National Laboratory, Los Alamos, New Mexico, United States of America; 6 Department of Immunology and Microbial Science, The Scripps Research Institute, Jupiter, Florida, United States of America; 7 Frederick National Laboratory for Cancer Research, Leidos Biomedical Research, Inc., Frederick, Maryland, United States of America; 8 Vaccine Research Center, National Institute of Allergy and Infectious Diseases, National Institutes of Health, Bethesda, Maryland, United States of America; University of North Carolina at Chapel Hill, UNITED STATES

## Abstract

HIV-1 vaccine immunofocusing strategies may be able to induce broadly-reactive neutralizing antibodies (NAbs). Here, we engineered a panel of diverse, membrane-resident native HIV-1 trimers vulnerable to two broad targets—the V2 apex and fusion peptide (FP). Selection criteria included i) high expression and ii) infectious function, so that trimer neutralization sensitivity can be profiled in pseudovirus (PV) assays. Initially, we boosted the expression of 17 candidate trimers by truncating gp41 and introducing a gp120-gp41 SOS disulfide to prevent gp120 shedding. "Repairs" were made to fill glycan holes and eliminate other strain-specific aberrations. A new neutralization assay allowed PV infection when our standard assay was insufficient. Trimers with exposed V3 loops, a target of non-NAbs, were discarded. To try to increase V2-sensitivity, we removed clashing glycans and modified the C-strand. Notably, a D167N mutation improved V2-sensitivity in several cases. Glycopeptide analysis of JR-FL trimers revealed near complete sequon occupation and that filling the N197 glycan hole was well-tolerated. In contrast, sequon optimization and inserting/removing glycans at other positions frequently had global "ripple" effects on glycan maturation and sequon occupation throughout the gp120 outer domain and gp41. V2 MAb CH01 selectively bound to trimers with small high mannose glycans near the base of the V1 loop, thereby avoiding clashes. Knocking in a rare N49 glycan was found to perturb gp41 glycans, increasing FP NAb sensitivity—and sometimes improving expression. Finally, a biophysical analysis of VLPs revealed that i) ~25% of particles bear Env spikes, ii) spontaneous particle budding is high and only increases 4-fold upon Gag transfection, and iii) Env+ particles express ~30–40 spikes. Taken together, we identified 7 diverse trimers with a range of sensitivities to two targets to allow rigorous testing of immunofocusing vaccine concepts.

## Author summary

Despite almost 40 years of innovation, a vaccine to induce antibodies that block HIV infection remains elusive. Challenges include the unparalleled sequence diversity of HIV’s surface spikes and its dense sugar coat that limits antibody access. A growing number of monoclonal antibodies from HIV infected donors provide vaccine blueprints, but have been difficult to induce by vaccination, due to their unusual features. However, two targets, one at the viral spike apex and another at the side of the spikes are more forgiving in their ’demands’ for unusual antibodies. Here, we made a diverse panel of HIV spikes vulnerable at these two sites to be used as vaccines to try to focus antibodies on these targets. Our selection criteria for these spikes were: i) that when expressed on particles, they are infectious, allowing us to evaluate immunogens and vaccine sera using particles made with the *same* trimers, ii) that spikes are easy to produce by cells in quantities sufficient for vaccine use. Ultimately, we selected 7 trimers that will allow us to explore concepts that could bring us closer to an HIV vaccine.

## Introduction

Contemporary HIV-1 vaccine candidates can routinely induce high titers of autologous tier 2 neutralizing antibodies (NAbs) [1–5]. However, cross-neutralization is sporadic [6–8]. This may be because vaccine NAbs tend to target strain-specific gaps in the envelope glycoprotein’s (Env’s) carbohydrate shell, i.e., “glycan holes” and the glycan-free base of soluble trimers [1,5,9–11]. By contrast, broad NAb (bNAb) targets are usually protected by glycans that nascent NAbs must evolve to avoid and/or engage [12–14].

Scores of bNAbs have been isolated from HIV-1-infected donors over the past decade, targeting 5 major conserved epitope clusters: the V2 apex, V3-glycan, CD4 binding site (CD4bs), gp120-gp41 interface/fusion peptide (FP) and the gp41 MPER [15]. Exciting studies now show that NAbs with some breadth can be induced in some vaccinated animals [7,16,17]. Further efforts are needed to improve vaccine-induced NAb breadth, titer and consistency.

Strategies to improve vaccine NAbs may be divided into 3 non-exclusive tracks [18–21]. One is to trigger the expansion of bNAb precursor B cells (unmutated common ancestors: UCAs) [22–27]. Vaccine-induced UCA triggering [28–34] may be improved by removing clashing glycans [3,7,26,35,36], reducing glycan size [14,37–40], or by priming with core epitopes or scaffolds [8,16,17,23,34,41–45]. Ideally, priming creates an initial diverse pool of antibodies (Abs) that can be guided to breadth by carefully selected boosts [8,35,46]. A second approach is to recapitulate natural bNAb development, using patient-derived Env clonal lineages [13,25,33,47–54]. In this case, UCAs may be triggered by Envs from the source donors [38,39]. A third strategy is to “immunofocus” NAbs, using trimers with high sensitivities to desired site(s) [3,7,8,35,36,41,55].

Repeated immunization with the same Env trimer may cause NAbs to overly focus on distinct features of the vaccine strain, resulting in autologous NAbs. The use of multiple trimer variants may help resolve this problem in any of 3 approaches [2,3,11,26,35,36,55,56]. 1) Repeated polyvalent mixtures in each shot [55,57]. Success may be limited by the lack of evolutionary direction for NAb development; 2) Use a highly sensitive prime, followed by modified matched-strain trimer boosts. This has been attempted with some success, typically by toggling glycans surrounding the epitope in primes and boosts [3,7,33,35,36,57,58]; 3) Serial heterologous prime-boosts (SHPB), uses *different strains* in each shot [2,55,56,59–61], thus eliminating the possibility of strain-specific NAbs. Success may hinge on whether nascent NAbs sufficiently cross-react with boosting trimers to keep them "on track".

Here, we sought to assemble a panel of diverse trimers expressed on virus-like particles (VLPs) to simultaneously immunofocus NAbs on the V2 apex and fusion peptide (FP) epitopes. The resulting VLP SHPB regimens will later be tested in vaccine models, e.g., the V2-specific CH01 "heavy chain only" (V_H_DJ_H_^+/+^) UCA knock in mouse [59]. These two epitopes were chosen for several reasons. First, since they do not overlap, the chance of inducing bNAbs is doubled. Second, both sites accommodate multiple NAb binding modes [8,43,55], increasing the number of compatible germline Ab precursors. FP NAbs have common-in-repertoire features, and can be induced in many species, including mice [8,16,43]. Some V2 NAbs (e.g., CH01, VRC38.01) also exhibit sufficiently common features, and thus do not depend heavily on rare V(D)J recombination and/or somatic hypermutation, thus also making them plausible vaccine prototypes [24,27,46].

V2 and FP NAbs both recognize protein/glycan composite epitopes. V2 bNAbs bind the N160 glycan and the neighboring basic C strand of a 5-strand β-barrel at the trimer apex [24,62,63]. However, the binding may be regulated by protecting V1V2 glycans and long V1V2 loops [13,26,40,55,57,64,65]. FP bNAbs, like VRC34 recognize the N-terminal gp41 fusion peptide, and the proximal N88 glycan of gp120, but clash with gp41’s N611 glycan [8,43,44,66,67].

The preferential binding of CH01 and VRC34 to trimers produced in GnT1- cells, in which glycan maturation is blocked, suggests that both NAbs contact the stems of their glycans (N160 and N88, respectively), and that bulky glycan head groups hinder their binding. CH01 often mediates sub-saturating neutralization, even at high concentrations [14]. This may stem from differential trimer glycosylation. Indeed, some sequons (glycosylation motifs) may be occupied by a variety of glycans or may be skipped altogether [68]. This variability could have direct consequences on NAbs that either bind to trimers or are unable to bind due to glycan clashes. Glycan variation may also impact V1V2 folding [69]. Since FP neutralization depends largely on the FP sequence, engineering trimers to maximize the induction of FP NAb breadth should be relatively straightforward. V2 bNAb ontogeny is more complex. In natural infection, a basic V2 C-strand (residues 166–171) promotes initial electrostatic NAb contacts. The C strand then becomes increasingly charge-neutral as the virus escapes, promoting NAb reactivity with anchor residues, usually N160 glycan, and conserved residues at positions 168, 169, 171 and 173 of the C-strand [24,25,37,57,70].

We previously showed that VLPs expressing native JR-FL trimers, like their soluble, “near native” counterparts (e.g., SOSIP), regularly induce potent autologous NAbs [1,11,36]. Advantages of SOSIP include facile manufacture and rational, structure-driven vaccine improvements [71,72]. However, one drawback is that the V2 apex is less compact [73,74], which may explain why they induce V2-specific Abs that differ markedly from V2 bNAbs [55,71,75]. Second, the glycan-free base of SOSIP is an immunodominant, non-neutralizing target that might dampen responses to desired sites [10]. Third, SOSIP partially exposes the V3 loop, thus inducing more V3 non-NAbs than VLPs [1,6,36,76–78]. Fourth, SOSIP exhibits more unoccupied sequons and a more heterogeneous and immature glycoprofile compared to native trimers [9,55,68,79–82], perhaps due to structural differences and/or to soluble trimer overexpression outpacing glycosylation machinery. Consequently, some SOSIP-induced Abs are unable to navigate membrane trimer glycans, even on the cognate strain [9,83,84].

The transmembrane domain and cytoplasmic tail of membrane trimers anchor, stabilize, and modulate the external spike conformation, in particular the V2 apex [85,86] in ways that cannot be readily achieved by soluble trimers. A further advantage of membrane trimers is that they can be directly checked for sensitivity in pseudovirus (PV) neutralization assays—thus, the same trimers are used in immunization and our desired readout. Accordingly, we primarily used NAbs CH01 (V2) and VRC34 (FP) as NAb probes to appraise candidate trimers, with more emphasis on V2, due to its complexity.

Adapting vaccine platforms from their initial prototypes e.g., JR-FL for VLPs and BG505 for SOSIP to other strains is not straightforward. Many strains do not form well-folded soluble trimers. Mutations can solve this problem, sometimes using BG505 Env sequences as “scaffolding” [87–91]. In contrast, since membrane trimers are by definition native, proper folding is typically not a problem. However, expression levels of native trimers of many strains is poor [92], although evidence suggests that expression can be improved [93–96]. Accordingly, we took two approaches to down select a panel of immunofocusing trimers. First, we screened for high expressing V2-sensitive strains. Second, we tried to increase the V2 sensitivity of well-expressed strains. From there, we used a variety of repairs and adjustments to select a panel of 7 diverse, well-expressed VLPs for prospective vaccine studies.

## Results

Here, we sought to identify a panel of trimers to immunofocus V2 and FP epitopes. Specifically, we sought well-expressed trimers with a range of CH01 sensitivities from at least 5 diverse strains to enable us to test various prime-boost vaccine concepts. Strains with broad sensitivity to multiple V2 NAbs are preferred. Ideally, the panel should encompass a range of NAb sensitivities, ranging from acute, UCA-triggering to that of typical transmitted isolates. All selected trimers should be functional in pseudovirus (PV) assays, so their NAb sensitivity can be calibrated and should not be overtly sensitive to V3 MAbs.

**Fig 1:**
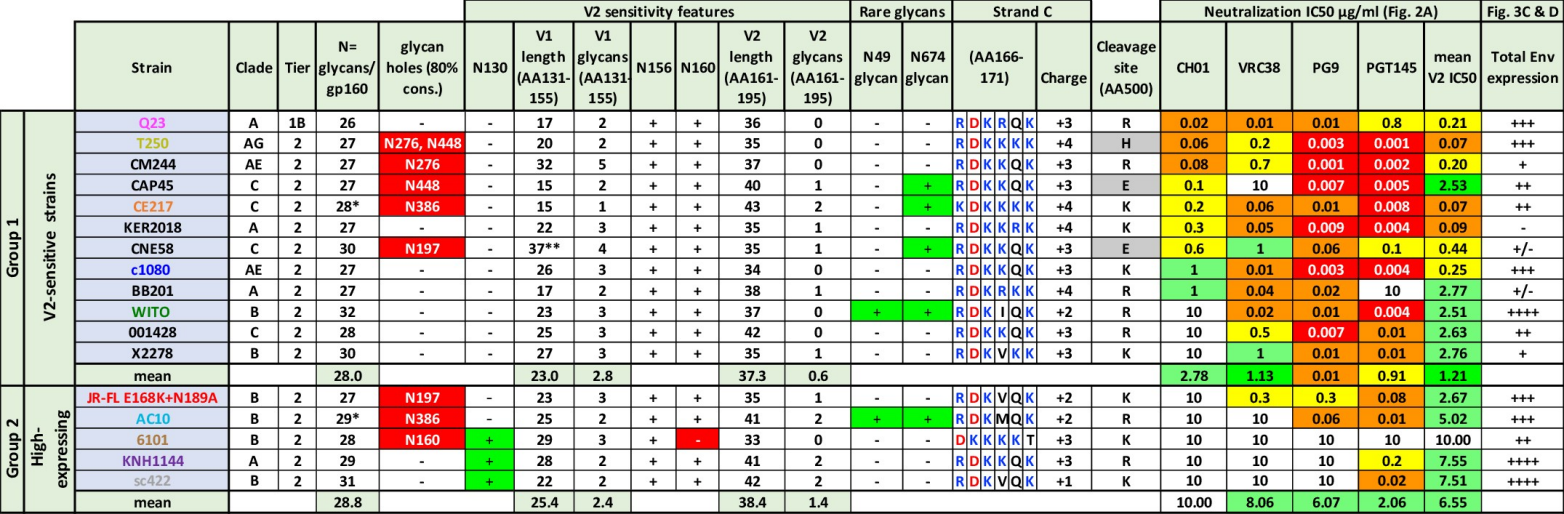
Key features of candidate Env strains. 17 strains were split into group 1 strains (n = 12) that are naturally sensitive to multiple V2 NAbs and group 2 strains (n = 5) that exhibit high membrane trimer expression. Strain names are abbreviated (see Materials and Methods). An asterisk in total glycans/gp160 protomer indicates overlapping sequons in the CE217 and AC10 strains, only one of which can carry a glycan. Glycan holes are listed whenever a ≥80% conserved glycan is absent. V2 sensitivity features are shown, including glycans involved in NAb binding or clashes and loop lengths. A double asterisk for the CNE58 V1 loop denotes a possible internal hairpin disulfide loop (S1 Fig). Rare glycans N49 and N674 are shown. Strand C sequence (AA166-171) is shown with basic residues in blue and acidic residues in red, along with its charge. The residue at position 500 may influence gp120/gp41 processing (gray highlights non-lysine or arginine residues). PV IC50s for V2 NAbs (CF2 assay) (see Fig 2A). JR-FL neutralization data is for the E168K+N189A mutant. Total Env expression, as judged by SDS-PAGE-Western blot (Fig 3C and 3D).

We focused largely on identifying V2-sensitive trimers, due to the relative complexity of this epitope cluster. We could later "knock in" FP sensitivity into V2-sensitive clones. Since few express at levels suitable for vaccine use [92], we took two approaches: i) to screen for high expressing V2-sensitive strains [97] (group 1) and ii) to knock V2 sensitivity into well-expressed trimers (group 2). Key features and sequences of 17 candidate strains are shown in Figs 1 and S1.

**Fig 2:**
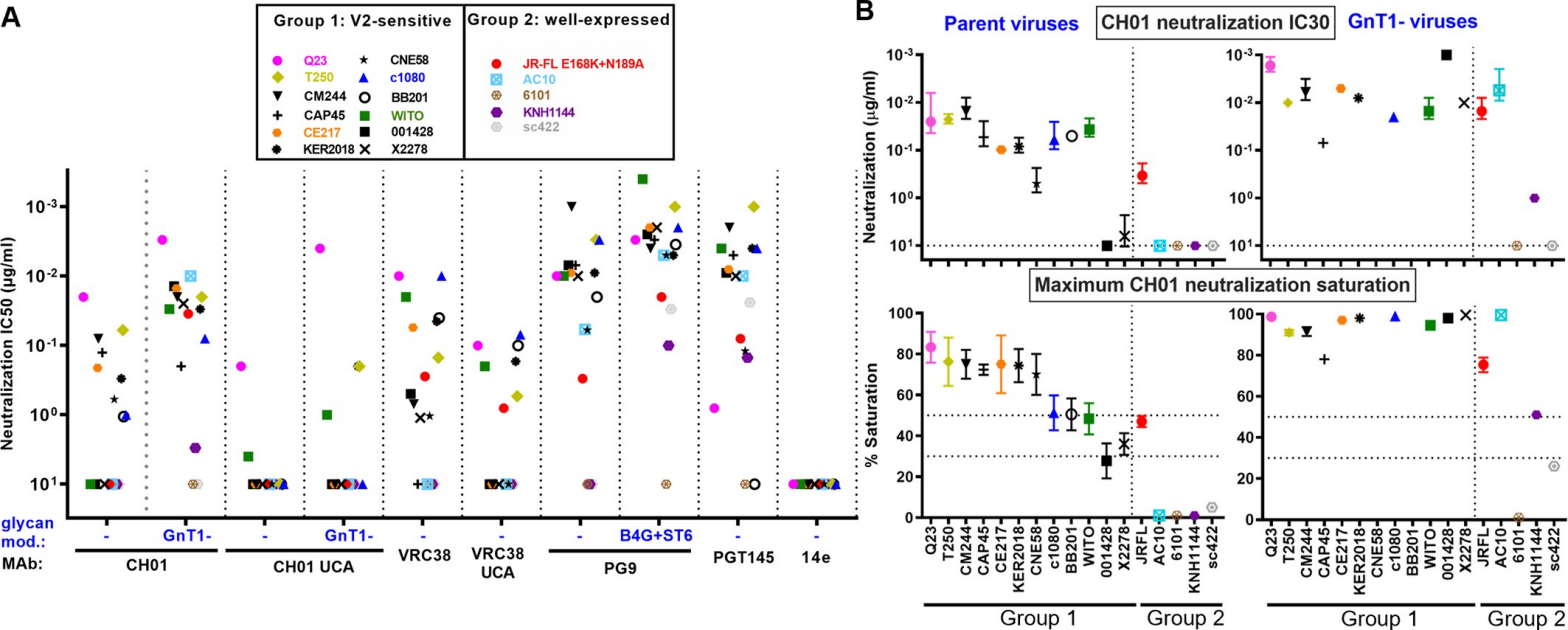
V2 NAb sensitivity of candidate strains. A) MAb IC50s against candidate PVs bearing full-length wild-type (WT) gp160 spikes, except for WITO, AC10, 6101, KNH1144 and sc422, that were gp160ΔCT WT. For CH01 and VRC38, UCA sensitivities are shown. CH01 and PG9 NAb IC50s were measured against PVs bearing Envs with engineered glycans: GnT1- and B4GalT1+ST6Gal1 (abbreviated as B4G+ST6), respectively. GnT1- PV of strains BB201 and CNE58 strains were not infectious. B) CH01 IC30s and % maximum CH01 neutralization saturation in unmodified (left) and GnT1- (right) formats.

**Fig 3:**
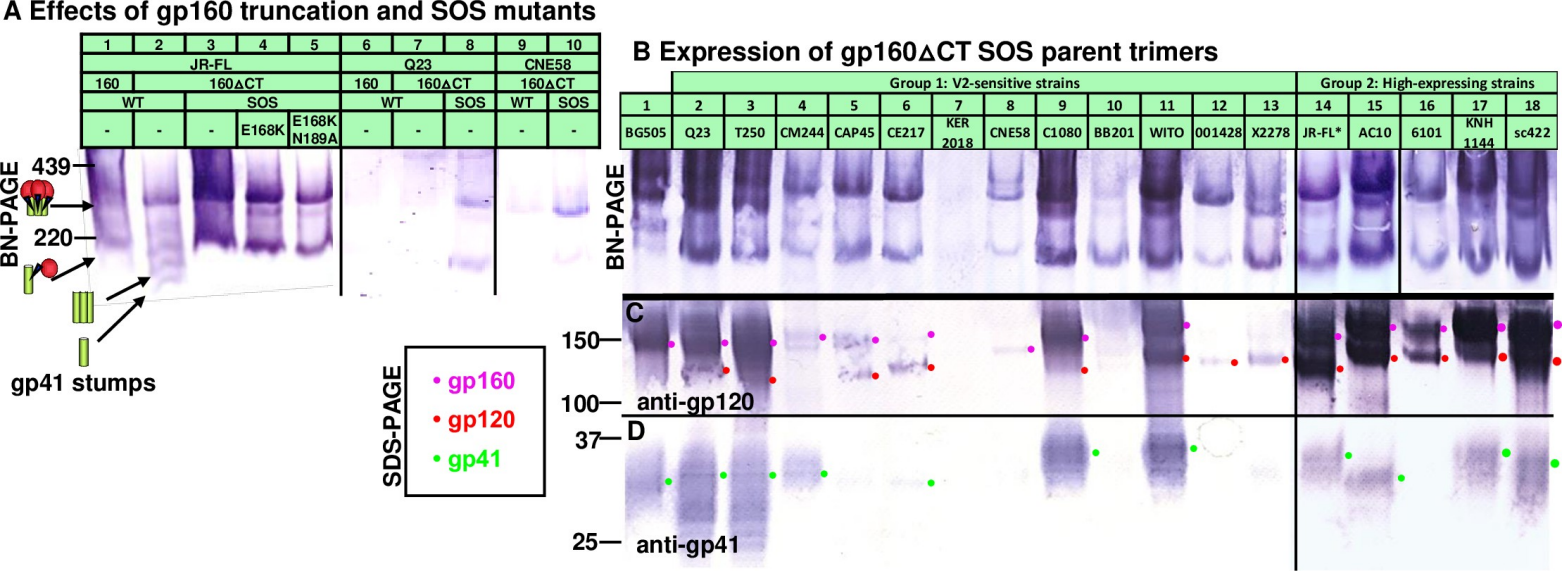
Gp160ΔCT and SOS mutations consistently improve trimer expression. A) VLP trimer expression with or without gp41 truncation (gp160ΔCT) and SOS mutations, probed with anti-gp120 and anti-gp41 MAb cocktail. SOS gp160ΔCT trimer expression of candidate strains visualized by B) BN-PAGE-Western blot and by SDS-PAGE-Western blot, probing with anti-gp120 (C) or anti-gp41 (D) MAb cocktails. All Envs were expressed using robust plasmids (pVRC8400 or pCDNA3.1), except for Q23 in part A lanes 6–8 and BB201 in part B lane 10, for which pCR3.1 was used. pVRC8400 was used to express Q23 in part B, lane 2.

Group 1 strains all carry glycans at N156 and N160, a K/R-rich C strand (residues 166–171), lack the clashing N130 glycan at the V1 loop base (Figs 1 and S1), have few V1V2 glycans and short V1 loop lengths [22,23,55,57]. Group 1 strains Q23, T250, c1080 and WITO were of exceptional interest, as they were both V2-sensitive and well-expressed (Fig 1). Group 2 includes strains that previously expressed well as gp41 cytoplasmic tail-truncated (gp160ΔCT) trimers on VLPs (Fig 1 in [92]). E168K+N189A mutations were shown to introduce V2 sensitivity into the JR-FL strain [14,98], by increasing C strand charge and eliminating an overlapping sequon (S1 Fig). This demonstrates the feasibility of knocking V2 sensitivity into group 2 strains.

Total glycan count varies considerably between strains (Fig 1). Counting non-overlapping sequons in an alignment of 4,582 diverse Env sequences in the LANL database between amino acids 31–674 [99], the median sequons/protomer is 29 (inter-quartile range 28–31). Glycans are important for structure and immune evasion. Structural glycans like N262 [100] impact folding and are therefore relatively conserved (shaded blue in S1 Fig). Other glycans are commonly gained or lost to facilitate NAb escape ([101]; shaded yellow in S1 Fig).

"Glycan holes" occur when conserved glycans are absent (Figs 1 and S1). Filling these holes eliminates off target NAbs and may accelerate the development of bNAbs to the intended target(s) [1,9,11,83,99]. Furthermore, filling glycan holes may assist trimer folding and therefore expression. We note that two strains (WITO and AC10) exhibit rare glycans at positions 49 and 674 that could be important for their high expression (Fig 1). Some strains lack a basic residue at position 500 near the furin processing site (Fig 1), which may adversely impact gp120/gp41 maturation. Below, we explore the effects of modifying these and other Env features to develop a panel of well-expressed immunofocusing membrane trimers.

### V2 sensitivity of trimer panel candidates

We first measured the sensitivities of candidate strains to 4 prototype V2 NAbs (Fig 2A), along with the CH01 Unmutated Common Ancestor (UCA) and a germline-reverted form of VRC38 (both termed ’UCA’ hereafter, for brevity) [24]. For CH01 and its UCA, we also measured IC50s against PV produced in GnT1- cells. We previously showed that CH01 saturation improves against GnT1- PV, presumably as clashes with larger glycans are resolved [14]. PG9 neutralization is more effective against B4GalT1+ST6Gal1- (abbreviated "B4G+ST6") modified PV that increases hybrid glycans and terminal α-2,6 glycan sialylation [14]. Neither of these modifications overtly increase V3 sensitivity, suggesting that trimer folding is not impacted. To track and discard any misfolded trimers, we verified that they were recognized by two V3 MAbs, when activated by sCD4 (S1 Text).

Q23 was the most sensitive strain to MAbs CH01, VRC38 and their UCAs (Fig 2A; [24]) and was also highly PG9-sensitive. Although Q23’s tier 1B classification and moderate PGT145-sensitivity reflect a less compact V2 apex (Fig 1), it is nevertheless 14e-resistant. Surprisingly, WITO, 001428, and X2278 were not neutralized by CH01 (Fig 2A), contrasting previous data [97]. This may be due to CH01’s characteristic "sub-saturating" neutralization and/or that our ’workhorse’ CF2 assay is slightly less sensitive than the commonly used TZM-bl assay [14]. Indeed, CH01 reached IC30s against WITO and X2278 (Fig 2B, top left) and partially neutralized 001428 at 10μg/ml (Fig 2B, bottom left).

In contrast, 13 of 15 GnT1- PVs were CH01-sensitive (Fig 2B, top right; BB201 and CNE58 GnT1- PVs did not infect sufficiently). For 10 of these strains, maximum CH01 saturation was close to 100% (Fig 2B, bottom right) and IC30s were also lower (Fig 2B, compare upper panels). VRC38, PG9 and PGT145 neutralized all of the group 1 strains, except that PGT145 did not neutralize BB201. In some cases, group 2 strains were also neutralized (Fig 2A). Only 6101 was resistant to all V2 NAbs, probably due to its missing N160 glycan (Figs 1 and S1).

Overall, Q23’s exquisite V2 sensitivity supports its use as a V2 priming immunogen. Other CH01-sensitive strains could be useful as boosts. Additional strains may be acquired by engineering changes to increase V2 sensitivity and/or trimer expression, as outlined below.

### *In situ* membrane expression of candidate trimers

Mutations to improve expression were made in gp160 plasmids previously used to make PV of the 17 candidate strains (Fig 1), and BG505 for reference. Expression was examined in BN-PAGE- and SDS-PAGE-Western blots (Fig 3). Membrane trimer expression is improved by truncating gp160 at position 708 (gp160ΔCT), leaving a 3 amino acid gp41 tail (Figs S1 and 3A, compare lanes 1 and 2). Although gp160ΔCT can cause overt V3 sensitivity in some settings [102], the effect is minimal for JR-FL [103]. Previously, we found that high trimer expression can be achieved by co-transfecting Env plasmids with MuLV Gag (Figure S1 in [24]) and Rev plasmids (used when Env is not codon-optimized). The SOS mutation (501C+605C) further improves JR-FL trimer expression compared to WT, probably because gp120 shedding is eliminated, evidenced by the lack of gp41 stumps (Fig 3A, compare lanes 2 and 3). E168K and E168K+N189A variants were also well-expressed (Fig 3A, lanes 4 and 5). Gp160ΔCT SOS mutants of strains Q23.17 and CNE58 were also better expressed than their full-length gp160 and/or WT counterparts (Fig 3A, lanes 6–10).

BG505 trimers expressed modestly well (Fig 3B, lane 1), and consisted largely of uncleaved gp160 (Fig 3C and 3D, lane 1). Q23 SOS gp160ΔCT expression by the pVRC8400 plasmid (Fig 3B–3D, lane 2) was far higher than by the pCR3.1 plasmid (Fig 3A, lane 8). The pCR3.1 plasmid may also account for poor BB201 Env expression (Fig 3B–3D, lane 10). For all other strains, expression plasmids pVRC8400 or pCDNA3.1 were used. Group 1 trimers T250, c1080, and WITO expressed well, (Fig 3B–3D, lanes 3, 9 and 11). CM244, CAP45, CE217, CNE58, 001428 and X2278 trimers expressed modestly (Fig 3B–3D, lanes 4, 5, 6, 8, 12 and 13), while KER2018, expressed poorly (Fig 3B–3D, lane 7).

In contrast, all 5 group 2 SOS gp160ΔCT mutants expressed high levels of trimer (Fig 3B, lanes 14–18). Gp160 and gp120 expression was high in all cases (Fig 3C, magenta and red dots). Corresponding gp41 bands were also observed in all but 6101. Overall, these blots reveal vast differences in Env expression between strains and that poor expression is common [92]. The group 2 strains and the 4 group 1 strains that express high levels of gp120/gp41 trimers (Q23, T250, c1080 and WITO) are of particular interest for follow up. Next, we tried to modify the most promising strains to improve V2 sensitivity and/or trimer expression.

### JR-FL modifications

We first modified our prototype vaccine strain, JR-FL, hoping to improve V2 and FP sensitivity. The E168K+N189A mutant is sensitive to VRC38, PG9 and PGT145 (Fig 1) and is partially sensitive to CH01 (Fig 2B). V2 sensitivity might be improved by removing local clashing glycans and/or by increasing strand C’s basic charge. These modifications were made in the JR-FL SOS gp160ΔCT background. We initially compared WT and SOS PV NAb sensitivities. SOS PV infection can proceed after receptor engagement by adding traces of reducing agent to break the gp120-gp41 disulfide bond [104]. Like its WT counterpart, the SOS PV was V3-resistant and PG9 and VRC38-sensitive (S2A Fig). However, CH01 saturation was notably greater and an IC50 was measurable (S2A Fig), reinforcing our decision to use the SOS mutant.

**Fig 4:**
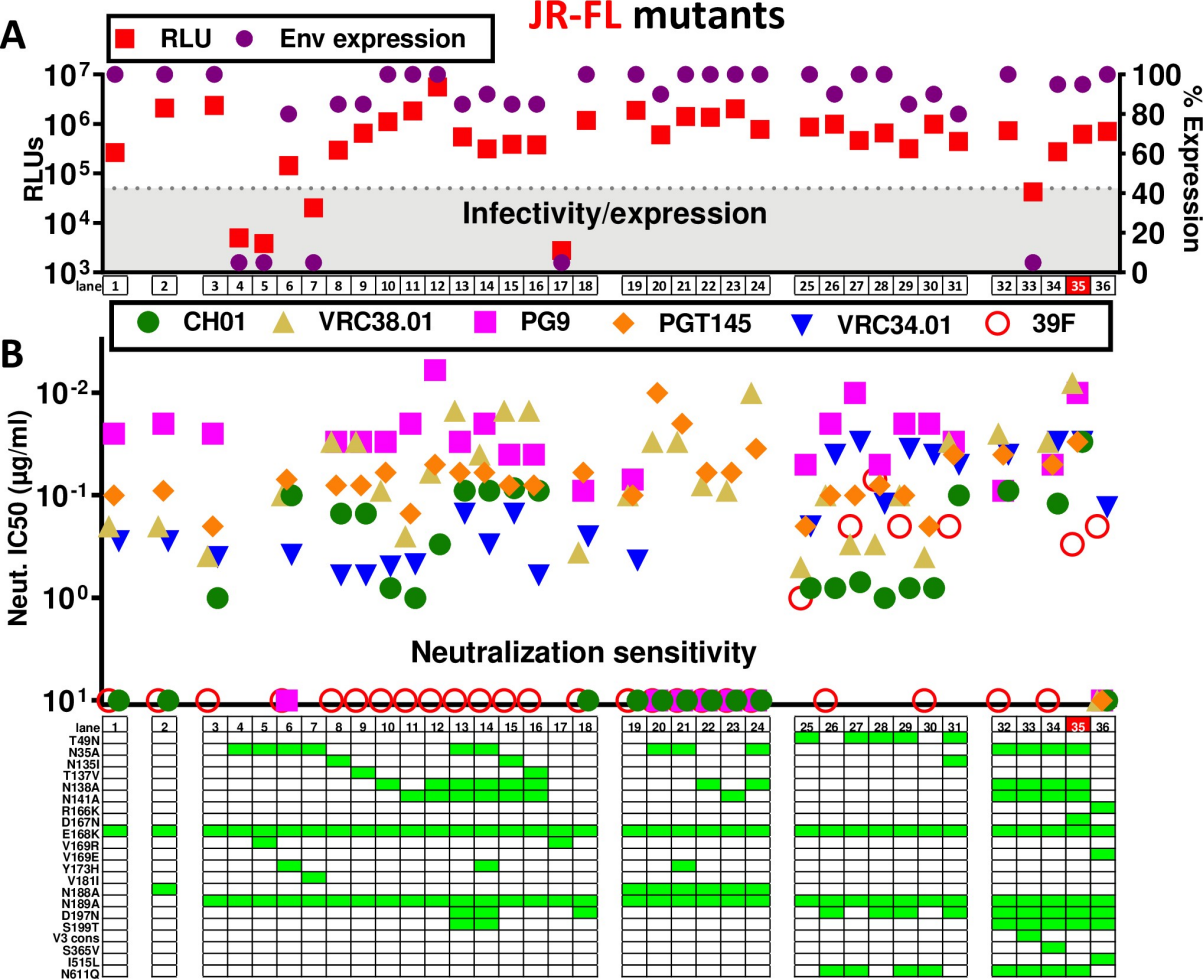
Effect of mutations on JR-FL SOS gp160ΔCT trimer expression, infectivity and MAb sensitivity. Effect of mutations on A) JR-FL gp160ΔCT SOS trimer infectivity, and total Env expression (quantified by SDS-PAGE-Western blot), and B) MAb sensitivity. The best mutant is highlighted in red (lane 35). The V3 consensus (V3 cons) mutant in lane 33 included H310R+R315Q+T319A+E322D mutations.

We next investigated the effects of removing the overlapping V2’ sequons N188 and N189 alone or together. Despite only moderate effects on V2 IC50s (Fig 4, lanes 1–3), both single mutants improved PG9 saturation, while the double mutant showed reduced PG9 sensitivity and saturation (Figs S2B and 4B, lane 19). Unlike the E168K, E168K+N188A and E168K+N188A+N189A mutants, E168K+N189A was modestly CH01-sensitive (Fig 4B, lanes 1–3, 19). We therefore used JR-FL E168K+N189A as a "parent" clone to overlay further mutations.

V2 NAb sensitivity typically depends on glycans N156 and N160 and basic (K/R) residues in strand C [55,57,62,63,98] (S3 Fig). K168 and K171 are V2 NAb "anchor" residues [24,62]; K/R residues at 166, 169 and 170 also contribute to strand C’s positive charge (S3 Fig). Our JR-FL E168K+N189A mutant contains these anchor residues and a net charge of +2, which is somewhat lower than many V2-sensitive strains that exhibit K/R at position 169 of strand C (Fig 1).

### V1 and V2 loop glycan deletions

We initially sought to maximize JR-FL’s CH01 sensitivity, hoping to concomitantly boost sensitivity to other V2 NAbs. We first removed potentially clashing glycans at positions 135, 138 and 141 at the V1 loop base (S1 Fig). The N135A mutant dramatically reduced PV infectivity and trimer expression (Fig 4A, lane 4), suggesting a folding problem. To try to obtain an infectious N135A mutant, we combined it individually with 3 V2 substitutions commonly found in other strains (S3 Fig). A V169R mutation would increase strand C positive charge, possibly improving V2 sensitivity. However, neither the N135A+V169R double mutant nor the V169R alone were infectious or expressed trimer (Fig 4A, lanes 5 and 17). Y173 and Y177 of the V2 loop (S3 Fig) may interact with residues N300 and K305 at the V3 loop base to influence trimer folding [105]. H173 is also common (S3 Fig). N135A+Y173H restored some PV infectivity, albeit not to the level of the parent (Fig 4A, lane 6). Moreover, CH01 sensitivity and saturation was dramatically improved (Figs S4A and 4B, lane 6). Conversely, PG9 sensitivity was eliminated (Fig 4B, lane 6). Since the Y173H mutant alone did not affect CH01 sensitivity (S4A Fig), the increased CH01 sensitivity of the N135A+Y173H mutant is likely due to the N135A mutation. N135A+V181I also showed improved infectivity, but was insufficient to measure neutralization (Fig 4A, compare lanes 4 and 7). As alternative solutions, mutants N135I and T137V both improved CH01 and VRC38 sensitivity, but unlike N135A+Y173H, PG9 sensitivity was retained (Fig 4B, compare lanes 3, 8 and 9). Compared to N135A+Y173H, these mutants were slightly more VRC38-sensitive, but slightly less CH01-sensitive (Fig 4B, compare lanes 6, 8 and 9). Overall, N135I and T137V mutations were preferable to N135A+Y173H (better expression and infectivity).

**Fig 5:**
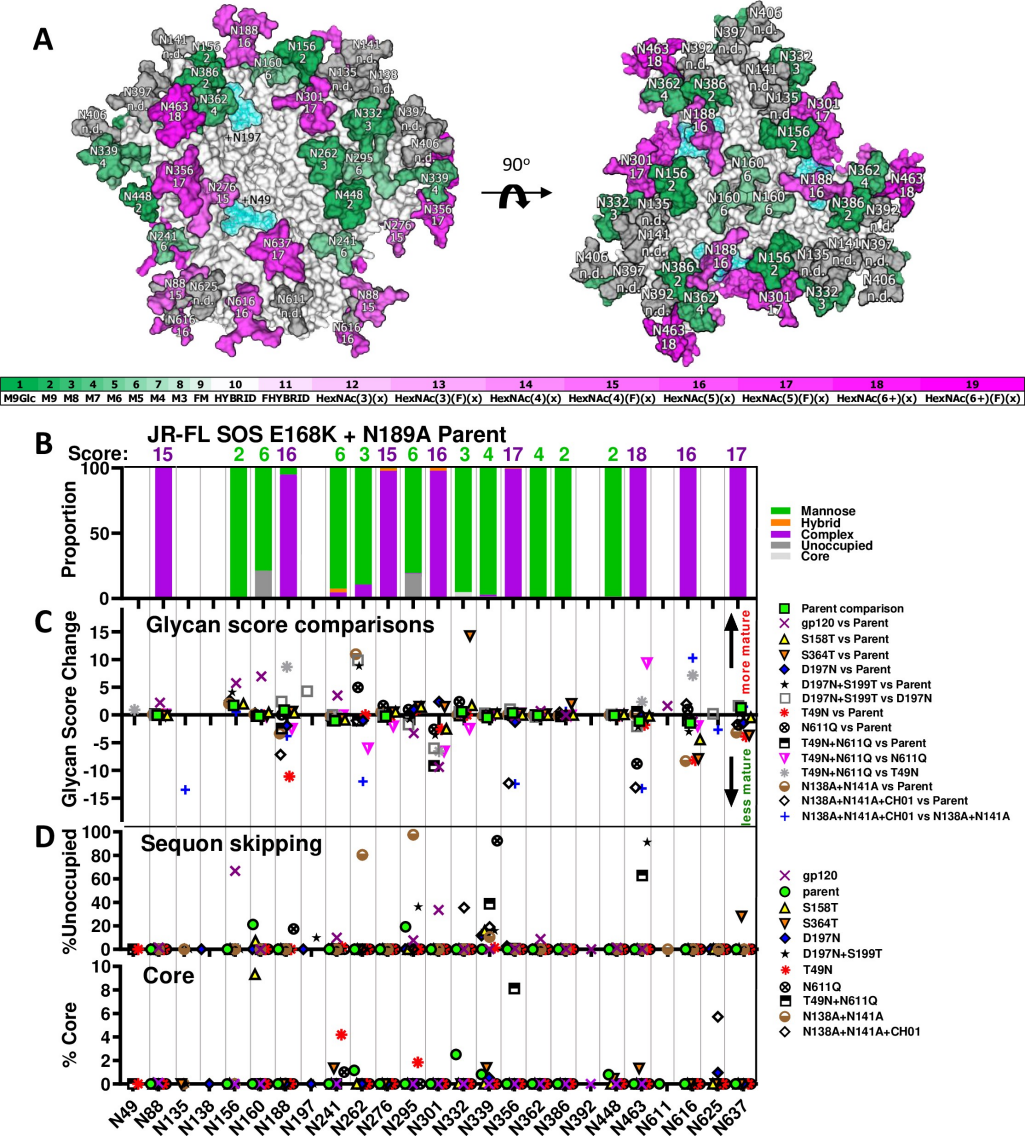
Effects of mutants on JR-FL membrane trimer glycan maturation and occupation. Related to S1 Data and analysis and Figs S5 and S6. A) In a trimer model (pdb: 6MYY), each glycan is numbered according to the prototype HXB2 strain (see S1 Fig) and is given a maturation score, derived from LC-MS analysis of parent JR-FL SOS E168K+N189A VLPs (S1 Data and analysis). Glycans are colored in shades of green (high mannose) or magenta (complex), according to their score. Untrimmed high mannose glycans are dark green. Trimmed high mannose glycans are shown in lighter hues of green. Heavy complex glycans are shown in dark magenta, whereas smaller complex glycans are shown in lighter hues of magenta. Some glycans, rendered in gray, were not resolved in the JR-FL parent and therefore have no score (not done; n.d.). Glycans at positions N49 and N197 are modeled as blue translucent masses. B) Glycan identity and scores at each sequon in JR-FL SOS E168K+N189A VLPs determined by LC-MS. Glycans were assigned scores by their degree of maturation (S5 Fig). C) Changes in glycan scores at each position between sample pairs. A negative score implies a shift to less mature glycan and *vice versa*. Data are only shown at positions where a glycan was detected in >10% of the equivalent peptides of both samples in each pair. Score difference calculations are shown in S1 Data and analysis and are modeled in S6 Fig. D) Sequon skipping and core glycans.

N138A did not impact expression or infectivity (Fig 4A, compare lanes 3 and 10), but modestly improved CH01 sensitivity and saturation, albeit not to the extent of N135 glycan knock out mutants (Figs S4A and 4B, compare lanes 3, 6, 8, 9 and 10). VRC38 and PGT145 sensitivities were also higher (Fig 4B, lanes 3 and 10). The greater impact of N135A than N138A on V2 NAb sensitivity is consistent with its closer structural proximity to the core N156 and N160 glycans (Fig 5A).

N141A had little effect on any parameter (Fig 4, compare lanes 3 and 11). However, a previous report suggested that this sequon is only 50% occupied by glycan [79], so it is perhaps not surprising that removing the glycan has little effect. Incomplete N141 occupation may be due to spatial competition with N138 (Fig 5A). If so, N141 occupation may increase if N138 is absent. Accordingly, a N138A+N141A double mutant exhibited improved CH01, VRC38 and PG9 sensitivity compared to N138A alone (Figs S4A and 4B, lanes 10–12). Infectivity and trimer expression were comparable to the parent (Fig 4A, lanes 3 and 12).

To further improve CH01 sensitivity, we removed all 3 V1 glycans in a N135A+N138A+N141A mutant. To compensate for the loss of 3 glycans, at the same time, we filled the N197 glycan hole, using a D197N+S199T mutation [1]. Located at the base of the V2 loop, the N197 glycan could impact V2 NAb binding (Fig 5A). The N135A+N138A+N141A+D197N+S199T mutant was well-expressed and infectious. An additional Y173H mutation was not necessary (Fig 4A, lanes 13 and 14). Thus, either the removal of other V1 glycans and/or the addition of the N197 glycan compensated for the N135A folding defect (Fig 4A, lane 4). The triple ΔV1 glycan mutant was marginally more V2-sensitive than the N135 single glycan mutants (Figs 4B, compare lanes 6, 8, 9 to lanes 13 and 14 and S4A). Since the N197 glycan lies at the edge of the V2 apex (modeled in cyan in Fig 5A), it is possible that the D197N+S199T mutant directly impacts V2 NAb sensitivity. Compared to the parent, the D197N mutant alone exhibited higher PGT145 sensitivity but weaker or no PG9 and CH01 sensitivity, respectively (Fig 4B, lanes 3 and 18). Thus, the D197N glycan alone does not account for the high V2 sensitivity of the N135A+N138A+N141A+D197N+S199T mutant. Triple V1 glycan knockout mutants using N135I and T137V were also highly V2-sensitive, (Fig 4B, lanes 13–16). These latter mutants do not contain a D197N mutation, further suggesting that most V2 sensitivity gains stem from V1 glycan deletion, not N197 glycan addition.

We next tested if the above mutants might be further augmented by removing *both* the N188 and N189 glycans (Fig 4B, lanes 19–24). In this context, N135A showed high VRC38 and PGT145 sensitivity (Fig 4B, compare lanes 6 and 21). However, these mutants were neither CH01- nor PG9-sensitive. Therefore, the reduced PG9 saturation of the E168K+N188A+N189A mutant noted above (S2B Fig), was exacerbated by V1 glycan removal, suggesting that the N188 glycan is required for broad V2 sensitivity.

### Improving FP NAb sensitivity

To try to improve FP NAb sensitivity, we removed the N611 clashing glycan [8]. We also knocked in the rare N49 glycan that is carried by well-expressed strains WITO and AC10 (S1 Fig), hoping to boost JR-FL trimer expression. Modeling places the N49 glycan in between the N276 and N637 glycan sites (Fig 5A). Its proximity to N637 led us to wonder if it might impact the local glycan network that regulates sensitivity to FP NAbs like VRC34 that contact N88 glycan and clash with N611 glycans. We therefore toggled the N49 and N611 glycans, with or without D197N. T49N and N611Q glycan mutants did not appreciably impact infectivity and trimer expression (Fig 4A, lanes 25–31). N611Q improved VRC34 sensitivity, while T49N did so to a lesser extent (S4B Fig). T49N+N611Q was only marginally more VRC34-sensitive than N611Q alone (Fig 4B, lanes 3, 25, 27 and 30). Notably, T49N mutants were all modestly 39F-sensitive (Figs S4C and 4B, lanes 25, 27–29, 31). Overlaying a N135I mutation increased V2 sensitivity (Fig 4B, compare lanes 28 and 31). Thus, despite causing partial V3 exposure, the N49 glycan did not perturb V2 apex epitope integrity and modestly improved VRC34 sensitivity.

### Effects of mutations on glycan maturation and occupation

To better understand the basis of the effects of JR-FL mutants observed in Fig 4, we assessed glycan occupation and maturation of these mutants by glycopeptide in-line liquid chromatography mass spectrometry (LC-MS) [68,81]. Each glycan type was given a score from 1 to 19, depending on the average maturation state (S5 Fig and S1 Data and analysis). Thus, the high mannose glycan, M9Glc, is given a score of 1, while the most highly branched and fucosylated complex glycan HexNAc(6+)(F)(x) has a score of 19. Glycan scores of parental E168K+N189A trimers are modeled in Fig 5A. The scores and diversity at each site are summarized in Fig 5B. The nature of glycans at each site generally match a previous report that categorized JR-FL PV Env glycans by another method [79], although the N160 and N386 glycans were mostly high mannose in our hands but were complex by the other method.

We next evaluated glycan score differences at each site in pairs of samples. Score changes were recorded in a dot plot (Fig 5C) for sites that were >10% occupied by glycans (excluding core glycans, i.e., truncated glycan structures smaller than M3) in both samples. Score differences for each pair are modeled (S6 Fig). Sequon skipping and core glycans are shown in Fig 5D.

We first compared two preparations of JR-FL SOS E168K+N189A VLP trimers (’parent’) analyzed on different days to gauge sample and assay variation (Figs S5 and S6 and S1 Data and analysis). Minor differences in glycan maturation were observed, e.g., at position N156 (Figs 5C and S6). Sequon skipping was rare and varied between samples, occurring at positions N156 (0.87%) and N362 (0.4%) in one sample and at N160 (21.24%) and N295 (19.24%) in the other (S1 Data and analysis, average % skipping shown in Fig 5D). Glycan core was found occasionally (~5% or less) at 4 sites in one sample, but not at all in the other (S1 Data and analysis; average % core shown in Fig 5D). Several sequons could not be assigned a glycan (e.g., N135 and N138), as their proximity made it difficult to isolate peptides with only one glycan.

A comparison of ’parent’ trimers and monomeric JR-FL gp120 revealed that glycan types (i.e., high mannose or complex) were similar at many positions (S6 Fig). However, gp120 monomer glycans were more differentiated at positions N88, N156, N160 and N241, perhaps reflecting the greater access to glycan processing enzymes (Fig 5C). Similar observations were made in a comparison of BG505 SOSIP and gp120 [106]. Conversely, glycans N295 and N301 were less mature, which, as for SOSIP, may be because the relatively fast Env production rate reduces contact time with glycan processing enzymes (S6 Fig).

Sequon skipping was common in gp120: 8 out of 14 sequons were partially unoccupied (Fig 5D and S1 Data and analysis). Of these, N156 was most frequently skipped (66.7%), followed by N301 (33.5%) and N362 (8.9%). As noted above, N156 and N362 were also occasionally skipped in membrane trimers, albeit to a far lesser extent. Both sequons have a serine at the 3^rd^ position, i.e., NXS (S1 Fig).

Since NXT is a better substrate for glycan transfer [84,107], we made S158T and S364T mutants. Glycopeptide LC-MS revealed that S158T mutant glycosylation was broadly similar to the parent, with score changes <Δ5 (Figs 5C and S6 and S1 Data and analysis). Differences in both directions were observed, e.g., at N156 and N301. Modest sequon skipping and core glycans at N160 could be a direct consequence of the adjacent S158T mutation, as in BG505 SOSIP [84]. Skipping also occurred at N339 (Fig 5D and S1 Data and analysis). S364T dramatically increased N332 glycan differentiation and caused skipping at N339 and N637 (Figs 5C and 5D and S6). Some of the above effects were distal from the two mutation sites, suggesting allosteric ’glycan network’ effects (Fig 5A). In both cases, gp41 N616 and N637 glycans were less mature (Fig 5C). Collectively, our findings suggest that these two mutants disturbed conserved serine residues (S1 Fig), resulting in modest structural changes.

The D197N mutant completely knocked in the N197 glycan (S1 Data and analysis, Figs 5C and 5D and S6). N301 maturation was modestly increased, perhaps due to its proximity (Figs 5A and S6). N160 and N637 sequons were both fully occupied, unlike the S158T and S364T mutants, respectively. However, like S158T and S364T, some skipping occurred at N339 (Fig 5D and S1 Data and analysis). Overall, D197N was well-tolerated compared to the S158T and S364T (Figs 5C and S6).

A sequon-optimized D197N+S199T mutant was inferior. It only filled the N197 site to ~90% efficiency and caused glycan holes elsewhere, most notably at N463 (~91% skipped) and N295 (~36% skipped) (Fig 5D). N262 partly toggled to complex, whereas N301 became immature (S6 Fig). Overall, D197N+S199T was poorly tolerated, like S158T and S364T, affecting distal glycans in a global "ripple" effect, further cautioning against mutations at conserved positions (S1 Fig).

T49N successfully added a complex glycan that led to decreased maturation at positions N188, N301, N616, and N637 (Figs 5C, S5 and S6), presumably due to overcrowding. This contrasted sharply with the mild effects of D197N. The reduced gp41 glycan complexity of the T49N mutant is consistent with SDS-PAGE-Western blot analysis (S2 Text). We could not obtain glycopeptide data for N611 that might have given insights into how the N49 glycan improves VRC34 sensitivity. While N611 is not close to the N49 (Fig 5A), it is possible that smaller glycans at the other gp41 sites provide space for the N611 glycan to move aside for VRC34 binding. Our model suggests that some effects are localized (Fig 5A), but others (e.g., N188) are distal, suggesting a global conformational change, consistent with partial V3 MAb sensitivity (Figs 4B and S4C).

N611Q led to increased N262 glycan maturation, decreased N463 glycan maturation and skipping at N188 and N339 (Fig 5D). T49N+N611Q led to reduced N301 maturation (Figs 5C and S6) and skipping at N339 and N463 (Fig 5D). Reduced N301 maturation of the single T49N and N611Q mutants appeared to be amplified in the double mutant. However, other effects in the single mutants were absent in the double mutant, suggesting that N49 knock in partially compensates for N611 glycan loss (S6 Fig and S2 Text). Comparing the double mutant to its component single mutants again highlighted differences at N188, N262, N301, N463 and N616 (S6 Fig), although the patterns did not resemble those above, implying unpredictable and subtle effects on trimer folding.

Analysis of N138A+N141A revealed that the N135 glycan is complex in the absence of these neighboring glycans (S5 Fig). Significant skipping at positions N262 and N295 was observed, along with some at N339 (Fig 5D). The small amount of glycan detected at position N262 was far more mature than on the parent (Fig 5C). Although this mutant was infectious (Fig 4A, lane 12), since this glycan is structurally important, its absence could cause some misfolding [100]. Glycan maturation differences were also observed at N156, N188, N616 and N637.

N138A+N141A membrane trimers complexed with CH01 exhibited radical differences at some positions: a shift to high mannose at N135 and N188 may help to minimize clashes at the binding site (Figs 5A, and S5 and S6) [14]. However, N356 and N463 glycans were also less mature, despite being distal from the CH01 epitope. Intriguingly, N262 and N295 glycans were efficiently detected in CH01-bound sample, even though both were largely skipped in the unbound sample (S1 Data and analysis). Conversely, N332 exhibited more skipping and N616 was more complex in the CH01-bound sample. Thus, it appears that rare glycan species were found in CH01-trimer complexes that were not detected in uncomplexed trimers. Since CH01 neutralizes the N138A+N141A mutant to a maximum of only ~75%, it appears to bind only to trimers where N135 and N188 glycan clashes are minimal. This trimer population carries other unusual glycans at distal sites that may further improve CH01 binding or are inextricably linked with high mannose glycans at the epitope.

Overall, this data reveals that outer domain glycans (N156-N339) are prone to maturation changes, while inner domain glycans N88, N356-N448 are largely static. Sequon skipping is also more common at some outer domain glycan sites, particularly N339. Importantly, our findings provide some guidance regarding how well our engineering efforts are tolerated.

### Final JR-FL mutants

A final set of JR-FL mutants was made based on the best mutants so far, starting with the triple V1 glycan deletion mutant in Fig 4B, lane 13. Overlaying N611Q improved VRC34 sensitivity, as expected, while V2 NAbs were largely unaffected except for a modest loss of PG9 sensitivity (Fig 4B, lane 32). Previous studies suggested that modifying V3 sequence [108] and an S365V mutation [109] may improve V2 NAb sensitivity. However, trimers mutated with a global V3 consensus sequence (lanl.gov) did not express efficiently, and S365V had little effect (Fig 4, lanes 33 and 34). This suggests that cognate V2-V3 sequences are important for folding and that the effect of S365V is context-dependent.

Highly basic C-strands may initiate V2 NAb lineages via electrostatic interactions [37,70,110]. However, a V169R mutant to render the JR-FL C-strand more like many V2-sensitive group 1 strains (Fig 1) was misfolded (Fig 4B, lane 17). A D167N mutant provides another way to increase strand C’s positive charge (Fig 4B, lane 35), as found in some V2-initiating sequences [70]. This further increased CH01 sensitivity (Figs S4A and 4B, lane 35). Excitingly, this mutant was broadly sensitive to all 4 V2 NAbs (Fig 4B, lane 35 marked in red). However, it was also somewhat V3-sensitive (S4C Fig) and PGT145 saturation was somewhat reduced (S4D Fig). Taken together, the increased V3 sensitivity and reduced PGT145 saturation suggests a slightly more ’open’ trimer apex. Despite its improved CH01-sensitivity, this mutant was still resistant to the CH01 UCA (S4A Fig). A further increase C-strand positive charge via D167K mutation reduced V2 sensitivity (S4A and S4D Fig). This is perhaps not surprising, given that D and N are the only permissible residues at this position (S3 Fig).

During V2 NAb ontogeny in natural infection, the C-strand may become more neutral, as the virus attempts to escape NAbs. In turn, V2 NAbs evolve to be less dependent on electrostatic charges and depend more on V2 "anchor" residues [37,55,70,110]. To mimic the "escape" phenotype of such "late" viruses, we made an R166K+V169E variant. We also added back the V1 and N611 glycans and modified the FP sequence to the second most common variant [8]. Including these changes in boosts could help V2 and FP NAbs evolve to tolerate sequence variations and navigate glycans. However, none of the V2 NAbs neutralized this variant, most likely because V169E eliminates V2 binding completely. Nevertheless, many other mutants in Fig 4B provide multiple options to increase V2 stringency in boosts without eliminating V2 sensitivity altogether. Conversely, VRC34 neutralized I515L comparable to other mutants that retain the N611 glycan, suggesting that it tolerates this FP sequence variation.

Finally, we investigated approaches to improve JR-FL Env processing at the lysine rich gp120/gp41 junction (S3 Text). While we were unable to improve cleavage efficiency by mutation or furin co-transfection, data suggest that 500K/R "repair" mutation should be used whenever another residue is found at this position.

### An alternative PV neutralization assay for poorly infectious clones

**Fig 6:**
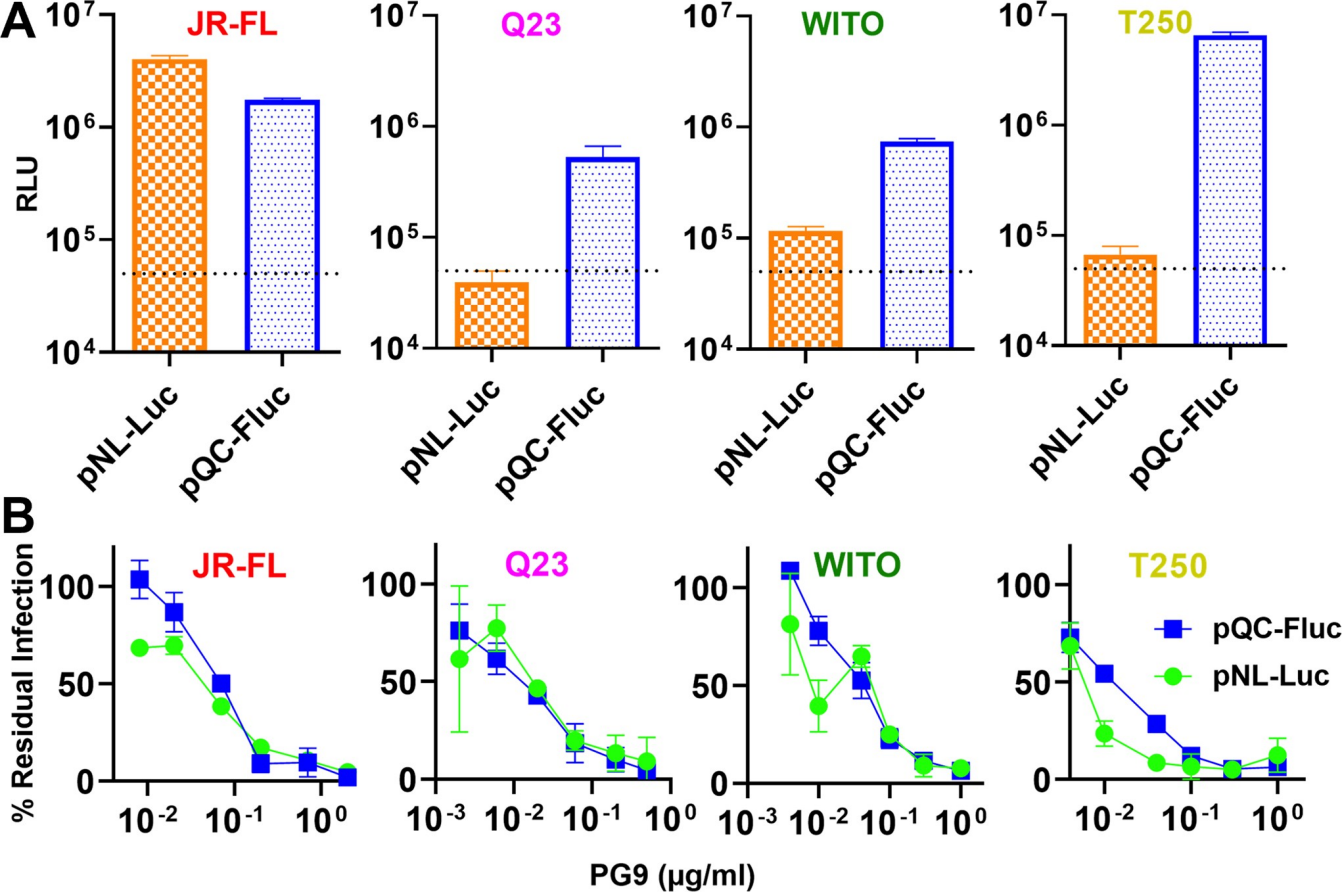
Comparison of pNL-Luc and pQC-Fluc assays for HIV pseudovirus infectivity and neutralization sensitivity. A) The infectivities of JR-FL E168K+N189A, Q23 D49N+N611A, WITO and T250 SOS gp160ΔCT PVs, produced using pNL-Luc or pQC-Fluc plasmid sets were compared in CF2.CD4 CCR5 cells. The dotted line marks an arbitrary cutoff for infection sufficient to measure neutralization. B) Comparative PG9 sensitivity of the same PV in both assays.

Our standard neutralization assay uses pNL-LucR-E- and an Env plasmid to make PV for infection of CF2.CD4.CCR5 cells. Using this assay, Q23 SOS gp160ΔCT PV infection was low and close to our arbitrary cutoff of 50,000 relative light units (RLUs), where neutralization becomes difficult to distinguish from background. The "gold standard" TZM-bl protocol cannot be used for SOS PV infection, as it involves overlaying cells on virus-antibody mixtures, which is incompatible with our requirement to wash cells after briefly exposing them to 5mM DTT to break the SOS bond of spikes attached to cellular receptors, allowing infection to proceed [104]. We therefore sought a different protocol that uses pre-attached cells. We adapted a PV assay previously reported for coronaviruses, in which viral budding is driven by MuLV GagPol and luciferase is carried by plasmid pQC-Fluc [111]. PVs made this way mediated elevated infection versus the NL-Luc assay for poorly infectious Q23, WITO and T250 SOS PVs (Fig 6A), which could be related to the more robust particle budding by MuLV GagPol [24]. However, JR-FL SOS PV infection (already high in the NL-Luc assay), was slightly lower. To check if NAb sensitivity was impacted, we compared PG9 neutralization of the four viruses in both assays. The NL-Luc assay resulted in high error bars compared to the pQC-Fluc assay, most notably for Q23 and WITO (Fig 6B). Nevertheless, PV PG9 sensitivities were comparable, suggesting that the pQC-Fluc assay is a reasonable substitute whenever infectivity is too low in the NL-Luc assay.

### Q23

**Fig 7:**
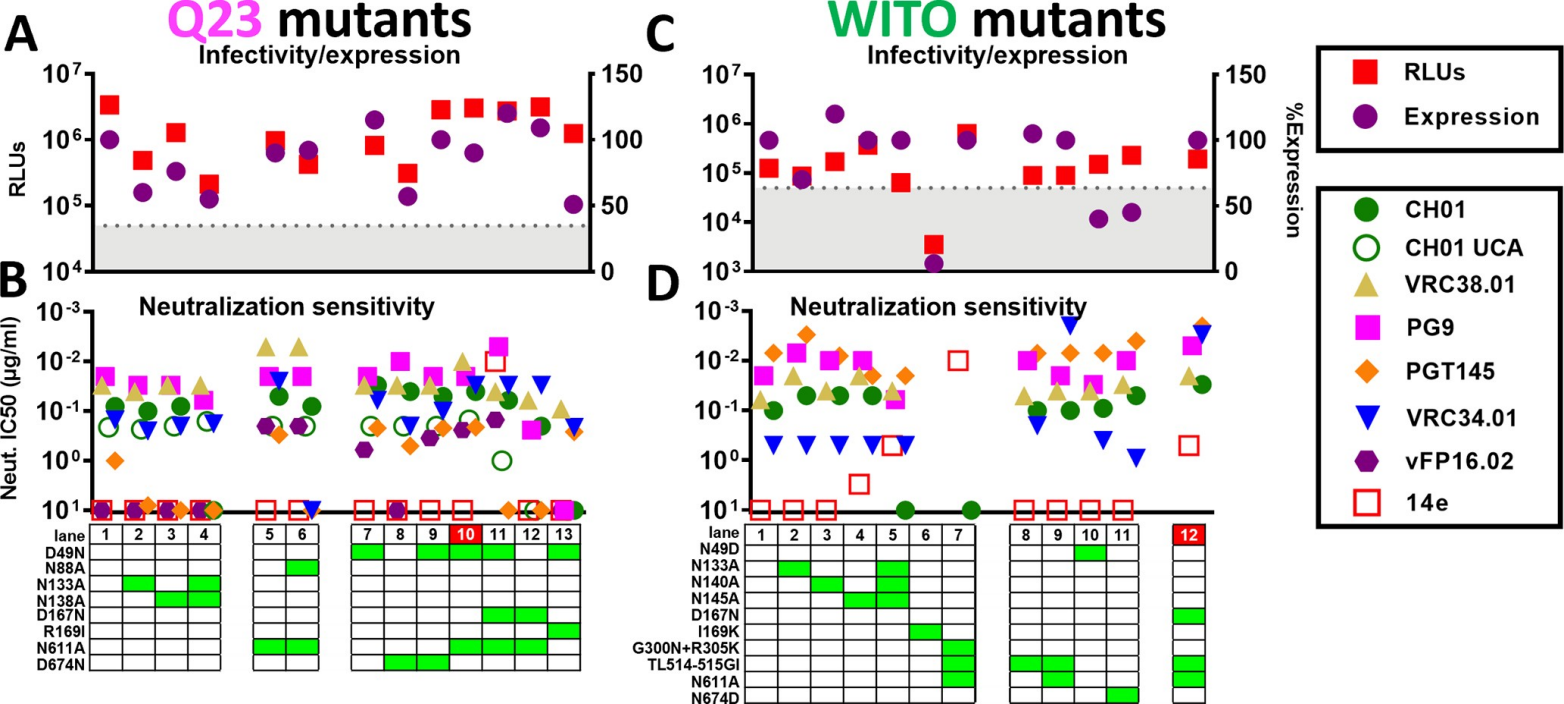
Effects of Q23 and WITO SOS gp160ΔCT mutations on trimer expression, infectivity and MAb sensitivity. Effect of mutations on A) Q23 and C) WITO gp160ΔCT SOS trimer infectivity (by the pQC-Fluc assay) and total Env expression (by SDS-PAGE-Western blot). B) and D) MAb sensitivities of mutants.

Q23 is highly CH01 UCA-sensitive (Fig 2) and expresses well (Fig 3B–3D), hence may be ideal for V2 NAb priming. It is generally V2-sensitive (Figs S7A and 7B, lane 1). To further increase V2 sensitivity, we removed V1 glycans at positions N133 and N138 alone and together. These mutants reduced infectivity and expression (Fig 7A, lanes 1–4), but had little effect on V2 NAb or CH01 UCA sensitivities, except that the weak PGT145 sensitivity of the parent virus was lost (Figs S7C and 7B, lanes 1–4).

To try to increase FP NAb sensitivity, we tested the effect of N611A alone and together with N88A. As for JR-FL, N611A mutation improved VRC34 sensitivity. Vaccine-elicited FP NAb vFP16.02 also neutralized this mutant (Fig 7B, lane 5). When N88A was overlaid, vFP16.02 was still able to neutralize, but VRC34 sensitivity was lost, consistent with VRC34’s requirement for N88 glycan (Fig 7B, lane 6).

We next examined the effects of knocking in N49 and N674 glycans, as found on well-expressed strains WITO and AC10 (Fig 1). Considering Q23 trimer’s low total glycan number (Figs 1 and S1), additional glycans might assist trimer folding. D49N slightly improved expression, whereas D674N reduced expression (Fig 7A, lanes 7 and 8). Together, there was no net effect on expression (Fig 7A, lane 9). Notably, D49N knocked in vFP16.02 sensitivity and increased VRC34 sensitivity (Fig 7B, compare lanes 1 and 7), as for JR-FL (Fig 4). PGT145 and CH01 sensitivities were also improved (Fig 7B, compare lanes 1 and 7–9).

D49N+N611A expressed well and was robustly VRC34- and vFP16-sensitive (Fig 7B, lane 10). CH01 sensitivity was moderately higher than the parent (S7A Fig), whereas CH01 UCA sensitivity was unaffected (Figs S7B and 7B, lane 10). 14e saturation also increased, although it did not achieve an IC50 (S7D Fig). Overall, this further suggests that the N49 glycan opens the trimer slightly to expose V2 and V3 targets. To further increase V2-sensitivity, we overlaid the D167N mutant. However, this reduced V2-sensitivity and caused overt V3 sensitivity (Figs 7, lane 11 and S7A, S7B and S7D). Since N49 and D167N may both modestly increase V3 sensitivity, together they may lead to overt V3 sensitivity. We therefore tested the effects of D167N without D49N. Although V3 resistance was restored, this mutant was still poorly V2-sensitive (Fig 7B, lane 12).

Considering the negative impact of the V169R mutation on JR-FL (Fig 4B), we wondered if essentially the reverse mutation, i.e., knocking out Q23’s basic residue by a R169I mutation might improve its expression. This was not the case, and sensitivity to V2 and FP NAbs was reduced or eliminated (Fig 7B, compare lanes 7 and 13).

Given Q23’s CH01 UCA sensitivity, we tested if it could also stimulate CH01 UCA expressed on the surface of B cells *ex vivo*. Total (CD19+ B220+) B cells from the spleens of naïve CH01 UCA ‘double knock in’ (dKI+) mice, i.e., expressing both CH01 heavy and light chain rearrangements [112] were effectively labeled by WITO SOSIP (S8A Fig). As expected, an anti-IgM Fab2 induced calcium flux. Q23 SOS D49N+N611A VLPs also stimulated *ex vivo* CH01 UCA dKI+ splenic B cells effectively and this result titrated (S8B Fig). GnT1- VLPs induced more robust stimulation, whereas bald VLPs did not stimulate cells (S8B Fig). Thus, Q23 VLPs may be highly effective at priming CH01-like specificities in a vaccine regimen, especially if they are produced in GnT1- cells.

### WITO

We next attempted to improve WITO, another well-expressed (Fig 3B), V2-sensitive (Fig 2) group 1 strain. Above, we saw that CH01 neutralization of WITO gp160ΔCT WT PV was sub-saturating (Fig 2). However, using the pQC-Fluc assay to improve WITO SOS PV infection (Fig 6), we found that like JR-FL SOS PV, WITO SOS PV was neutralized by CH01 with somewhat better saturation, and an IC50 was measurable (Figs 7D and S9A). We next checked the effects of removing the 3 V1 glycans at positions 133, 140 and 145 alone and together. Expression of N133A was lower, but N140A and N145A expressed similar to the parent (Figs 7C and S9B) and sensitivities to multiple V2 NAbs were slightly higher (Fig 7D, lanes 2–4). However, removing all 3 glycans together abolished CH01 sensitivity and also caused some V3 sensitivity (Figs S9A and 7D, lane 5).

An I169K mutation in strand C might improve V2 sensitivity. However, expression and infectivity were poor (Figs S9B and 7C, lane 6), as with the equivalent JR-FL mutant (Fig 4A). We attempted to improve apex folding via G300N+R305K mutations at the V3 loop base that may interact with Y173 and Y177 of the V2 loop [105]. To accelerate screening, we combined this double mutant with TL514-515GI (FPvar1) and N611A to knock in FP sensitivity. However, this mutant was overtly V3-sensitive and CH01-resistant (Figs S9A and 7D, lane 7)—essentially the reverse of the desired effect. The same mutant lacking the G300N+R305K (Fig 7D, lane 9) was not V3-sensitive, suggesting that G300N+R305K causes misfolding. TL514-515GI slightly improved VRC34 sensitivity (Fig 7D, lane 8), and, as expected, combining this with N611A led to a dramatic further increase in VRC34 neutralization (Fig 7D, lane 9).

We next tested the effects of removing the unusual N49 and N674 glycans (Figs 1 and S1). Both mutations reduced Env expression (Figs S9B and 7C, lanes 10 and 11). Analysis of 4,582 M-group Env sequences from the Los Alamos HIV Database reveals that the N49 glycan is ~10% conserved (S1 Fig) and more common in clade B, moderate in clade D, F1, G, AG and AE, but virtually absent elsewhere (S9C Fig). Above, we saw that knocking in the N49 glycan had no effect on JR-FL expression (Fig 4A) but improved Q23 expression slightly (Fig 7A). The N674 glycan is slightly more prevalent (13% conserved) and is present in 5 of our 17 strains (Figs 1 and S1). However, none of these other N674 glycan-containing strains were well-expressed, suggesting that the N674 glycan alone does not partition with high expression.

Finally, we combined the FP-immunofocusing mutant TL514-515GI+N611A with D167N to try to create a highly V2- and FP-sensitive combination mutant. This improved WITO sensitivity to multiple V2 NAbs, albeit with a moderate increase in V3 sensitivity (Figs S9A and 7D, lane 12), similar to the JR-FL D167N mutant (Figs S4A and 4, lane 35).

### T250

**Fig 8:**
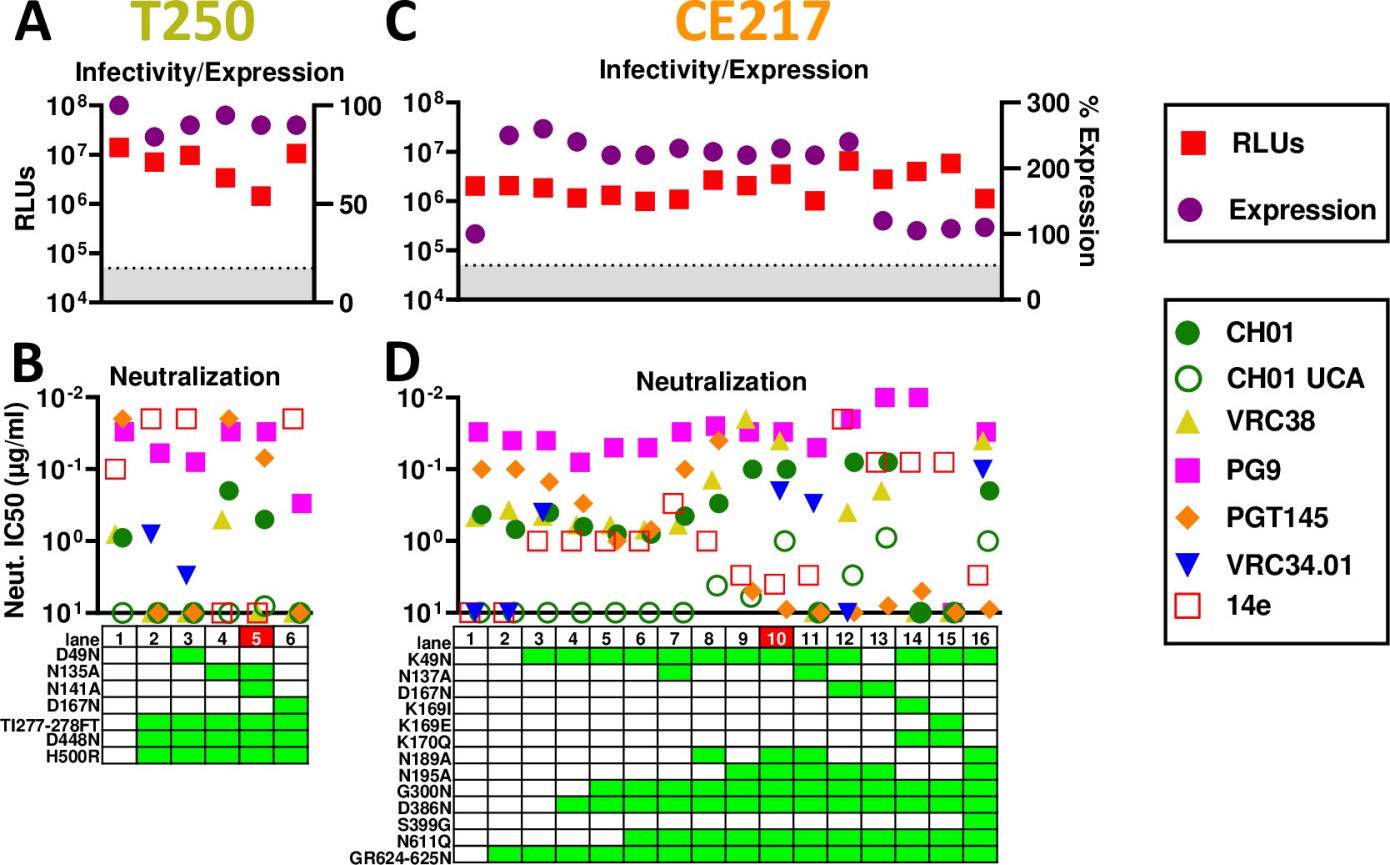
Effects of T250 and CE217 SOS gp160ΔCT mutations on trimer expression, infectivity and MAb sensitivity. Effect of mutations on A) T250 and C) CE217 gp160ΔCT SOS trimer infectivity (by pQC-Fluc and pNL-Luc assays, respectively) and expression (by SDS-PAGE-Western blot). B) and D) MAb sensitivities of mutants.

T250 is another well-expressed group 1 strain. In the gp160ΔCT SOS parent, we filled glycan holes at positions 276 and 448 and optimized the gp120/gp41 processing site. These repairs slightly reduced expression and infectivity (Fig 8A, lanes 1 and 2). Both were PG9-, CH01- and weakly CH01 UCA-sensitive (S10A and S10B Fig)—the latter being a rare feature so far shared only with Q23. However, the repaired mutant was PGT145-resistant (Figs S10D and 8B, lane 2). Poorly saturating 14e neutralization suggested partially open trimers, so it was not surprising that the D49N mutant led to overt V3 sensitivity (Figs S10C and 8B, lane 3). Removing one or both V1 glycans modestly improved CH01 sensitivity and reduced V3 sensitivity (Figs 8B, lanes 4 and 5 and S10A and S10C). In contrast, D167N lost V2 sensitivity, similar to the D49N mutant, and became overtly V3-sensitive (S10C Fig).

### CE217

CE217 is a highly V2-sensitive, but modestly expressed group 1 strain (Fig 3B–3D). Sequence alignments reveal an unusual insertion at position 625 (S1 Fig) that could adversely impact gp41 helix folding. We repaired this via a GR624-625N mutation, making it more consistent with other strains (S1 Fig). This dramatically improved expression (S11A Fig), but did not improve infectivity (Fig 8C, lanes 1 and 2). K49N knocked in VRC34 sensitivity but did not impact expression (Figs S11A and 8C, lanes 2 and 3). A D386N mutation to fill in a glycan hole led to slightly decreased PGT145 and PG9 sensitivity (Fig 8D, lane 4). Overlaying G300N [105] further reduced PGT145 sensitivity (Fig 8D, lane 5), but had little effect on CH01 and its UCA (S11B and S11C Fig). N611Q knocked in VRC34 sensitivity, as expected (Fig 8D, lane 10). Removing V1 and V2 clashing glycans improved V2 sensitivity overall (Fig 8D, lanes 7–11). The N195A mutant was highly sensitive to PG9, CH01 and VRC38. However, PGT145 sensitivity was lost, suggesting that V2 NAb sensitivities are differentially regulated by N195 glycan removal (Figs 8D, compare lanes 6 and 9 and S11B and S11E). N189A and N195A mutants were also modestly susceptible to CH01 UCA (Figs S11C and 8D, lanes 8 and 9). Removing both N189A and N195 glycans, increased CH01 UCA sensitivity further (Fig 8D, lane 10). Combination V1 glycan knockout mutant N137A+N189A+N195A eliminated CH01 and VRC38 sensitivities (Fig 8D, lane 11). Overlaying D167N on the N195A mutant improved PG9, but not CH01 sensitivity (Fig 8D, compare lanes 9 and 12). VRC38 and VRC34 sensitivities were reduced and lost, respectively and 14e sensitivity became overt (S11D Fig). Reverting K49N slightly reduced V3 sensitivity and modestly restored V2 NAb sensitivity, but reduced expression (Fig 8D, lane 13).

K169I, K169E and K170Q mutants were made to try to reduce V2 sensitivity for late boosting. However, these mutants completely eliminated V2 sensitivity, reduced expression and were overtly V3-sensitive (Fig 8C and 8D, lanes 14 and 15). Finally, an S399G mutation to eliminate the 397 sequon that overlaps with a more conserved sequon at N398 (S1 Fig) did not alter NAb sensitivities but reduced expression and infectivity (Fig 8D, lanes 10 and 16).

### AC10

**Fig 9:**
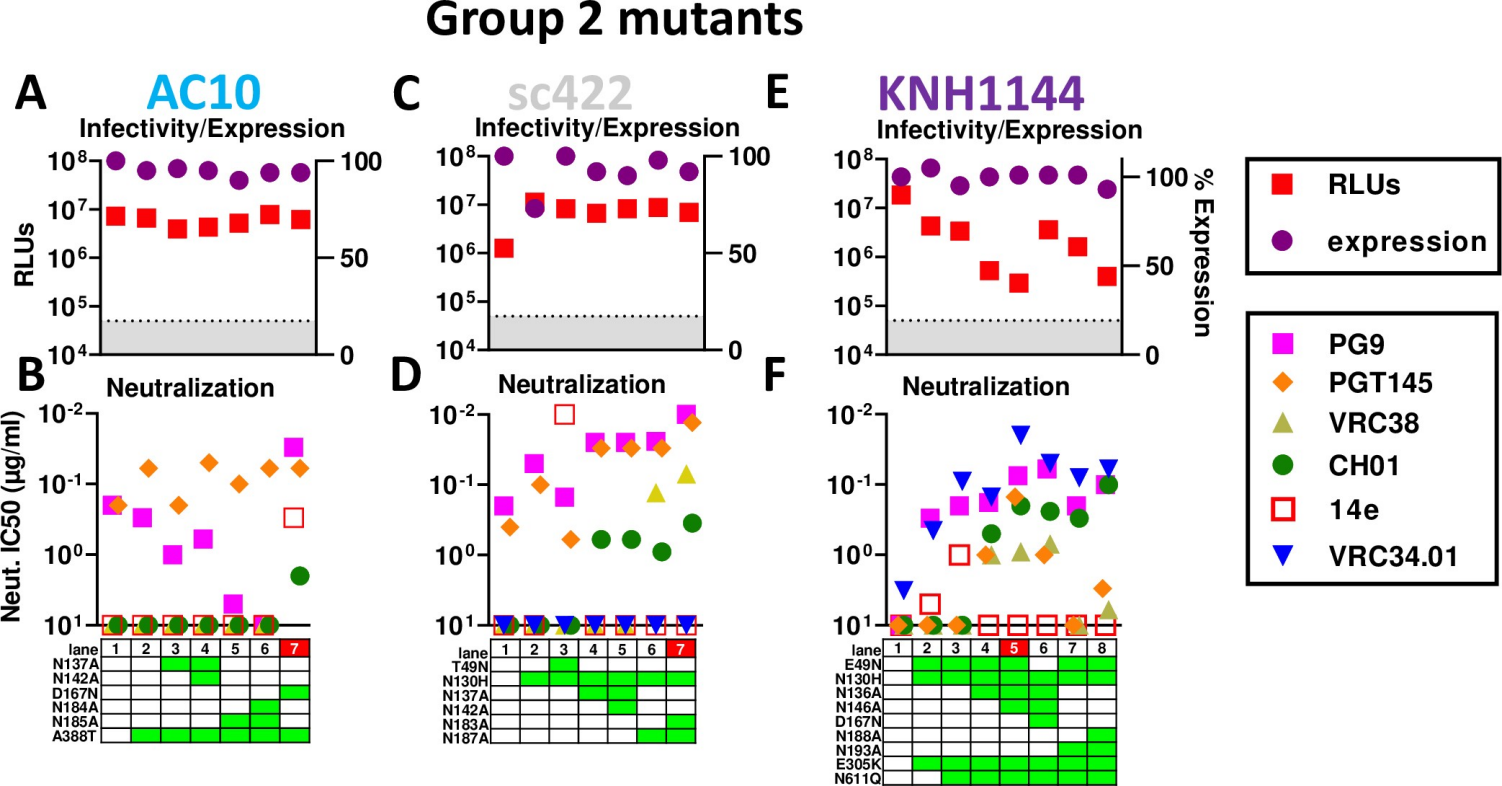
Effects of group 2 strain AC10, sc422 and KNH1144 SOS gp160ΔCT mutations on trimer expression, infectivity and MAb sensitivity. Effect of mutants on A) AC10, C) sc422 and E) KNH1144 gp160ΔCT SOS trimer infectivity (by pQC-Fluc assay) and expression (by SDS-PAGE-Western blot). B), D) and F) MAb sensitivities of mutants.

Given our success with JR-FL (Fig 4), we took a similar strategy with other group 2 strains. The AC10 parent is already PG9- and PGT145-sensitive and lacks a clashing N130 glycan (Fig 1). The SOS parent and A388T glycan hole-filled mutants both retained PG9- and CH01- sensitivity and were V3-resistant. Our data above suggest that "outermost" glycans (i.e., those closer to the V1V2 base) may be more prone to V2 clashes (Figs 4, 7 and 8), so we removed these from AC10 first, alone and together with inner glycans. Mutants N137A and N137A+N142A led to modest changes in PGT145 and PG9 sensitivity but remained CH01- and VRC38-resistant (Figs 9B and S12A). V2 glycan mutants N185A and N184A+N185A (Figs 1 and S1), led to PG9 resistance, but remained sensitive to PGT145 and resistant to 14e, CH01 and VRC38 (Figs 9B and S12A). Finally, D167N mutant increased PG9 and CH01 sensitivity, but was also partially V3-sensitive. PGT145 NAb saturation was also reduced.

### sc422

sc422 is the best expressed clone in our panel (Fig 3B–3D). It is also PGT145-sensitive (Figs 1 and 2A). Although PG9 did not neutralize the WT parent (Figs 1 and 2), the SOS mutant was sensitive (Fig 9D). An N130H mutant further increased PG9 and PGT145 sensitivity, but CH01 and VRC38 resistance was intact (Figs S12B and 9D, lane 2). T49N mutation led to overt V3 sensitivity and reduced PG9 and PGT145 sensitivities (Figs S12B and 9D, lane 3). Removal of V1 and V2 clashing glycans significantly increased sensitivity to multiple V2 NAbs (Figs S12B and 9D, lanes 4–7). Removal of the V2 glycans N183 and N187 knocked in VRC38 susceptibility. N183A+N187A was the most sensitive mutant to multiple V2 NAbs and retained complete V3-resistance.

### KNH1144

KNH144 is a poorly V2-sensitive group 2 strain. Accordingly, we made several initial mutants in combination: E49N to try to maximize expression, N130H to eliminate a V2 clashing glycan and E305K to try to improve V1V2 packing [105]. These changes knocked in PG9- and VRC34-sensitivity and sub-saturating 14e-sensitivity (Fig 9F, lane 2). CH01 sensitivity was also detected (S12C Fig) but did not reach an IC50 (Fig 9F). PG9 sensitivity may be due to N130H mutation and/or E305K. VRC34 and 14e sensitivities were likely a result of E49N. N611Q improved VRC34 sensitivity, as expected (Fig 9F, lane 3). Finally, we removed potentially clashing V1 and V2 glycans, starting with those closest to the base of each loop (i.e., N136A and N193A), then double mutants. Both single mutants (N136A and N193A) resulted in detectable CH01 IC50s, albeit sub-saturating (Fig 9F, lanes 4 and 7). Double mutants (N136A+N146A and N188A+N193A) both improved CH01 IC50s slightly as well as its saturation (Figs S12C and 9F, lanes 5 and 8). Only the V1 glycan knockouts resulted in moderate VRC38 and PGT145 sensitivity (Fig 9F, lanes 4 and 5). To increase the sensitivity of the mutant in Fig 9F, lane 5, we overlaid a D167N mutation and reverted the N49 glycan. This had surprisingly modest impact on V2 sensitivity, except for a loss of PGT145 sensitivity (Fig 9F, lane 6). VRC34 neutralization was also reduced, suggesting that the E49N and N611Q mutations both assist VRC34 sensitivity in this case (Fig 9F, lane 5 and 6), unlike in JR-FL (Fig 4).

### Optimizing other strains and engineering approaches

Our attempts to optimize trimers from strains c1080 and 6101 are described in S4 Text. Other novel engineering approaches are described in S5 Text.

### A quarter of particles from transfected cells carry surface Env trimers

We previously found that codon-optimized MuLV Gag drives higher yields of Env trimer in transfection supernatants, versus using pNL-LucR-E- [24]. Electron microscopy showed that some particles bear surface spikes [77,113], although "bald" particles with no spikes are common. We hypothesized that MuLV Gag-mediated budding might outpace membrane Env expression, decreasing the proportion of "Env+" VLPs and/or lowering spike density.

**Fig 10:**
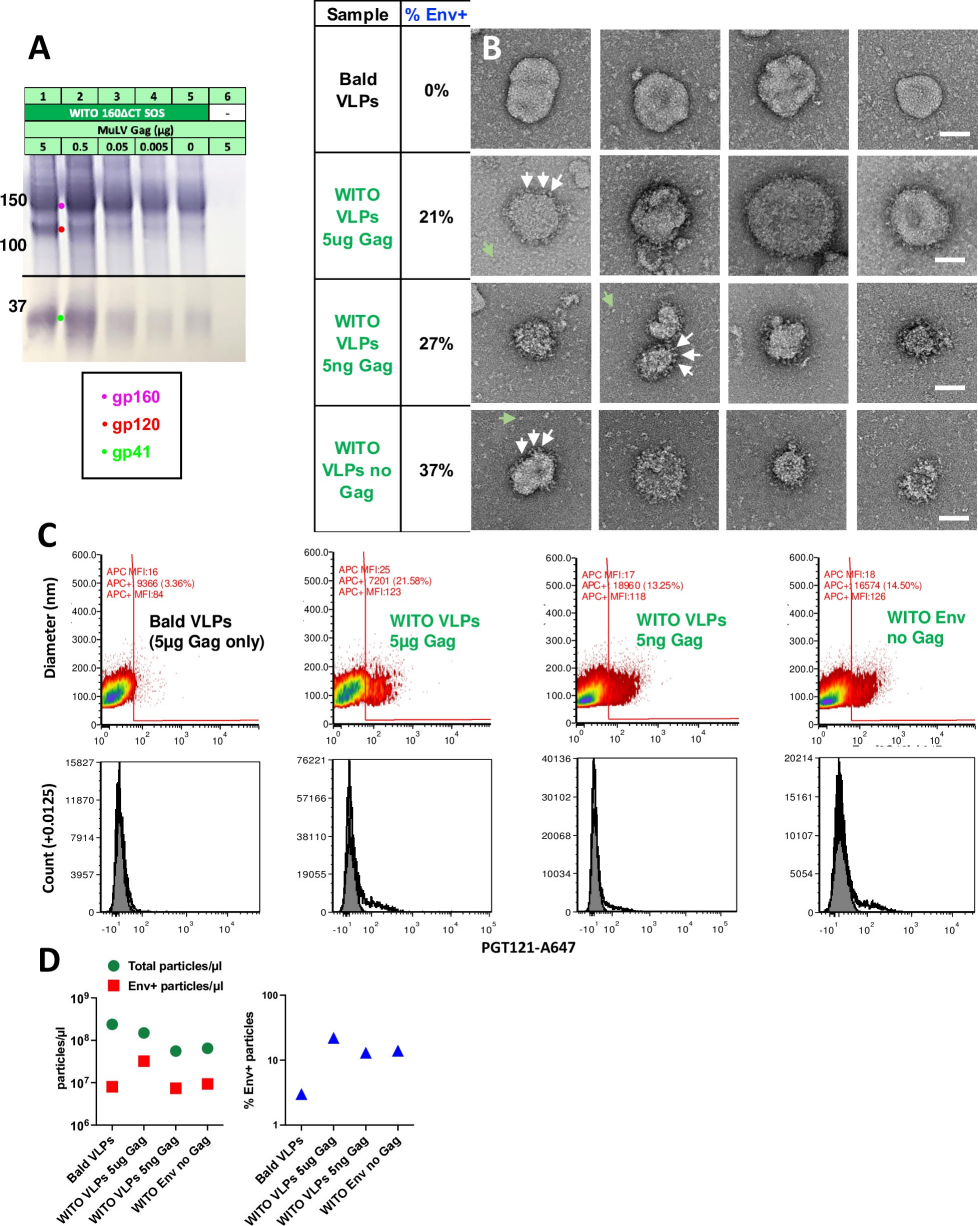
A quarter of particles from transfections using Env plasmid carry surface Env. 293T cells were transfected with WITO SOS gp160ΔCT and/or MuLV Gag, as indicated in part A). Supernatants were precleared, filtered and 1,000-fold concentrated. Samples were probed by A) SDS-PAGE-Western blot, B) negative stain EM (scale bars are 50nm, white arrows point to candidate Env trimers and green arrows point to possible dissociated spikes, and C) Single vesicle flow cytometry. Upper panels show particle diameters and fluorescence intensities after staining with Alexa-647-labeled PGT121. In the lower panel, we show total particle counts versus Alexa-647 fluorescence. D) Total particle and Env+ particle counts per μl of samples are indicated (left) and % Env+ particles as a proportion of total particles (right). Raw vFC data files and data analysis layouts have been deposited in Flowrepository (flowrepository.org; see S6 Text).

To investigate the Gag-dependency of membrane spike expression, we co-transfected a fixed amount of WITO SOS gp160ΔCT with 10-fold decreasing Gag doses. We also transfected Env only and Gag only controls. Supernatants were filtered and 1000x concentrated. As expected, higher doses of MuLV Gag generally resulted in more particulate Env (Fig 10A). However, Env was detected even when 1,000-fold less Gag or no Gag was co-transfected (Fig 10A, compare lanes 1, 4 and 5). Env was not detected in the Gag only sample (Fig 10A, lane 6).

Gag only "bald" VLPs (Fig 10B, top row) provided a reference to help identify Env spikes on other samples by negative stain EM. WITO Env-transfected samples (Fig 10B, rows 2–4) revealed particles with surface structures that we infer to be Env spikes. These putative spikes do not adopt clear propellor-like structures, perhaps because, unlike rigidified soluble trimers, native, membrane trimers are flexible and sample different conformations that are harder to resolve.

Bald particles were also prevalent in all samples. Counting Env+ and Env- particles in each sample revealed that approximately a quarter to a third were Env+. There was a trend towards a greater proportion of Env+ particles in samples made using little or no Gag (Fig 10B). Thus, our concern that efficient Gag-induced particle production might lead to an overwhelming proportion of bald VLPs appears to be unfounded. Debris, indicated by green arrows in Fig 10B, rows 2–4, may be spikes that dissociate from VLPs that collapse during processing. Finally, particle sizes varied. Many particles were ~100nm diameter, but some were much larger, up to ~300nm. It is not clear if the latter are Gag-driven or Gag-independent extracellular vesicles.

We next analyzed the same VLPs by single vesicle flow cytometry (vFC, Cellarcus Biosciences), which uses a fluorogenic membrane probe to detect and size vesicles, and fluorescent MAbs to measure vesicle surface cargo by immunofluorescence [114,115]. This revealed a modest, but consistent proportion of Env+ particles bound PGT121 (13.25–21.58%) in samples transfected with WITO Env (Fig 10C, columns 2–4 and D, right plot). In contrast, VLPs from cells expressing Gag but no Env ("bald VLPs") showed only background PGT121 binding (Fig 10C, first column). Transfecting with Gag alone yielded the highest particle count of 2.4x10^8^/μl of sample (Fig 10D). Co-transfecting Env reduced the particle count by ~60% to 1.5 x 10^8^/μl (Fig 10D, left). Transfecting with only 5ng or no Gag reduced particle counts a further 2-fold (Fig 10D, left). We can infer that ~25% of particles in these samples form spontaneously and are Gag-independent, putting a new perspective on the increased Env output when transfecting with high amounts of Gag versus no Gag (Fig 10A, compare lanes 1 and 5). Essentially, transfecting with a high dose Gag increases particle numbers by only ~4-fold with a concomitant increase in Env, as particle production is already high even with no Gag transfection. Overall, this data agrees quite well with the EM data in that particles are 13–37% Env+ and the proportion of Env+ particles does not change much with Gag co-transfection, which is perhaps not surprising, if Gag only raises above spontaneous particle production levels by about 4-fold.

The single vesicle flow cytometry analysis revealed other pertinent information. The x axis in Fig 10C (PGT121 binding fluorescence) is calibrated in MAb units/vesicle to estimate spike density. Bald particles (Gag only) have a median autofluorescence of ~16 MAb molecules (Fig 10C, left column), whereas Env+ samples bound ~125 MAbs/particle. PGT121 may bind up to 3 MAbs/trimer [116]. Thus, extrapolating the number of MAbs/particle to number of spikes/particle involves some uncertainty. Subtracting autofluorescence lowers our estimate to ~109 PGT121 molecules/particle. If 3 PGT121 molecules can bind per trimer at saturation, then spike density is at ~30–40 trimers/particle. This aligns quite well with our previous estimate of 27 spikes/particle for JR-FL VLPs [77]. There was no clear difference in the extent of MAb binding between samples, suggesting that spike density does not vary, consistent with the finding above that the proportion of Env+ particles also does not change markedly between samples (Fig 10C and 10D). Finally, this method revealed that most particles were 70-180nm in diameter, centered around 120nm, although some were much larger, up to ~300nm, again in agreement with EM data (Fig 10B and 10C).

## Discussion

**Fig 11:**
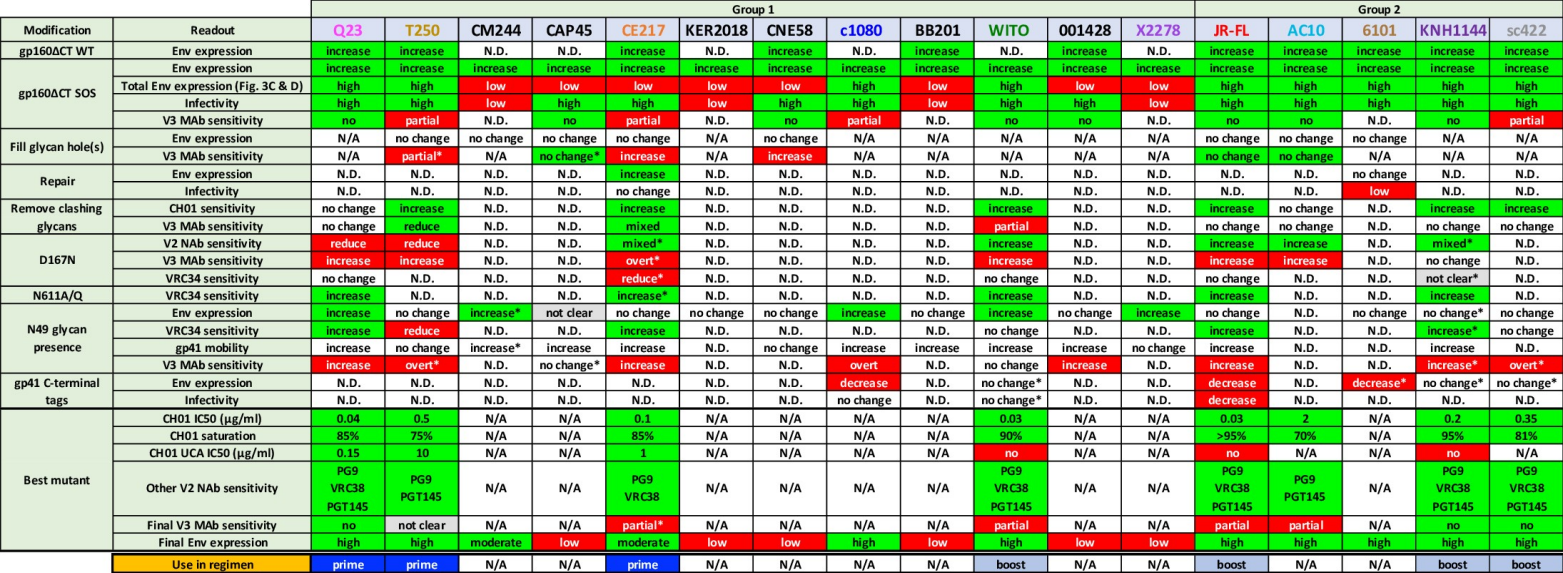
Summary of efforts to develop immunofocusing trimers. The 17 strains are partitioned into groups 1 and 2. The effects of modifications on expression, infectivity and sensitivity to V2 and/or V3 MAbs are indicated. Desired features are depicted in green boxes. Unwanted features are depicted in red boxes. Features of selected clones are shown in the lower rows along with their V3-sensitivities and potential use in vaccine regimens. Asterisks indicate modifications that were made in combination with others, so any effects observed cannot be unambiguously linked to any one modification.

Here, we identified 7 V2- and FP-sensitive trimers (Fig 11). Modified Q23, T250 and CE217 trimers could be used as priming immunogens, with modified WITO, JR-FL, sc422 and KNH1144 trimers as boosts. Our efforts show that gp41 tail truncation and SOS mutation consistently improve expression. The optimization process can be accelerated by initially correcting all "errors of nature", by filling glycan holes, and resolving insertions, deletions and overlapping sequons. Although these and other repair strategies occasionally improved expression or infectivity (Fig 11), we did not discover a "magic formula" for engineering desirable trimers. Depending on strain, membrane trimers can exist in a variety of ground states. It may be that trimers with particular phenotypes, e.g., the macrophage-tropic JR-FL strain, can benefit from particular engineering strategies. Certainly, the JR-FL strain is a good choice for membrane trimers as it is robustly V3-resistant, meaning that it endures various engineering strategies without becoming overtly V3-sensitive, unlike some of our other strains, such as c1080. Some trimers were partially V3-sensitive but also V2-sensitive, as described previously [109]. In some cases, loosening the V2 apex was beneficial. For example, SOS improved CH01 saturation of JR-FL and WITO, but in other strains, SOS mutants were *less* CH01-sensitive than the WT parent (Q23, T250 and CE217). Notably, PGT145 sensitivity of gp160ΔCT SOS trimers was somewhat reduced in all cases, as the tightly folded V2 apex targeted by this NAb is slightly perturbed by both of these mutations [117].

Most repair strategies that are helpful for SOSIP [23,40,90] proved to be unhelpful for membrane trimers. This difference is underlined by the distinct limiting factors of membrane trimers (i.e., expression) versus SOSIPs (i.e., "closed" trimers), which is reflected in the fact that different strains make better prototypes e.g., JR-FL and BG505, respectively. The fact that codon optimization, modified signal peptides [118], different expression plasmids and lentiviral vectors were unhelpful—all suggest that the expression bottleneck is not at the level of transcription, but relates to protein folding. Various domain swaps were also unhelpful, perhaps because, unlike rigidified SOSIP, native trimers are flexible, increasing the possibility that engraftments can perturb quaternary folding. One strategy we did not attempt was to screen for highly expressed clonal relatives of poorly expressed group 1 strains [110].

Attempts to "close" partially open trimers face a complex and nuanced challenge to identify residues that render the trimer open in the first place, which may be strain-dependent. For example, in the case of T250, the V1 glycans limit V2 apex folding and NAb sensitivity, but this had less effect in other contexts. Approximately 10% of our mutants were overtly V3-sensitive, with IC90s <0.01μg/ml, suggesting a complete loss of V-loop apex interactions. In many cases, this phenotype was coupled with low expression and infectivity, consistent with misfolding. Since these mutants are common, they have been well-documented by many groups [3,35,36,105,119–122]. While it could be inferred that these mutants suggest a role of the mutated amino acid(s) in actively maintaining quaternary trimer interactions, this is usually not the case. Instead, the mutants may simply be incompatible with quaternary structure.

Unlike soluble Envs, sequon skipping was limited in membrane trimers [68,79,80,84,123]. Also, unlike soluble Envs, attempts at sequon optimization were not helpful, as they disrupted trimer conformation (partial V3 sensitivity, increased skipping and/or glycan maturation changes). Again, this may be due to the flexibility of membrane trimers, versus rigid SOSIP, such that mutations can have more dramatic consequences for membrane trimers [13,80,124–126]. In general, membrane trimer mutants are only successful when they introduce a more conserved alternative residue at a given position. The varied effects of glycan toggling on proximal and distal glycans provides reason for caution. For example, a glycan hole that increases V2 apex sensitivity may only be effective if it does not open up other unwanted glycan holes. On the other hand, if "off-target" holes differ between successive vaccine shots, this could limit the problem.

V1V2 base glycans had the biggest impact on V2 NAb sensitivity. The greater effect of N135 on JR-FL compared to N138 or N141 is consistent with its closer physical proximity to the V1V2 apex (Fig 5A). Furthermore, the N130 glycan is absent in V2-sensitive group 1 strains and its removal from group 2 strains also improved their sensitivities. However, some V1V2 glycans can affect expression. For JR-FL, knocking in the N197 glycan did not appreciably impact either expression or V2 sensitivity, although N197 toggling can impact the V2 sensitivity of other strains [127].

Given the increased activity of CH01 on GnT1- PVs, it was no surprise that CH01 selectively bound to small high mannose glycans at N135 to avoid clashes. This selective glycovariant binding has been reported previously [84]. What we did not expect is for glycans at other sites to also be far less mature. The idea that CH01 bound an early "high mannose" trimer glycoform can be ruled out, as not all sequons were affected. For example, the N616 glycan was *more* mature in CH01 complexes. It could be that glycan maturation at different sites is co-dependent via a glycan network. If so, how can we reconcile the rarity or total absence of glycoforms at several sites in the corresponding uncomplexed parent? We assume that all glycoforms are equally infectious, but this may not be the case. Indeed, a significant fraction of JR-FL trimers remains uncleaved, while our glycopeptide analysis provides data on the *total* Env of VLPs, regardless of processing. Further analysis of MAb-complexed trimers may provide insights.

V2 bNAbs typically bind one-per-spike [63,95]. C strand charge is at a premium during V2 NAb ontogeny. Therefore, we increased strand C’s positive charge, while being mindful of acceptable substitutions in sequence alignments. Conserved K/R residues at positions 166, 168, 169 and 171 are important for V2 NAb binding. Of these, 166 and 171 were present in all but strain 6101. K/R168 was present in all but JR-FL, where E168K was an effective knock in mutant. D167N, found in V2 NAb-initiating clones consistently improved V2 sensitivity, albeit with a concomitant increase in V3 sensitivity. However, mutating position 169 to a basic residue in JR-FL and WITO was not tolerated, although it was helpful in other settings, suggesting a context-dependent effect [95].

A new, state-of-the-art method [128] revealed that only ~25% of particles in VLP preparations are Env+, the rest being bald [114,115]. Some particles may derive from FBS that carries ubiquitous vesicles. After transfection, cells are washed and replaced with 1% FBS medium, so FBS-derived vesicles are likely to co-purify with VLPs. This is supported by the observation that co-transfecting high doses of Gag plasmid with Env plasmid produces a higher total particle count and a higher total number and fraction of Env+ particles compared to Env plasmid transfection alone (Fig 10D, lanes 2 and 4). This is consistent with the production of Env+ particles amid a *constant* background of FBS vesicles. However, since Gag transfection increases particle production by only 2-fold compared to Env only transfection, hard interpretations are difficult. Additional sources of bald particles might be spontaneous budding of endogenous cellular Gag or Env-induced ER stress, consistent with the presence of uncleaved gp160 that bypasses furin [129].

One concern is that the high proportion of vesicles in VLP preparations, if formulated with adjuvant, might trigger auto-immune antibody and T cell responses. To date, we have immunized well-over a hundred animals with no documented side effects. Nevertheless, to address this, it may be possible to purify Env+ particles by immunocapture. If Env- particles are higher in some other marker (e.g., CD81) because they bud differently or originate from FBS, then they could be fractionated. Alternatively, our vaccine concepts could be adapted to an mRNA-LNP platform, thereby side stepping this concern. The use of HIV Gag to drive budding could provide additional T cell help.

We plan to use CH01 UCA-sensitive and FP-sensitive mutants to prime, followed by boosts with a variety of strains and/or mutants to increase stringency and promote binding to anchor residues and to tolerate sequence variation and clashing glycans. Thus, strand C could become gradually more neutral, mimicking waves of diversity in V2 NAb ontogeny in natural infection [25,37,70,110]. In a vaccine setting, strain variation between shots could be helpful, provided that bNAbs stays on track. Overall, the diverse panel of membrane trimers described here will allow us to test a variety of vaccine concepts for immunofocusing V2 and FP NAbs.

## Materials and methods

### Plasmids

#### i) HIV-1 Env plasmids

Abbreviated Env strain names are given first, with full names and GenBank references in parentheses: Q23 (Q23.17; AF004885.1), WITO (WITO.33, AY835451.1), c1080 (c1080.c3, JN944660.1), CM244 (CM244.ec1, JQ715397.1), T250 (also known as CRF250, T250.4, EU513189.1), 001428 (001428–2.42, EF117266.1), CE217 (CE703010217.B6-1, DQ056973.1), BB201 (BB201.B42, DQ187171.1), KER2018 (KER2018.11, AY736810.1), CNE58 (CNE58, HM215421.1), CAP45 (CAP45.G3, DQ435682.1), X2278 (X2278.c2.B6, FJ817366.1), JR-FL (JR-FL, AY669728.1), AC10 (AC10.29, AY835446.1), KNH1144 (KNH1144ec1, AF457066.1), sc422 (SC422661.8, AY835441.1), 6101 (6101, AY669708.1).

Full-length Env clones of the above strains, commonly used to make PVs for neutralization assays, were obtained from the NIH AIDS Reagent Repository, the Vaccine Research Center and The Scripps Research Institute. Most were in expression plasmids pCI, pCDNA3.1, pCAGGS or pVRC8400. However, the modestly expressing plasmid pCR3.1 was used for Q23.17 and BB201.

#### ii) Gag and Rev plasmids

A plasmid expressing murine leukemia virus (MuLV) Gag [24]. When Env plasmids use native codons, we co-transfected pMV-Rev 0932 that expresses codon optimized HIV-1 Rev to maximize Env expression.

#### iii) Glycosyltransferase plasmids

Glycosyltransferase plasmids pEE6.4_B4GalT1 (expressing β-1,4 galactosyltransferase) and pEE14.4_ST6Gal1 (expressing β-galactoside α-2,6-sialyltransferase) were co-transfected at a ratio of 1% and 2.5% total plasmid DNA, respectively.

**iv) MAb plasmids.** MAb plasmids were obtained from their producers or the NIH AIDS Reagent Repository. These included CH01/CH04, VRC38.01, PG9, PG16, and PGT145 directed to the V2 apex epitope; 39F and 14e directed to the V3 loop of gp120; VRC34.01 directed to the gp120-gp41 interface; and fusion peptide MAb vFP16.02 [8]. UCAs of MAbs CH01/CH04 and VRC38.01 were described previously [14].

### VLP and gp120 monomer production

For VLP production, Env plasmids were co-transfected in Human Embryonic Kidney 293T or GnT1- 293S cells using polyethyleneimine (PEI Max, Polysciences, Inc.), along with the MuLV Gag plasmid [24] and pMV-Rev 0932, as needed. 48h later, supernatants were collected, precleared, filtered, and pelleted at 50,000g in a Sorvall SS34 rotor. Pellets were washed in 1ml of PBS, recentrifuged in a microcentrifuge at 15,000 rpm, and resuspended at 1,000x the original concentration in PBS. JR-FL gp120 monomer was produced and purified as described previously [77].

### Neutralization assays

Assays were repeated at least twice to ensure consistency.

#### i) NL-Luc assay

Pseudoviruses (PV) were produced by co-transfecting 293T or 293S GnT1- cells with pNL4-3.Luc.R-E and an Env plasmid using PEI Max. Briefly, PV was incubated with graded dilutions of MAbs for 1 hour at 37°C, then added to CF2Th.CD4.CCR5 cells, plates were spinoculated, and incubated at 37°C [14]. For wild-type (WT) PV, plates were incubated for 3 days, after which luciferase was measured. For SOS PV, following a 2-hour incubation, 5mM DTT was added for 15 minutes to activate infection. The MAb/virus mixture was replaced by fresh media, cultured for 3 days, and luciferase activity measured.

#### ii) pQC-Fluc assay

PV were produced by co-transfecting Env plasmids with pMLV GagPol and pQC-Fluc-dIRES (abbreviated as pQC-Fluc) [111]. The resulting PV were used in neutralization assays with CF2Th.CD4.CCR5, as above.

#### iii) Post-CD4 assay

PV were mixed with sCD4 with or without V3 MAbs 14e or 39F. This mixture was then added to CF2.CCR5 cells, as described previously [103].

### Blue Native PAGE-Western blot

VLPs were solubilized in 0.12% Triton X-100 in 1mM EDTA. An equal volume of 2x sample buffer (100mM morpholinepropanesulfonic acid (MOPS), 100mM Tris-HCl, pH 7.7, 40% glycerol, and 0.1% Coomassie blue) was added. Samples were spun to remove any debris and loaded onto a 4–12% Bis-Tris NuPAGE gel (Thermo Fisher) and separated for 3 hours at 4C at 100V. Proteins were then transferred to polyvinylidene difluoride (PVDF) membrane, de-stained, and blocked in 4% non-fat milk in PBST. Membranes were probed with a cocktail of MAbs 39F, 2F5, b12, 4E10, 14e, and PGT121, followed by alkaline phosphatase labeled anti-human Fc conjugate (Accurate Chemicals) and were developed using SigmaFast BCIP/NBT (Sigma).

### SDS-PAGE-Western blot

VLPs were denatured by heating with 2-mercaptoethanol for 10 minutes at 90°C, then mixed with Laemmli buffer, then loaded onto 4–12% Bis-Tris NuPAGE gel (Invitrogen). To examine cleavage of oligomannose and hybrid glycans, endonuclease H (Endo H, New England Biolabs) was added to the samples after reduction and denaturation, followed by incubation for 1h at 37°C. Proteins were transferred onto a PVDF membrane, de-stained, and blocked in 4% non-fat milk in PBST. Blots were probed as for BN-PAGE blots. Env band densities were quantified using Image Studio Lite (LI-COR).

### Reduction, alkylation and digestion of Env

JR-FL gp120 monomer was denatured for 1h in 50 mM Tris/HCl, pH 8.0 containing 6M urea and 5 mM dithiothreitol (DTT). Next, Env proteins were reduced and alkylated with 20mM iodoacetamide (IAA) for 1h in the dark, followed by a 1h incubation with 20mM DTT to eliminate residual IAA. Alkylated Env proteins were buffer exchanged into 50mM Tris/HCl, pH 8.0 using Vivaspin columns (3 kDa). Aliquots were digested separately overnight using trypsin and chymotrypsin (Mass Spectrometry Grade, Promega). The next day, peptides were dried and extracted using C18 Zip-tip (Merck Millipore).

JR-FL VLPs were processed in the same way, except that were initially buffer exchanged into 50mM Tris HCl 0.1% Triton X-100 (w/w) to disperse lipids. To identify the glycome of the trimers that complexed with MAb CH01, VLPs were mixed with excess CH01 and incubated for 1h at 37°C. Screw cap spin columns were incubated with protein A–agarose for 10 minutes to allow for spin column resin equilibration before washing with gentle Ag-Ab binding buffer (Thermo Fisher Scientific). VLP-CH01 complexes were then applied to the spin columns and left to incubate for 30 minutes. Columns were washed twice with gentle Ag-Ab binding buffer prior to elution in 100–200 μL gentle Ag-Ab elution buffer (Thermo Fisher Scientific). Eluted VLP-CH01 mixtures were then buffer exchanged into 100μL 50mM Tris/HCl pH 8.0 for subsequent reduction and alkylation.

### Liquid chromatography-mass spectrometry (LC-MS) glycopeptide analysis

Peptides were dried again, re-suspended in 0.1% formic acid and analyzed by nanoLC-ESI MS with an Ultimate 3000 HPLC (Thermo Fisher Scientific) system coupled to an Orbitrap Eclipse mass spectrometer (Thermo Fisher Scientific) using stepped higher energy collision-induced dissociation (HCD) fragmentation. Peptides were separated using an EasySpray PepMap RSLC C18 column (75 μm × 75 cm). A trapping column (PepMap 100 C18 3μM 75μM x 2cm) was used in line with the LC prior to separation with the analytical column. LC conditions were as follows: 280 minute linear gradient consisting of 4–32% acetonitrile in 0.1% formic acid over 260 minutes, followed by 20 minutes of alternating 76% acetonitrile in 0.1% formic acid and 4% acetonitrile in 0.1% formic acid to ensure all the sample elutes from the column. The flow rate was set to 300nL/min. The spray voltage was set to 2.7 kV and the temperature of the heated capillary was set to 40°C. The ion transfer tube temperature was set to 275°C. The scan range was 375−1500 m/z. Stepped HCD collision energy was set to 15%, 25% and 45% and the MS2 for each energy was combined. Precursor and fragment detection were performed with an Orbitrap at a resolution MS1 = 120,000, MS2 = 30,000. The AGC target for MS1 was set to standard and injection time set to auto which involves the system setting the two parameters to maximize sensitivity while maintaining cycle time.

### Site-specific glycan classification

Glycopeptide fragmentation data were extracted from the raw file using Byos (Version 3.5; Protein Metrics Inc.). Glycopeptides were evaluated in reference to UniProtKB Q6BC19 (ectodomain of JR-FL gp160ΔCT). All samples carry additional mutations SOS (A501C, T605C) and E168K and N189A, along with other mutations, as denoted in the.txt files found in the MassIVE database (MSV000088108). Data were evaluated manually for each glycopeptide. A peptide was scored as true-positive when the correct b and y fragment ions were observed, along with oxonium ions corresponding to the glycan identified. The MS data was searched using the Protein Metrics “N-glycan 309 mammalian no sodium” library with sulfated glycans added manually. All charge states for a single glycopeptide were summed. The precursor mass tolerance was set at 4 ppm and 10 ppm for fragments. A 1% false discovery rate (FDR) was applied. Glycans were categorized according to the composition detected.

HexNAc(2)Hex(10+) was defined as M9Glc, HexNAc(2)Hex(9−3) was classified as M9 to M3. Any of these structures containing a fucose were categorized as FM (fucosylated mannose). HexNAc(3)Hex(5−6)X was classified as Hybrid with HexNAc(3)Hex(5–6)Fuc(1)X classified as Fhybrid. Complex glycans were classified according to the number of HexNAc subunits and the presence or absence of fucosylation. As this fragmentation method does not provide linkage information, compositional isomers are grouped, so, for example, a triantennary glycan contains HexNAc(5) but so does a biantennary glycans with a bisect. Core glycans refer to truncated structures smaller than M3. M9Glc- M4 were classified as oligomannose-type glycans. Glycans containing at least one sialic acid were categorized as NeuAc and at least one fucose residue in the “fucose” category.

Glycans were categorized into I.D.s ranging from 1 (M9Glc) to 19 (HexNAc(6+)(F)(x)). These values were multiplied by the percentage of the corresponding glycan divided by the total glycan percentage excluding unoccupied and core glycans to give a score that pertains to the most prevalent glycan at a given site. Arithmetic score changes were then calculated from the subtraction of these scores from one sample against others as specified.

### Construction of trimer model and cognate glycans

The model representation of the JR-FL SOS E168K+N189A trimer was constructed using SWISS-MODEL based on an existing structure of the 426c DS-SOSIP D3 trimer (pdb: 6MYY). Glycans were modelled on to this structure based on the most abundant glycoform identified from site-specific glycan analysis using WinCoot version 0.9.4.1 and PyMOL version 2.5.0. For sites which were not identified, a Man9GlcNAc2 glycan was modelled. Conditional color formatting was used to illustrate the predominant glycoforms of modeled glycans, as follows: green (high mannose), orange (hybrid) and magenta (complex).

### Phenotyping and calcium flux of CH01 UCA dKI-derived splenocytes

C57BL/6J WT or CH01UCA double KI (V_H_DJ_H_^+/+^ x VkJk^+/+^) splenocytes were phenotyped with 0.5μg/mL of anti-B220 BV650, anti-CD19 APC-R700 (Becton Dickinson) and WITO-SOSIP-BV421 HIV Env tetramers, washed then stained with LIVE/DEAD Fixable Near-IR Dead Cell Stain Kit (Thermo Fisher) for 30 min. To evaluate B-cell stimulation, splenocytes were stained with anti-B220 BV650 and anti-CD19 APC-R700 for 40 minutes. After washing with HBSS, pre-stained cells were loaded with Fluo-4 via by mixing with equal volumes of 2X Fluo-4 Direct™ loading solution (Fluo-4 Direct™ Calcium Assay Kit, Thermo Fisher). After a 30 min incubation at 37°C and then 30 mins at RT, cells were washed with HBSS and incubated with LIVE/DEAD Near-IR for 30 minutes. After another HBSS wash, cells were resuspended in calcium-containing HBSS and incubated at room temperature for 5 minutes before activation by anti-IgM F(ab′)2 (Southern Biotech) or VLPs. Fluo-4 MFI data for total B-cells (B220^**+**^CD19^**+**^) was acquired on a Beckman CytoFlex flow cytometer and analyzed using FloJo software.

### Negative-stain electron microscopy

A 4.8-μl drop of the sample was applied to a freshly glow-discharged carbon-coated copper grid for 10–15 s and removed using blotting paper. The grid was washed with several drops of buffer containing 10 mM HEPES, pH 7.0, and 150 mM NaCl, followed by negative staining with 0.7% uranyl formate. Staining quality and particle density were assessed using a Hitachi H-7650 transmission electron microscope. Representative images of VLPs were acquired with a Thermo Scientific Talos F200C transmission electron microscope operated at 200 kV and equipped with a Ceta CCD camera. The magnification was 57,000, corresponding to a pixel size of 0.25 nm.

### Flow cytometry analysis of particles

Particle concentration, size, Env+ fraction and spike density were determined by single vesicle flow cytometry [114,115], using a commercial kit (vFC Assay kit, Cellarcus Biosciences, La Jolla, CA) and flow cytometer (CytoFlexS, Beckman Coulter, Indianapolis, IN). Briefly, samples were stained with the fluorogenic membrane stain vFRed^TM^ and anti-Env MAb PGT121, labeled with AlexaFluor647 (Thermo Fisher) for 1h at RT and analyzed using membrane fluorescence to trigger detection. Data were analyzed using FCS Express (De Novo Software) and included calibration using a vesicle size and fluorescence intensity standards. The analysis included a pre-stain dilution series to determine the optimal initial sample dilution and multiple positive and negative controls, per guidelines of the International Society for Extracellular Vesicles (ISEV) [128]. A detailed description of vFC methods and controls can be found in S6 Text. A MIFlowCyt Item Checklist and MIFlowCyt-EV, as required by the guidelines are provided in S1 Table.

## Supporting information

S1 Fig Gp160ΔCT sequence alignment of candidate strains. Amino acids are numbered according to the prototype HXB2 sequence (Genbank: AAB50262.1). Conserved sequons (>80%) are highlighted in cyan; variable sequons (<80%) are highlighted in yellow. Glycan holes, in which >80% conserved glycans are missing, are shown in orange. Primary and secondary gp120-gp41 furin cleavage sites are shown in magenta. FP is highlighted in lavender. Overlapping sequons are boxed in black. CD5 signal peptide on certain strains is highlighted as green (AA1-31).
(TIF)

S2 Fig Effect of V2’ glycans and SOS mutation on JR-FL pseudovirus sensitivity to V2 NAbs. A) The sensitivity of JR-FL E168K+N189A gp160ΔCT PV in WT and SOS formats was assessed with V2 NAbs and 39F. B) The effect of removing V2’ glycans at positions N188 and N189 on JR-FL E168K SOS sensitivity to V2 MAb PG9 were compared.
(TIF)

S3 Fig Global variation in V1V2 sequences. A logo plot was generated of residues 150 to 185 of the V1V2 loop of 4,582 HIV-1 Env sequences of the Los Alamos database. Asterisks indicate residues important for broad V2 MAb binding. Basic residues are shown in blue and acidic residues are shown in red.
(TIF)

S4 Fig Effects of mutations on JR-FL pseudovirus MAb sensitivities. The impact of key JR-FL mutations on MAb sensitivities to a range of concentrations of A) CH01 and its UCA (latter for only one mutant, as indicated), B) VRC34.01, C) 39F and D) PGT145. This data exemplifies MAb titrations that were used to create data for the IC50 dot plot shown in Fig 4B. The best mutant is highlighted in red.
(TIF)

S5 Fig Models of glycan maturation of JR-FL gp120 and membrane trimers. Related to Figs 5 and S6 and S1 Data and analysis. Models of JR-FL gp120 monomer and trimers (both derived from pdb 6MYY) show the glycan scores, using the same format as in Fig 5A. These models were created from data in S1 Data and analysis. Models include gp120 monomer and a SOS E168K+N189A parent sample, both dated 11-11-19 (parts A and B), followed by 10 samples including the parent, mutants and a CH01 complexed sample, dated 4-24-21 (parts C-L). In each case, mutant locations are indicated by bold outlined text at the affected glycan site.
(PDF)

S6 Fig Models of glycan maturation differences between JR-FL Env sample pairs. Related to Figs 5B and S5 and S1 Data and analysis. Trimer models depict glycan score differences between sample pairs. Increases in glycan maturation are depicted in progressively bolder hues of blue, while decreases in maturation are indicated in progressively bolder hues of red. Unchanged glycan scores are shown in yellow. Mutant locations are indicated as in S5 Fig.
(PDF)

S7 Fig Effects of mutations on Q23 pseudovirus MAb sensitivities. The impact of key Q23 mutations on sensitivities to A) CH01 B) CH01 UCA, C) PGT145, and D) 14e, using the pQC-Fluc assay. This data exemplifies the MAb titrations used to create the IC50 dot plot in Fig 7B.
(TIF)

S8 Fig VLP stimulation of CH01 UCA-expressing B-cells. A) C57BL/6J WT or CH01 UCA double KI splenocytes were stained with anti-B220, anti-CD19 MAbs and WITO-SOSIP HIV Env tetramers to verify CH01 UCA expression on naïve splenic B-cells by SOSIP binding. B) Mice splenocytes were stained as above, loaded with Fluo-4 and resuspended in calcium-containing HBSS. Cells were then incubated with anti-IgM F(ab′)2, or graded doses of bald VLPs or Q23 SOS VLPs produced in either 293T cells or GnT1- 293S cells.
(TIF)

S9 Fig Effects of mutations on WITO pseudovirus MAb sensitivities and expression. The impact of key WITO mutations on sensitivities to A) CH01 or 39F (by pQC-Fluc assay). This data exemplifies the MAb titrations used to create data points for the IC50 dot plot in Fig 7D. Assays were repeated with consistent results at least twice. B) Gp120 expression of the WITO mutants was assayed by SDS-PAGE-Western blot. C) Logo plot showing the frequency of the N49 glycan (shown as a magenta O) in various clades.
(TIF)

S10 Fig Effects of mutations on T250 pseudovirus MAb sensitivities. The impact of key T250 mutations on sensitivities to A) CH01, B) CH01 UCA, C) 14e, D) PGT145, using the pQC-Fluc assay. This data exemplifies the MAb titrations that were used to create data points for the IC50 dot plot in Fig 8B.
(TIF)

S11 Fig Effects of mutations on CE217 neutralization sensitivities and expression. A) Gp120 expression of CE217 mutants was assayed by SDS-PAGE-Western blot. B) CH01, C) CH01 UCA, D) 14e and E) PGT145 were titrated against key CE217 mutants in the NL-Luc assay. This data exemplifies the MAb titrations that were used to create data points for the IC50 dot plot in Fig 8D.
(TIF)

S12 Fig Effects of mutations on AC10, sc422 and KNH1144 neutralization sensitivities. Various MAbs were titrated against A) AC10, B) sc422 and C) KNH1144 mutants, as indicated, in the pQC-Fluc assay.
(TIF)

S1 Data and analysis Glycopeptide assignments at JR-FL Env sequon positions in various formats. Related to Fig 5. Heat maps are shown based on glycopeptide analysis of JR-FL VLP or gp120 monomer samples generated from two analyses of separate samples dated 11-11-19 and 4-24-21. To calibrate the extent of glycan maturation, each glycan type was assigned a numerical code from 1 to 19. Each number was multiplied by its percentage at each sequon to determine the mean glycan number (maturation state). These scores were used to generate the models in S5 Fig. Also shown are percentages of core glycan and sequon skipping. Differences in glycan maturation at each site between pairs of samples were calculated by subtracting glycan scores of one sample (usually the parent) from the scores of the other. These changes in glycan maturation were used to generate the models in S6 Fig.
(XLSX)

S1 Text Verification of V3 MAb epitopes to monitor misfolding in a post-sCD4 neutralization assay. (DOCX)

S2 Text Effects of JR-FL mutations on Env mobility and endo H sensitivity in SDS-PAGE. (DOCX)

S3 Text Attempts to improve gp160 processing. (DOCX)

S4 Text Engineering strains c1080 and 6101. (DOCX)

S5 Text Other engineering approaches to try to improve membrane Env expression. (DOCX)

S6 Text Detailed vFC methods. (DOCX)

S1 Table MIFlowCyt checklist and EV. (XLSX)
