# Supplementary figures and images for: Engineering well-expressed, V2-immunofocusing HIV-1 envelope glycoprotein membrane trimers for use in heterologous prime-boost vaccine regimens
Source: PLoS Pathog. 2021 Oct 22;17(10):e1009807. doi: 10.1371/journal.ppat.1009807 (PMC8565784; doi:10.1371/journal.ppat.1009807)

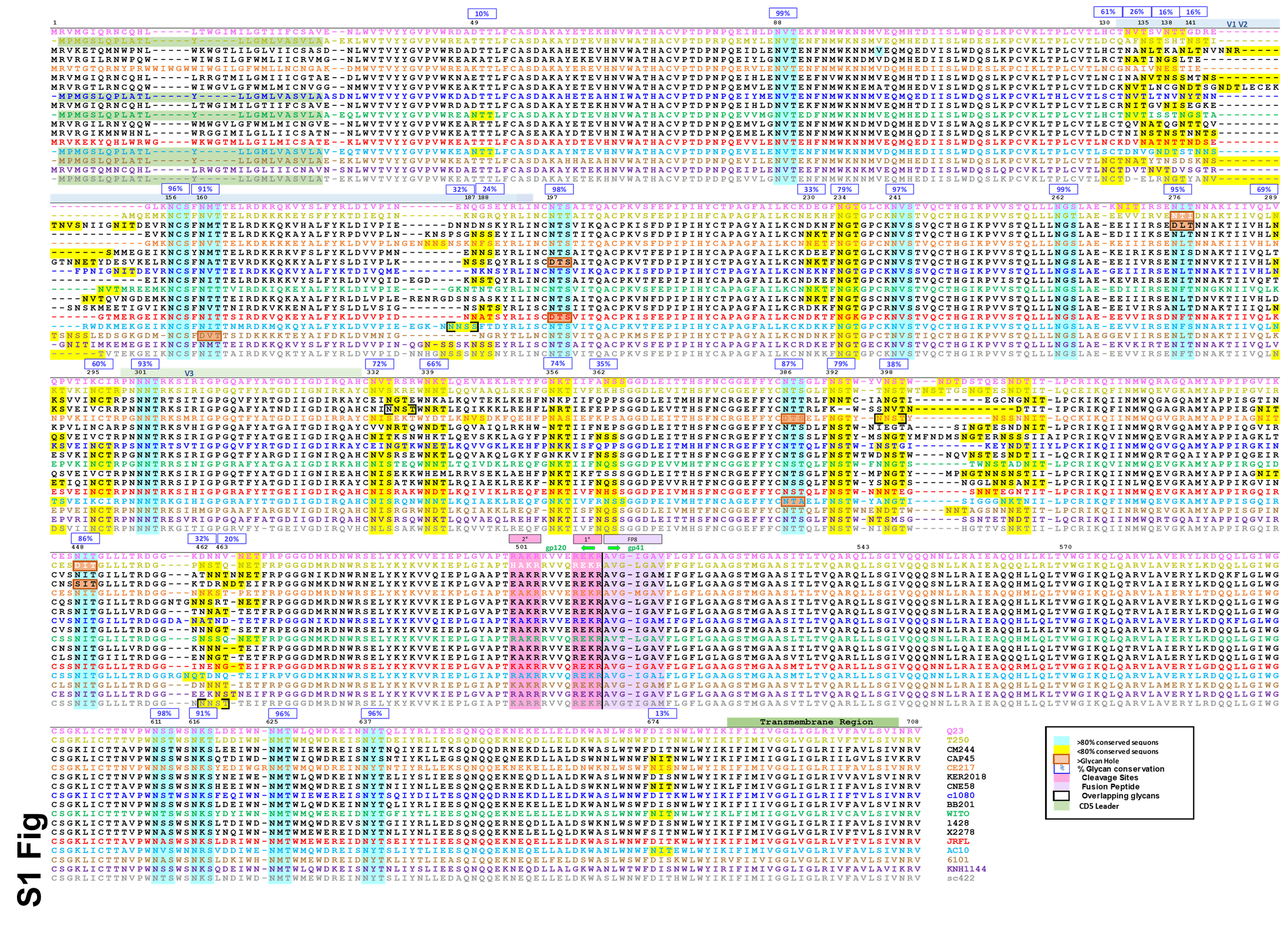

Supplement: S1 Fig — Amino acids are numbered according to the prototype HXB2 sequence (Genbank: AAB50262.1). Conserved sequons (>80%) are highlighted in cyan; variable sequons (<80%) are highlighted in yellow. Glycan holes, in which >80% conserved glycans are missing, are shown in orange. Primary and secondary gp120-gp41 furin cleavage sites are shown in magenta. FP is highlighted in lavender. Overlapping sequons are boxed in black. CD5 signal peptide on certain strains is highlighted as green (AA1-31). (TIF) [file ppat.1009807.s001.tif]

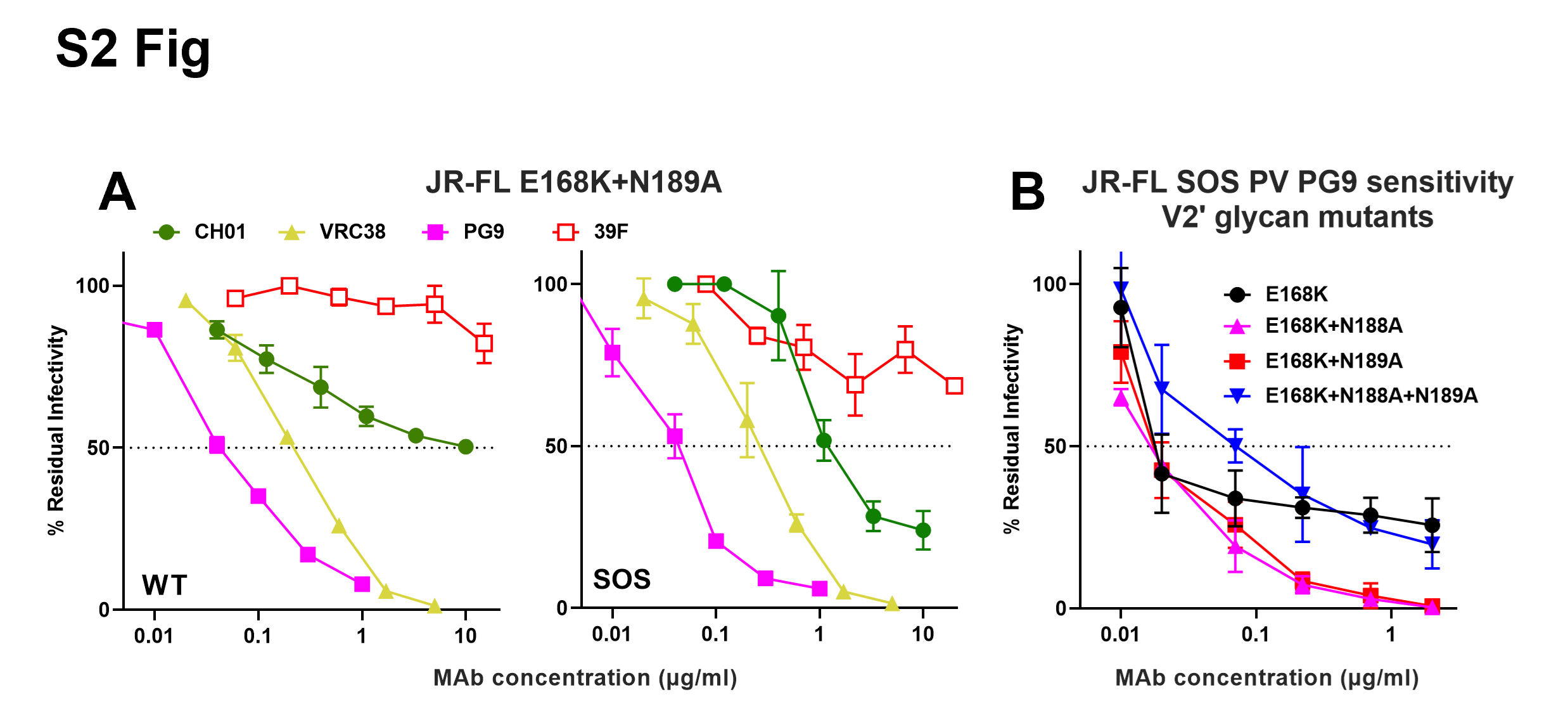

Supplement: S2 Fig — A) The sensitivity of JR-FL E168K+N189A gp160ΔCT PV in WT and SOS formats was assessed with V2 NAbs and 39F. B) The effect of removing V2’ glycans at positions N188 and N189 on JR-FL E168K SOS sensitivity to V2 MAb PG9 were compared. (TIF) [file ppat.1009807.s002.tif]

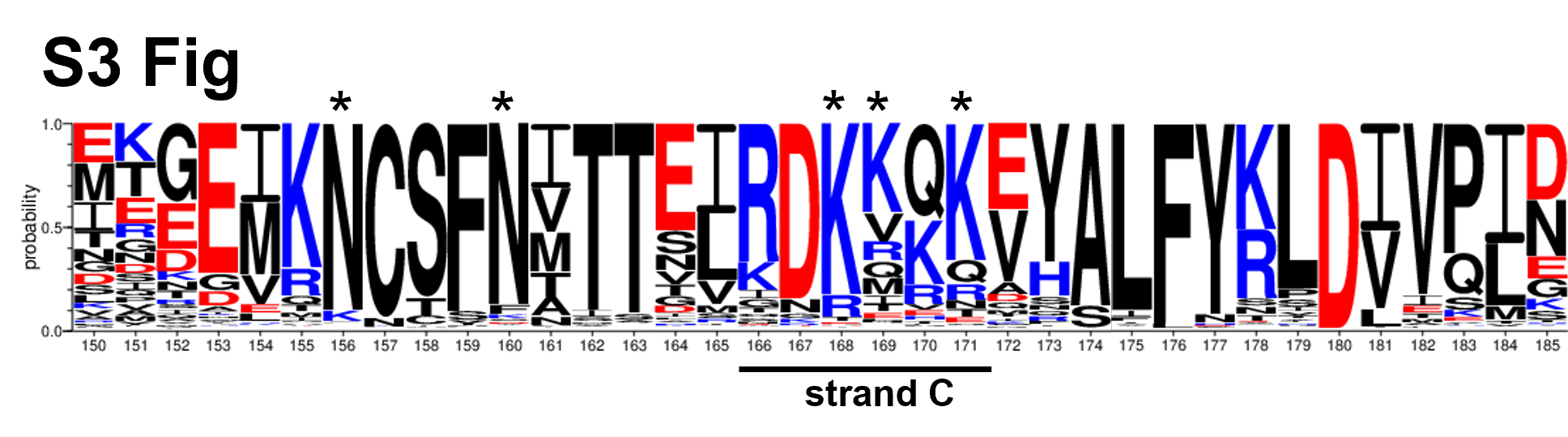

Supplement: S3 Fig — A logo plot was generated of residues 150 to 185 of the V1V2 loop of 4,582 HIV-1 Env sequences of the Los Alamos database. Asterisks indicate residues important for broad V2 MAb binding. Basic residues are shown in blue and acidic residues are shown in red. (TIF) [file ppat.1009807.s003.tif]

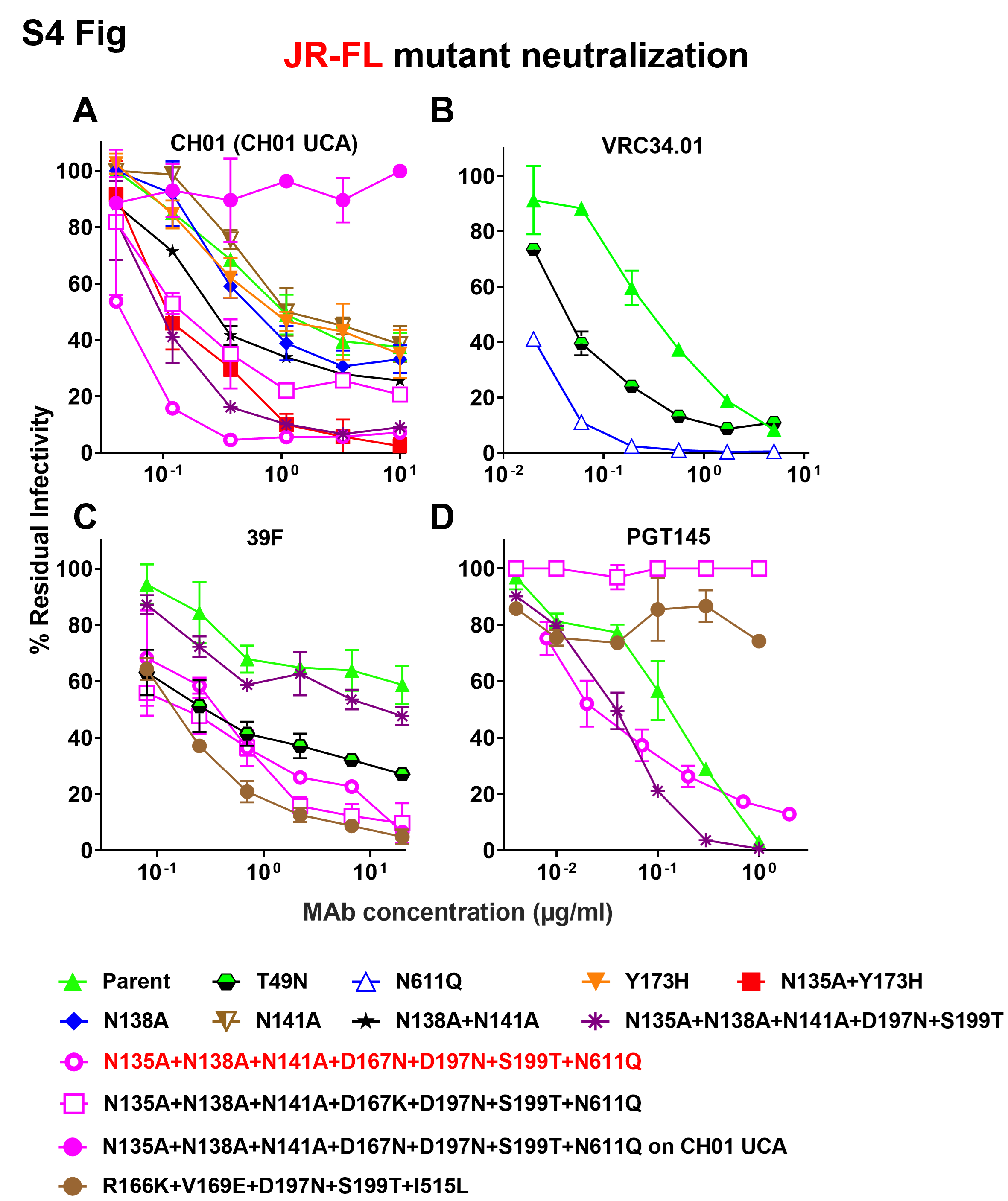

Supplement: S4 Fig — The impact of key JR-FL mutations on MAb sensitivities to a range of concentrations of A) CH01 and its UCA (latter for only one mutant, as indicated), B) VRC34.01, C) 39F and D) PGT145. This data exemplifies MAb titrations that were used to create data for the IC50 dot plot shown in Fig 4B. The best mutant is highlighted in red. (TIF) [file ppat.1009807.s004.tif]

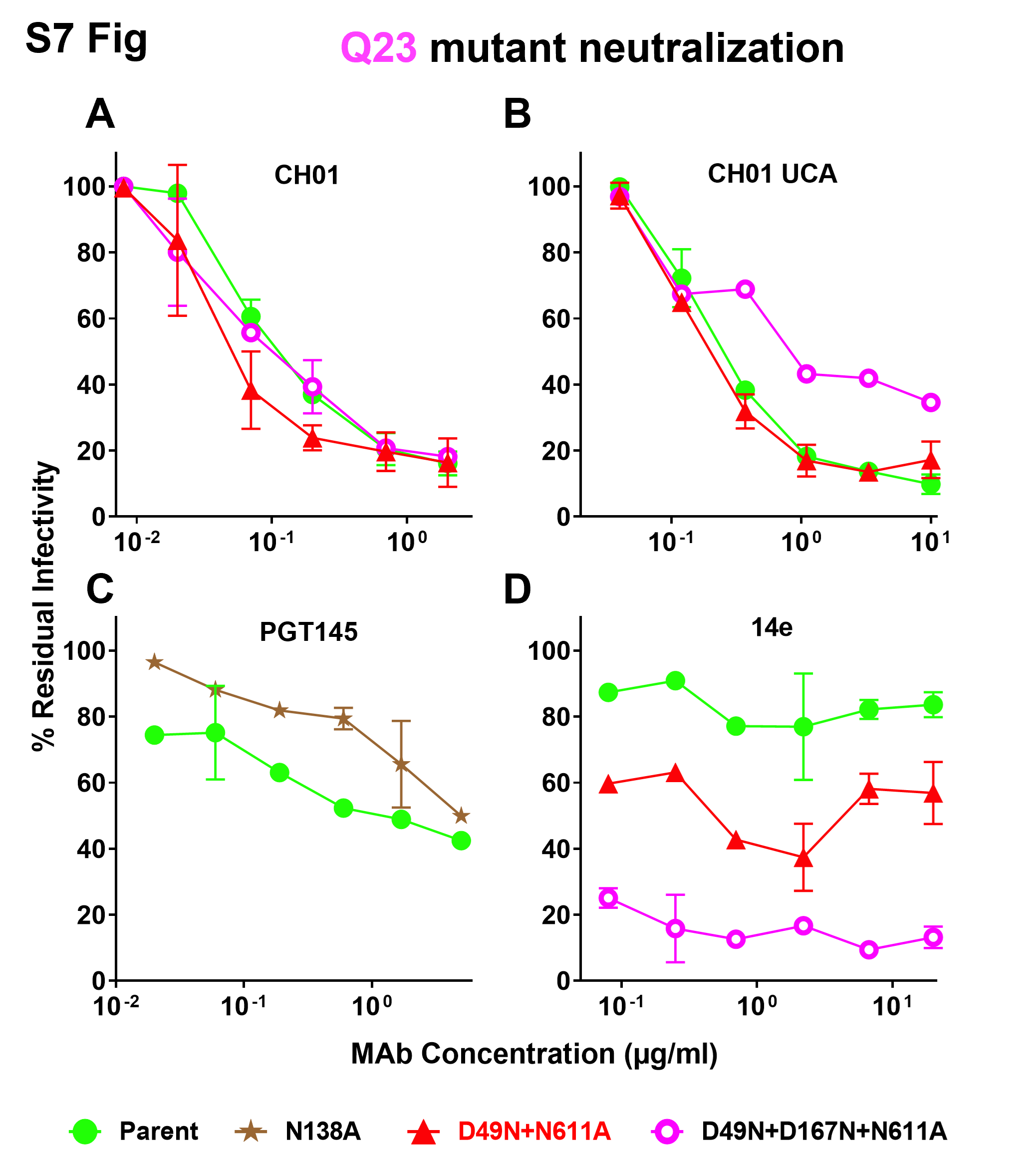

Supplement: S7 Fig — The impact of key Q23 mutations on sensitivities to A) CH01 B) CH01 UCA, C) PGT145, and D) 14e, using the pQC-Fluc assay. This data exemplifies the MAb titrations used to create the IC50 dot plot in Fig 7B. (TIF) [file ppat.1009807.s007.tif]

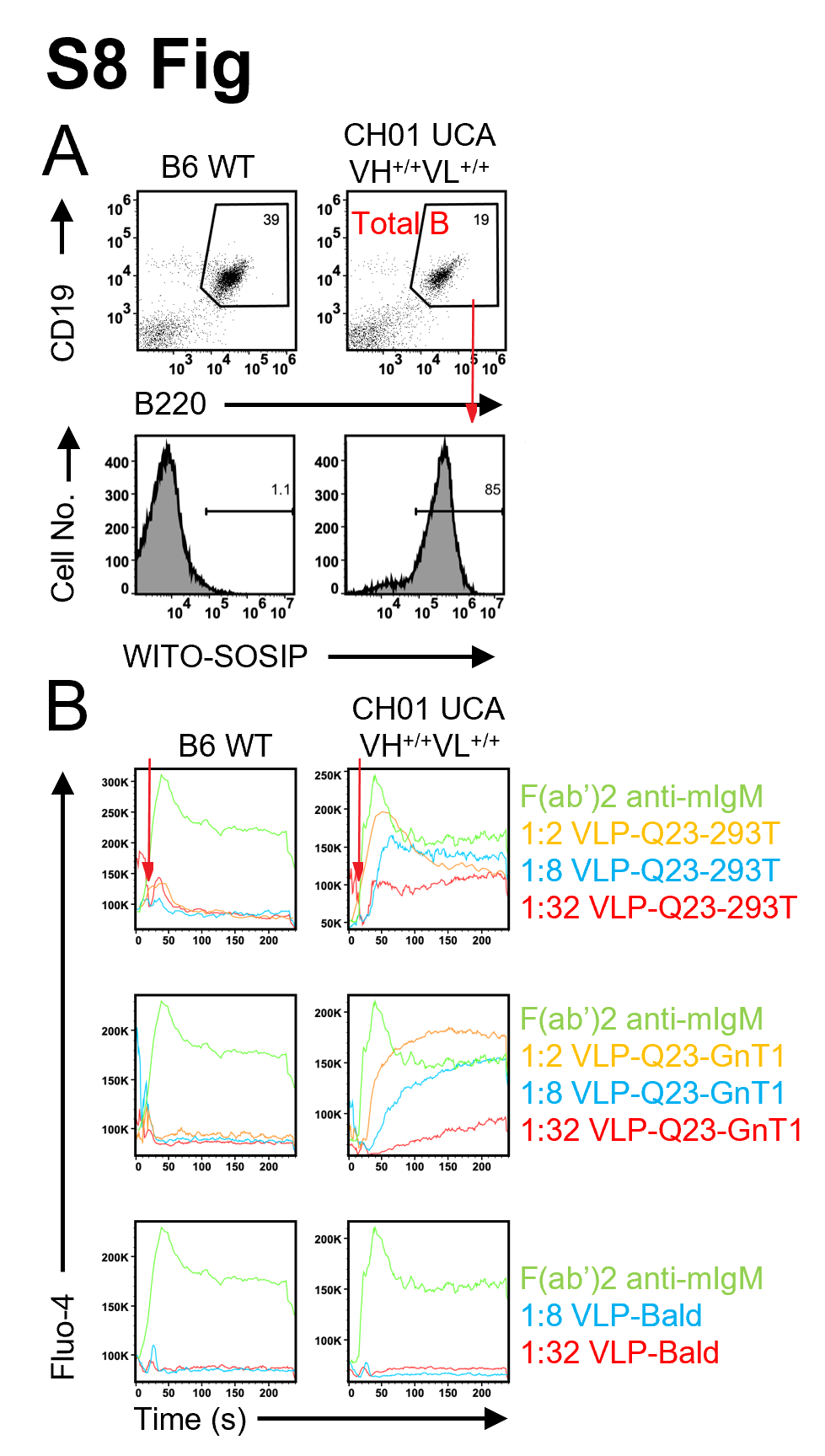

Supplement: S8 Fig — A) C57BL/6J WT or CH01 UCA double KI splenocytes were stained with anti-B220, anti-CD19 MAbs and WITO-SOSIP HIV Env tetramers to verify CH01 UCA expression on naïve splenic B-cells by SOSIP binding. B) Mice splenocytes were stained as above, loaded with Fluo-4 and resuspended in calcium-containing HBSS. Cells were then incubated with anti-IgM F(ab′)2, or graded doses of bald VLPs or Q23 SOS VLPs produced in either 293T cells or GnT1- 293S cells. (TIF) [file ppat.1009807.s008.tif]

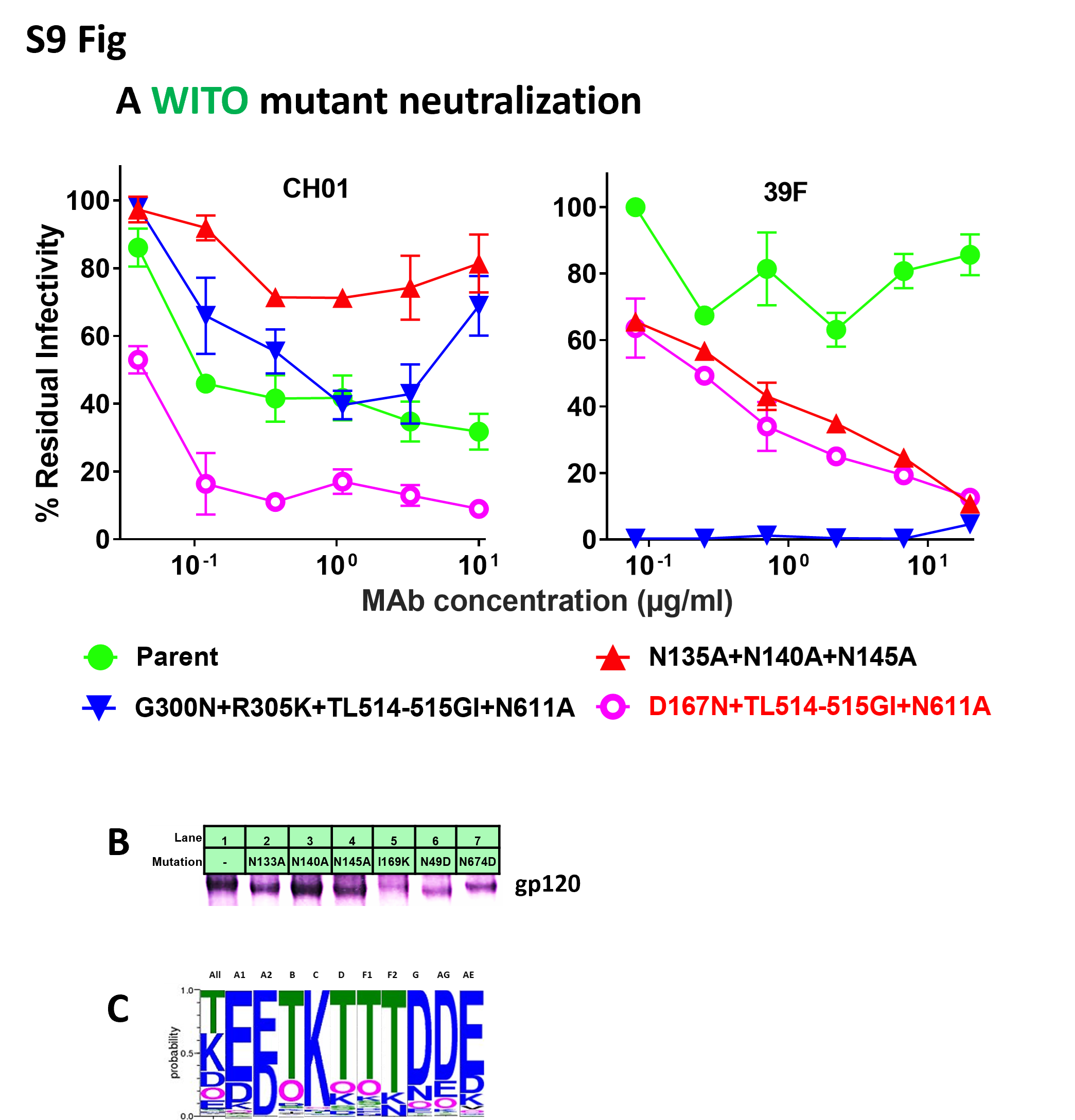

Supplement: S9 Fig — The impact of key WITO mutations on sensitivities to A) CH01 or 39F (by pQC-Fluc assay). This data exemplifies the MAb titrations used to create data points for the IC50 dot plot in Fig 7D. Assays were repeated with consistent results at least twice. B) Gp120 expression of the WITO mutants was assayed by SDS-PAGE-Western blot. C) Logo plot showing the frequency of the N49 glycan (shown as a magenta O) in various clades. (TIF) [file ppat.1009807.s009.tif]

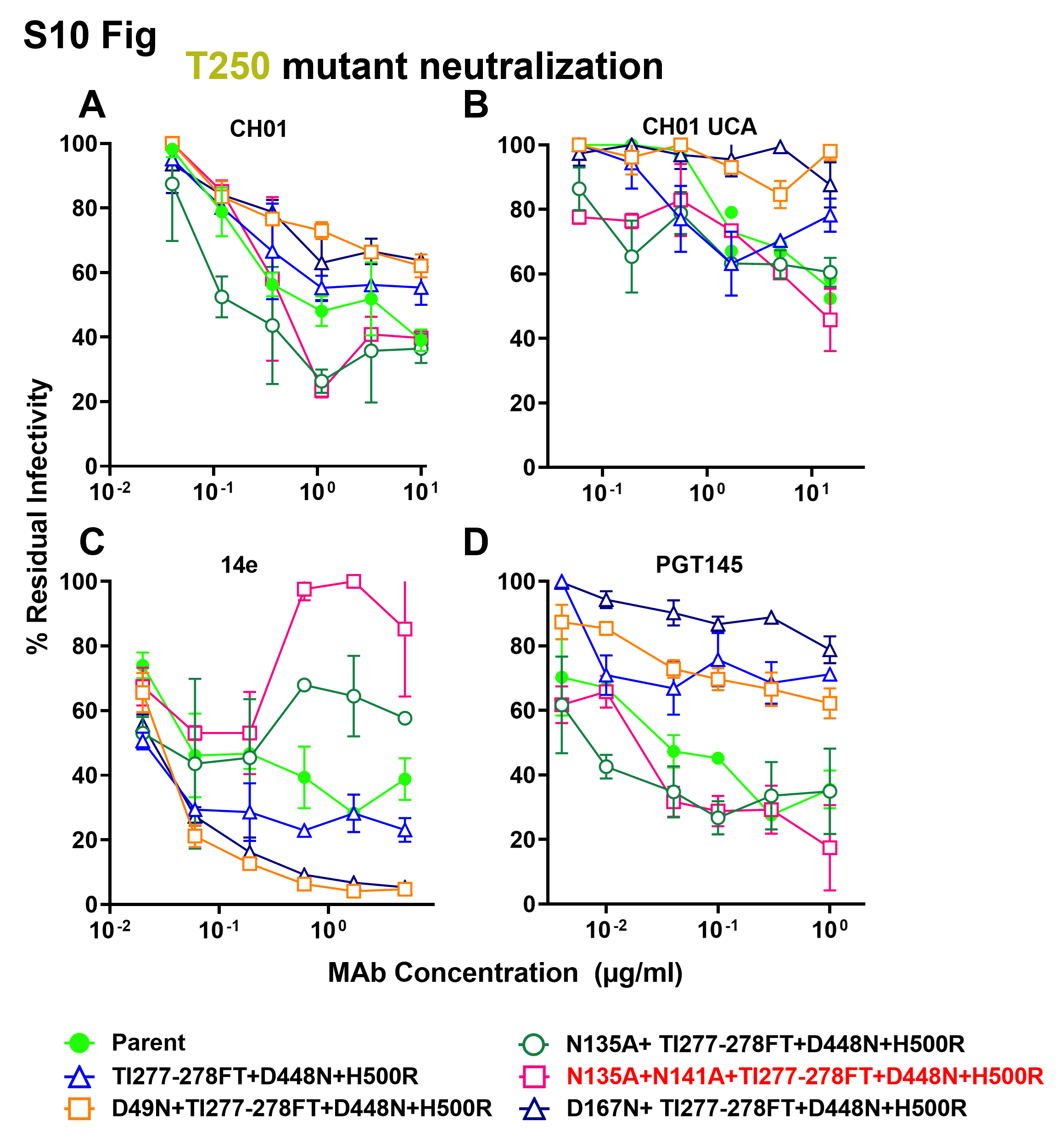

Supplement: S10 Fig — The impact of key T250 mutations on sensitivities to A) CH01, B) CH01 UCA, C) 14e, D) PGT145, using the pQC-Fluc assay. This data exemplifies the MAb titrations that were used to create data points for the IC50 dot plot in Fig 8B. (TIF) [file ppat.1009807.s010.tif]

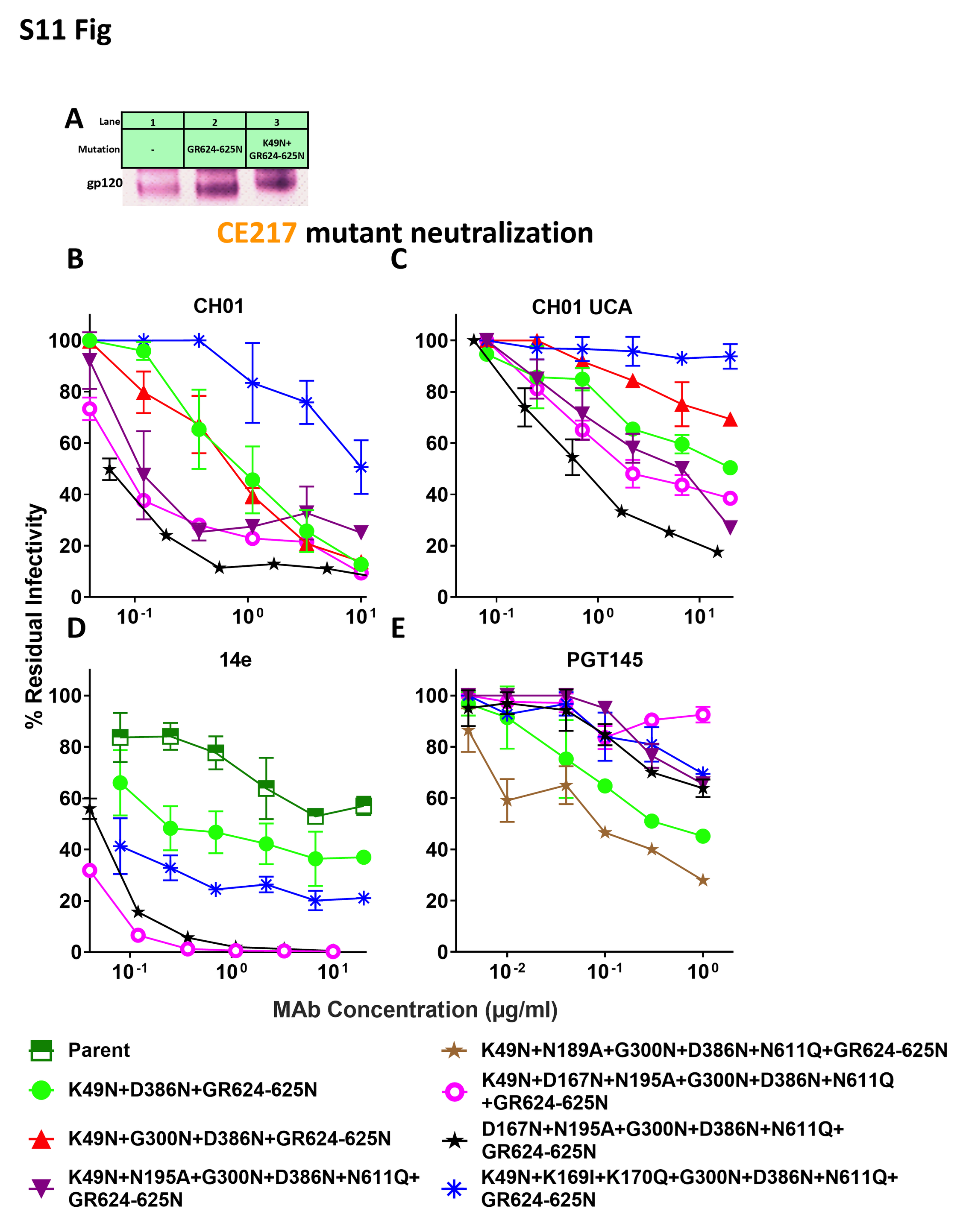

Supplement: S11 Fig — A) Gp120 expression of CE217 mutants was assayed by SDS-PAGE-Western blot. B) CH01, C) CH01 UCA, D) 14e and E) PGT145 were titrated against key CE217 mutants in the NL-Luc assay. This data exemplifies the MAb titrations that were used to create data points for the IC50 dot plot in Fig 8D. (TIF) [file ppat.1009807.s011.tif]

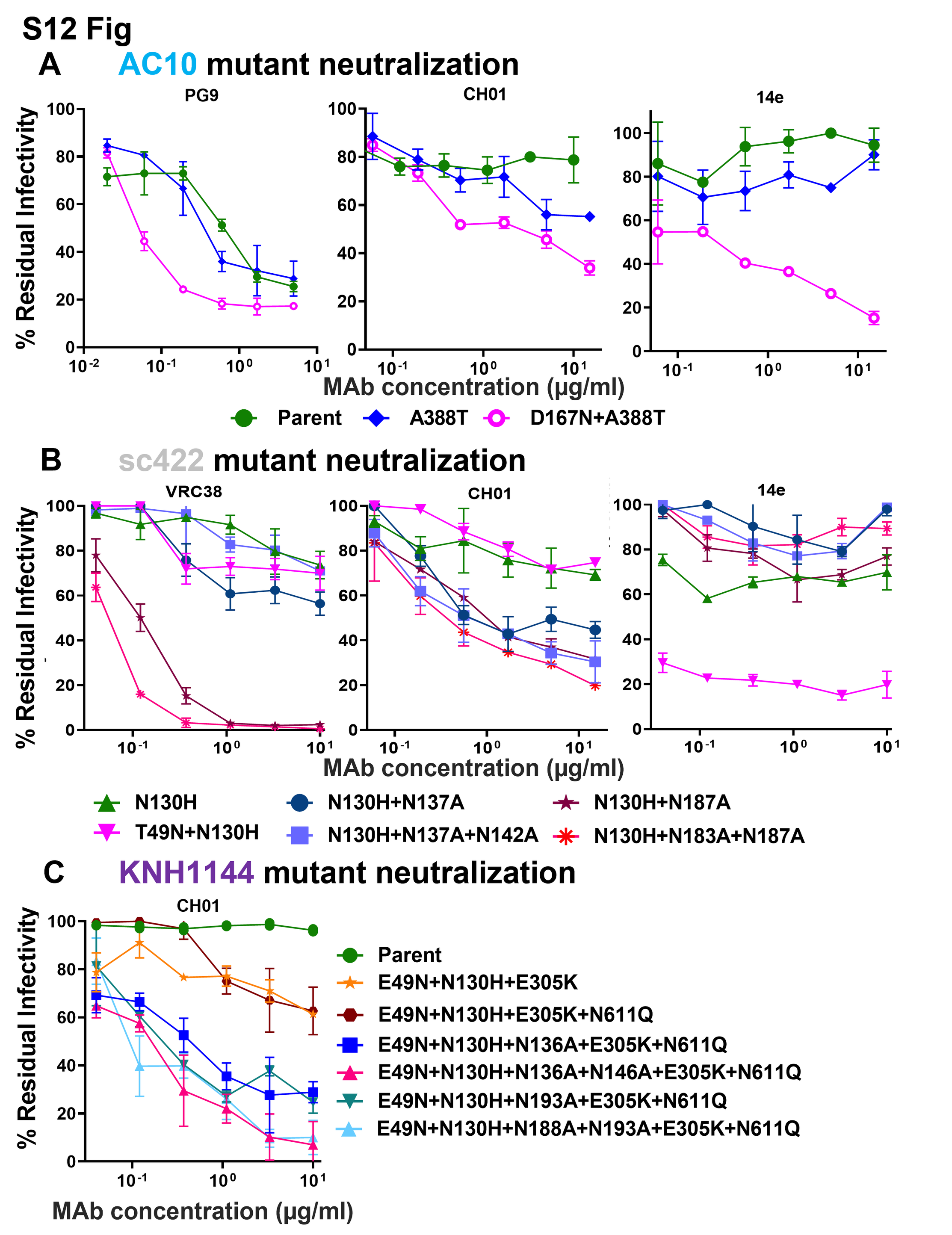

Supplement: S12 Fig — Various MAbs were titrated against A) AC10, B) sc422 and C) KNH1144 mutants, as indicated, in the pQC-Fluc assay. (TIF) [file ppat.1009807.s012.tif]
