# Supplementary material for: Engineering well-expressed, V2-immunofocusing HIV-1 envelope glycoprotein membrane trimers for use in heterologous prime-boost vaccine regimens
Source: PLoS Pathog. 2021 Oct 22;17(10):e1009807. doi: 10.1371/journal.ppat.1009807 (PMC8565784; doi:10.1371/journal.ppat.1009807)

S5 Fig (page 1)

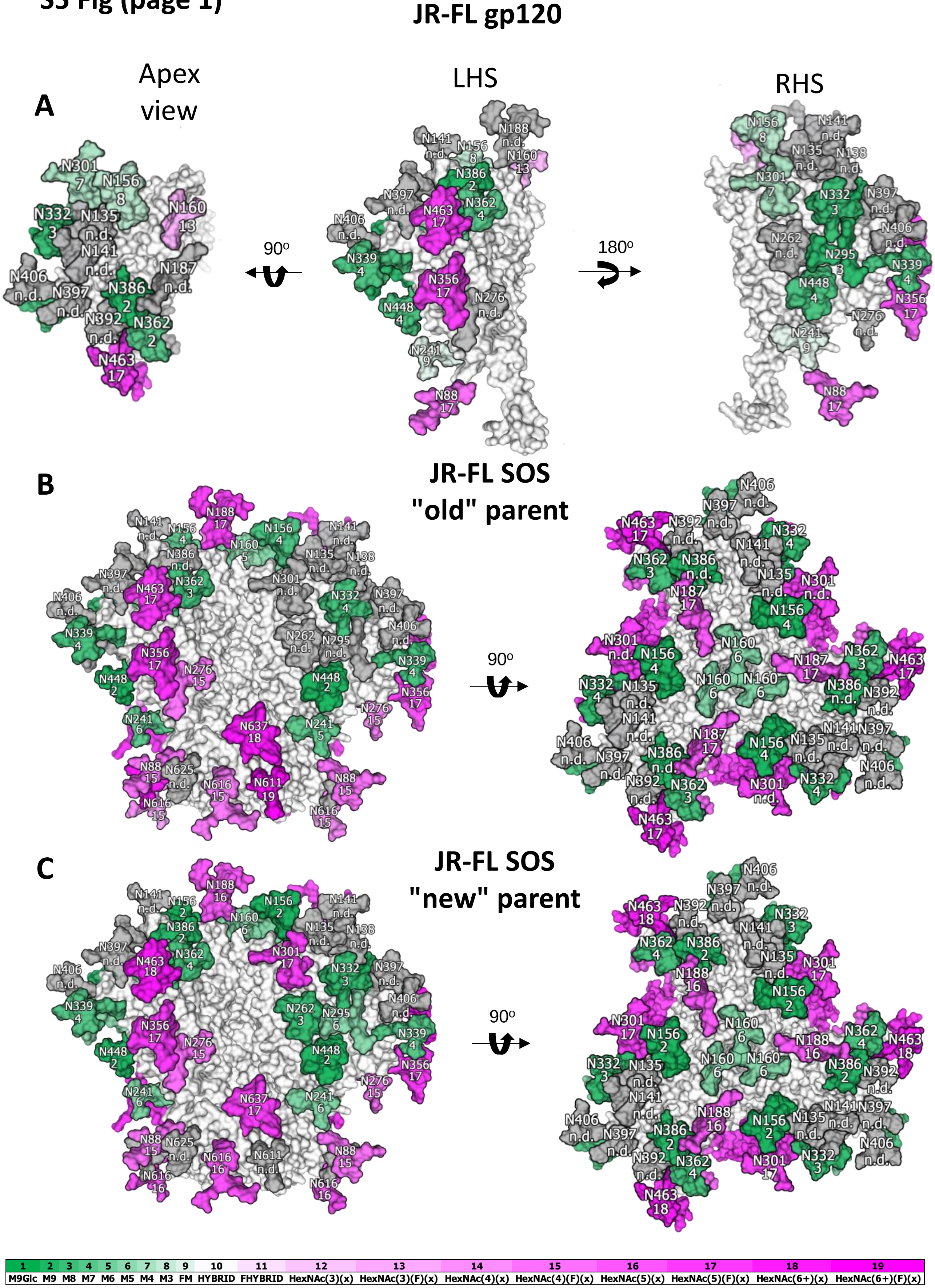
JR-FL gp120
A
Apex view
LHS
RHS
90°
180°
B
JR-FL SOS "old" parent
90°
C
JR-FL SOS "new" parent
90°
1 M9Glc
2 M9
3 M8
4 M7
5 M6
6 M5
7 M4
8 M3
9 FM
10 HYBRID
11 FHYBRID
12 HexNAc(3)(x)
13 HexNAc(3)(F)(x)
14 HexNAc(4)(x)
15 HexNAc(4)(F)(x)
16 HexNAc(5)(x)
17 HexNAc(5)(F)(x)
18 HexNAc(6+)(x)
19 HexNAc(6+)(F)(x)

S5 Fig (page 2)

D

Sequon optimization

2. S158T

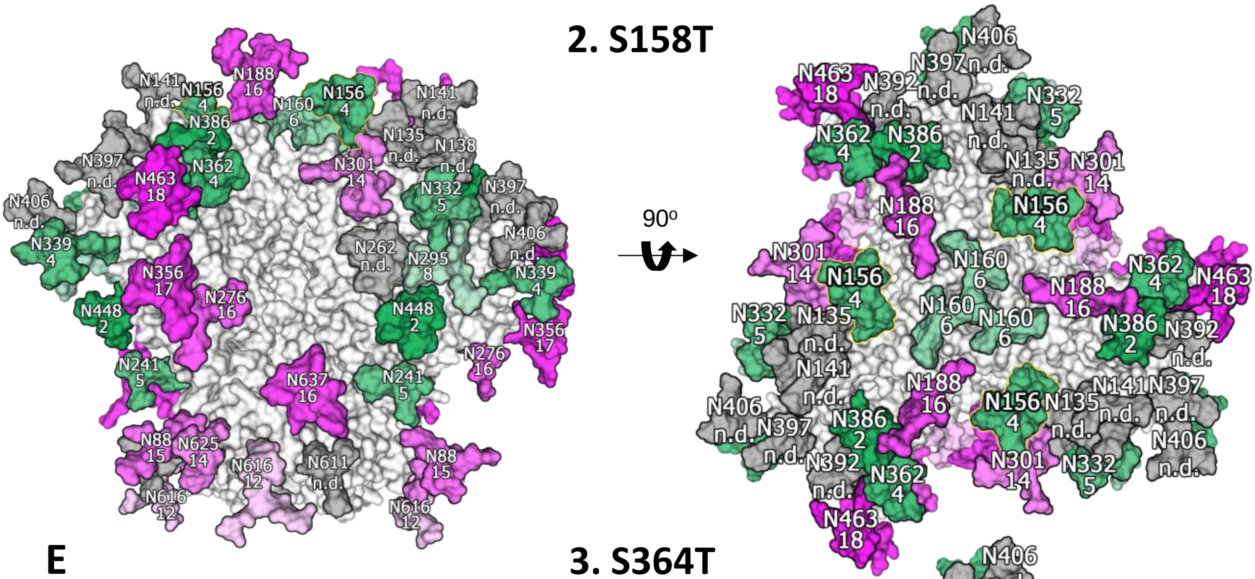

E

3. S364T

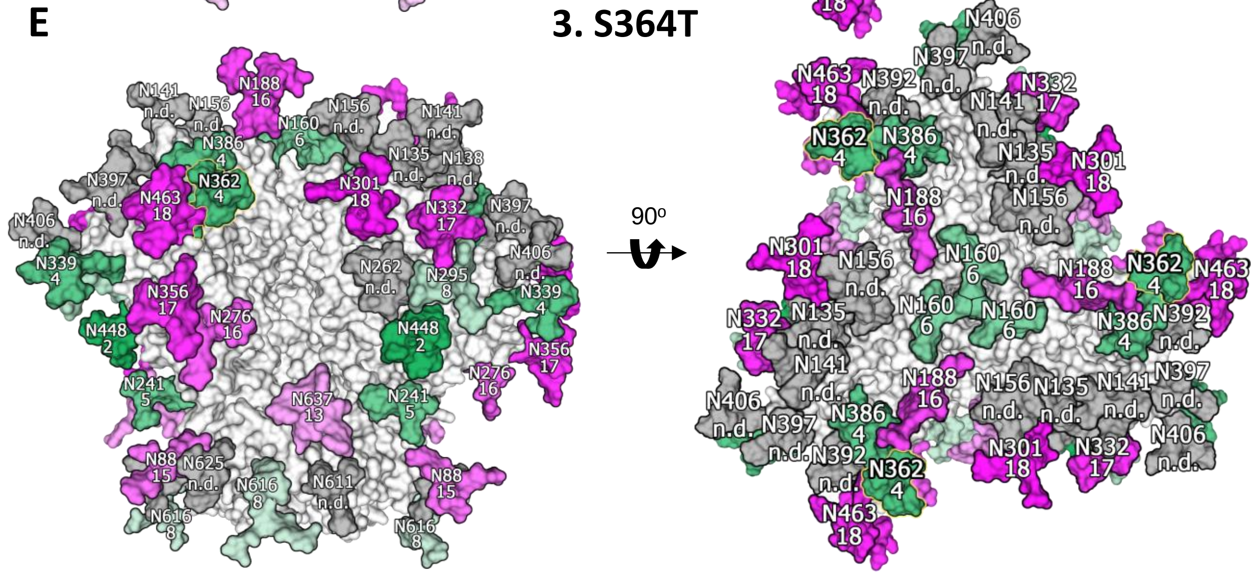

1	2	3	4	5	6	7	8	9	10	11	12	13	14	15	16	17	18	19
M9Glc	M9	M8	M7	M6	M5	M4	M3	FM	HYBRID	FHYBRID	HexNAc(3)(x)	HexNAc(3)(F)(x)	HexNAc(4)(x)	HexNAc(4)(F)(x)	HexNAc(5)(x)	HexNAc(5)(F)(x)	HexNAc(6+)(x)	HexNAc(6+)(F)(x)

S5 Fig (page 3)

D197N knock in

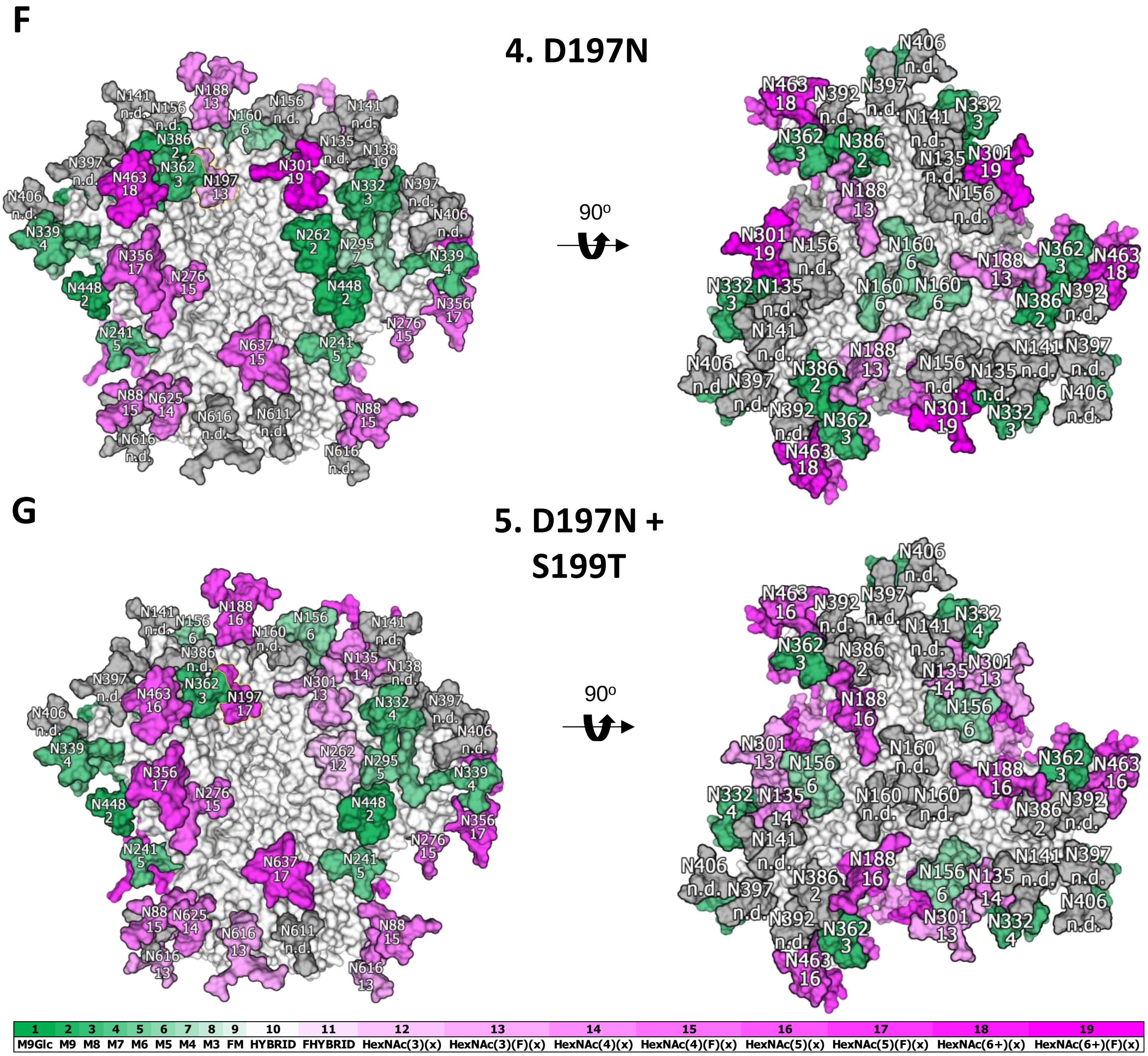

S5 Fig (page 4)

T49N and N611Q

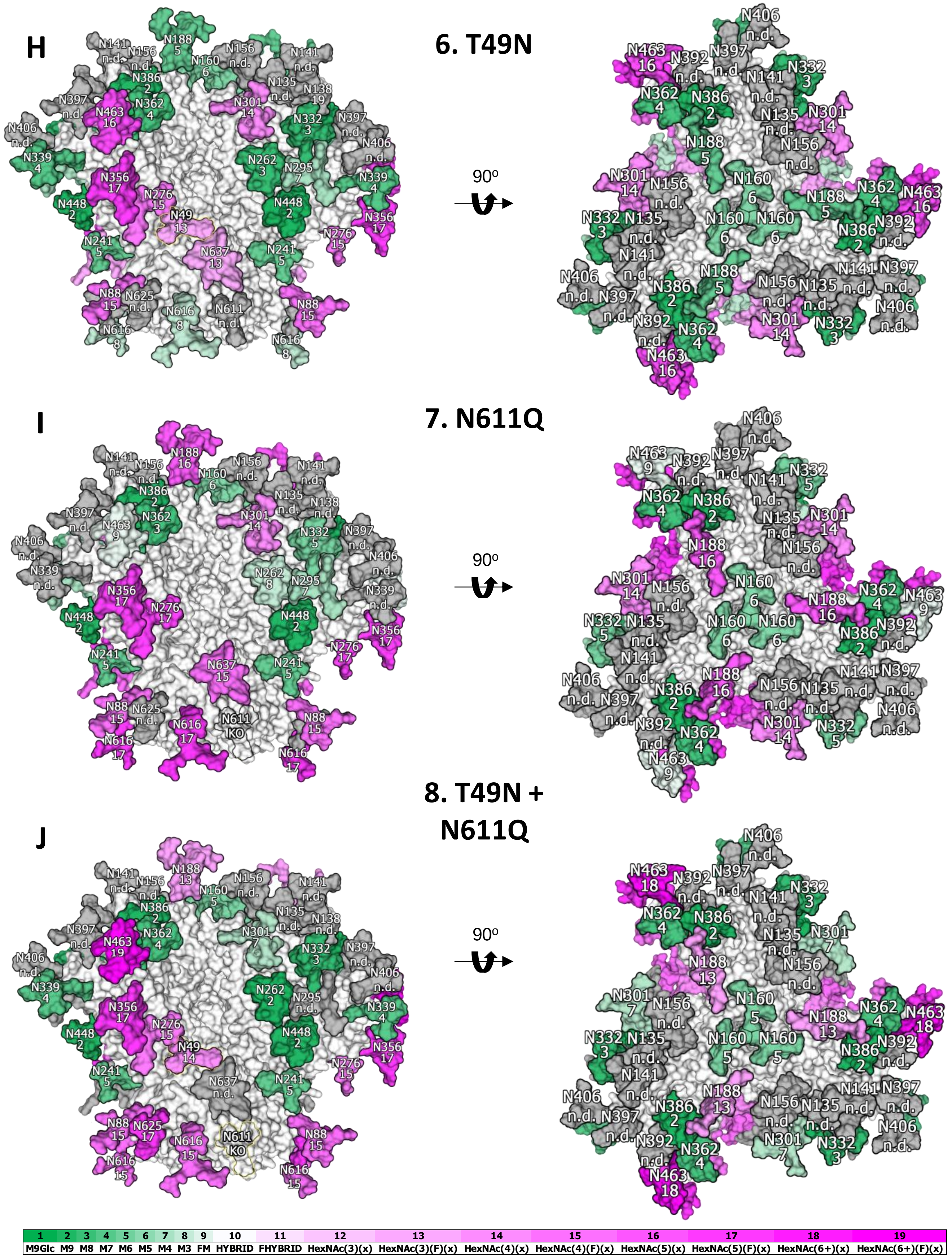

S5 Fig (page 5)

K

CH01-bound glycovariants

9. N138A + N141A

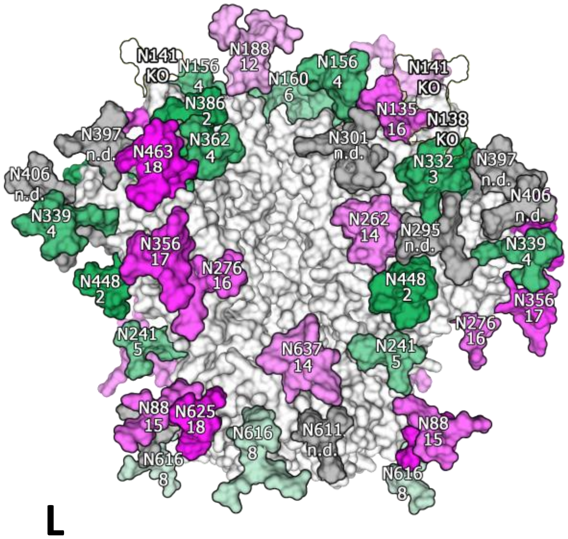

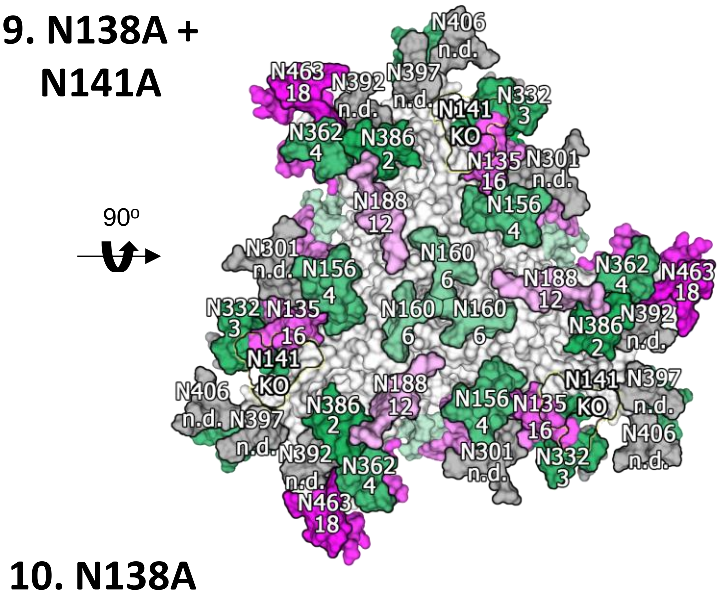

L

10. N138A + N141A + CH01

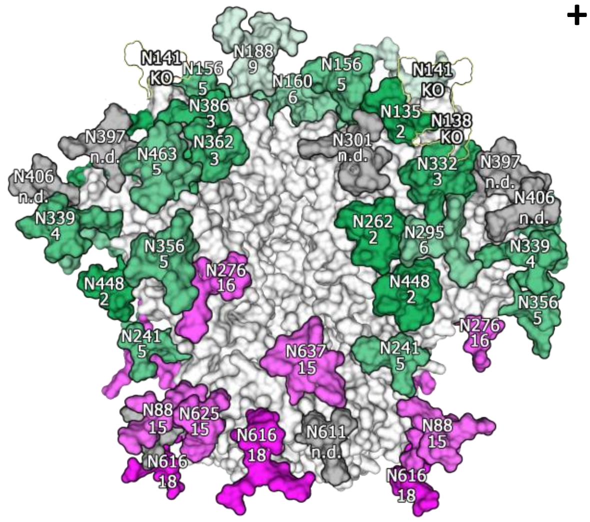

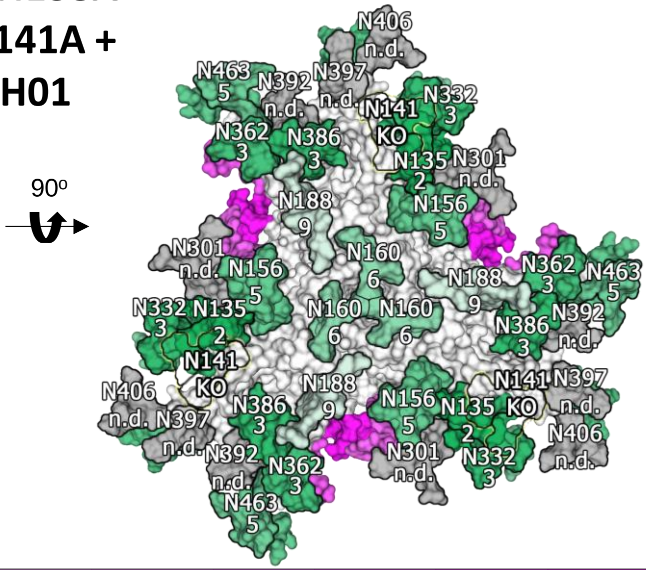

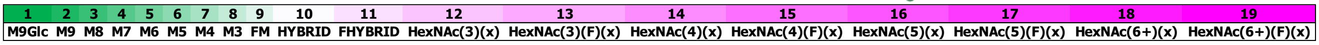

Supplement: S5 Fig — Related to Figs 5 and S6 and S1 Data and analysis. Models of JR-FL gp120 monomer and trimers (both derived from pdb 6MYY) show the glycan scores, using the same format as in Fig 5A. These models were created from data in S1 Data and analysis. Models include gp120 monomer and a SOS E168K+N189A parent sample, both dated 11-11-19 (parts A and B), followed by 10 samples including the parent, mutants and a CH01 complexed sample, dated 4-24-21 (parts C-L). In each case, mutant locations are indicated by bold outlined text at the affected glycan site. (PDF) [file ppat.1009807.s005.pdf]
