# Supplementary material for: Engineering well-expressed, V2-immunofocusing HIV-1 envelope glycoprotein membrane trimers for use in heterologous prime-boost vaccine regimens
Source: PLoS Pathog. 2021 Oct 22;17(10):e1009807. doi: 10.1371/journal.ppat.1009807 (PMC8565784; doi:10.1371/journal.ppat.1009807)

S6 Fig (page 1)

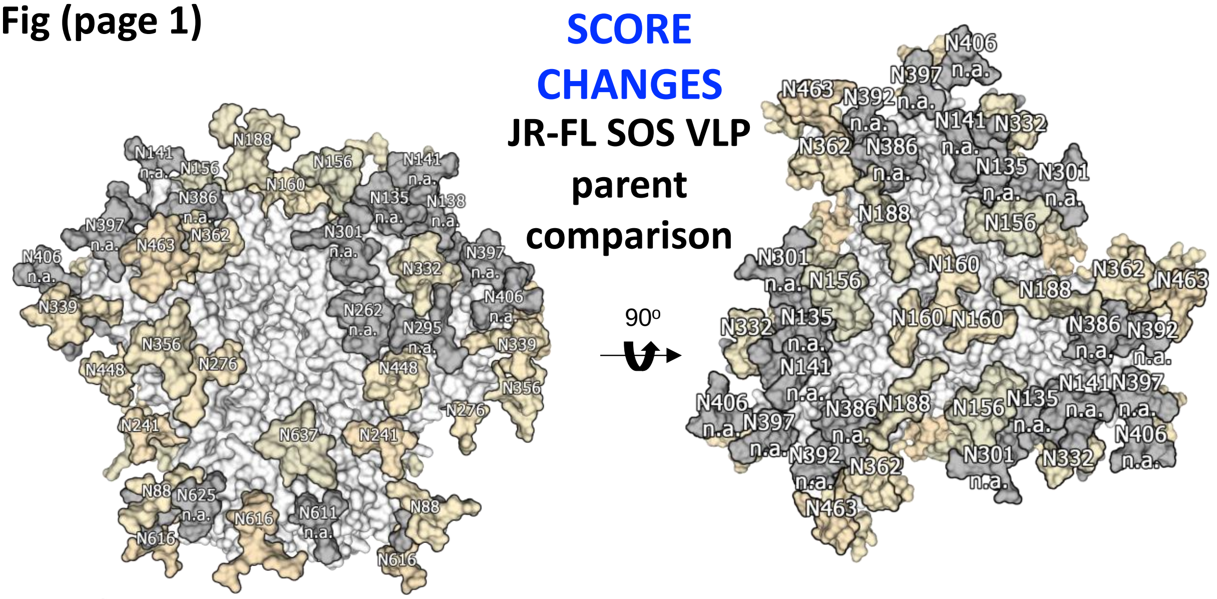

JR-FL gp120 vs "new" Parent

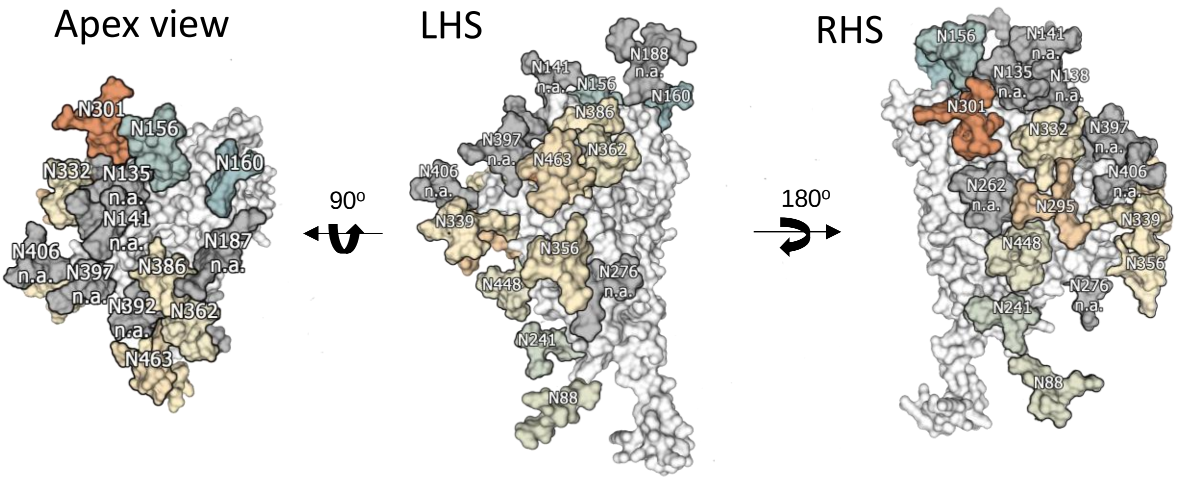

JR-FL gp120 vs "old" Parent

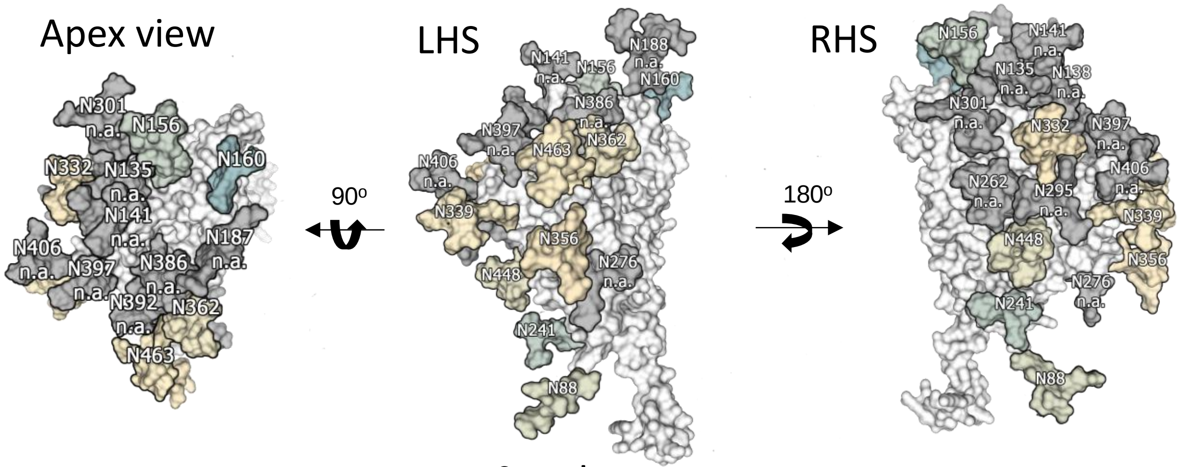

Score change

-20.00	-15.00	-10.00	-5.00	0.00	5.00	10.00	15.00	20.00

Decrease in Processing

Increase in Processing

S6 Fig (page 2)

SCORE CHANGES

S158T vs Parent

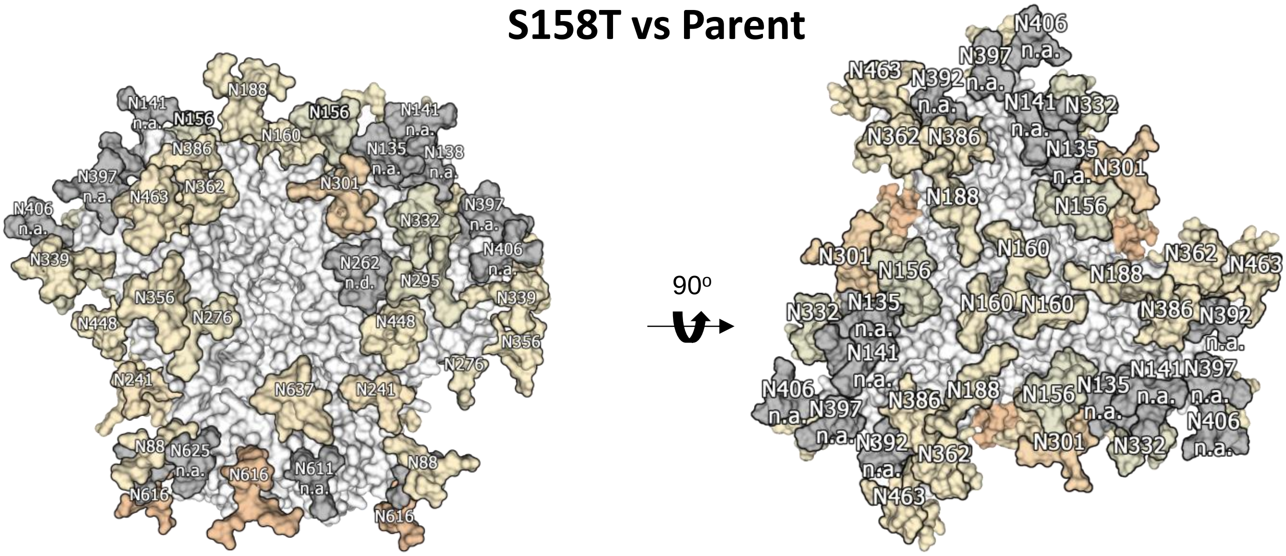

S364T vs Parent

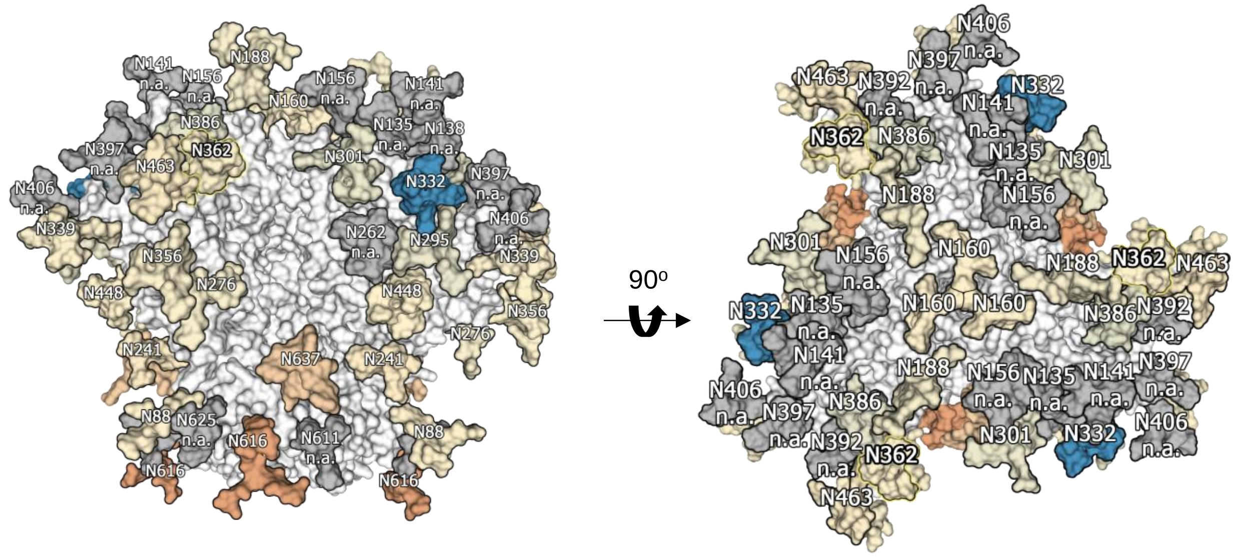

Score change

-20.00	-15.00	-10.00	-5.00	0.00	5.00	10.00	15.00	20.00

Decrease in Processing

Increase in Processing

S6 Fig (page 3)

SCORE CHANGES

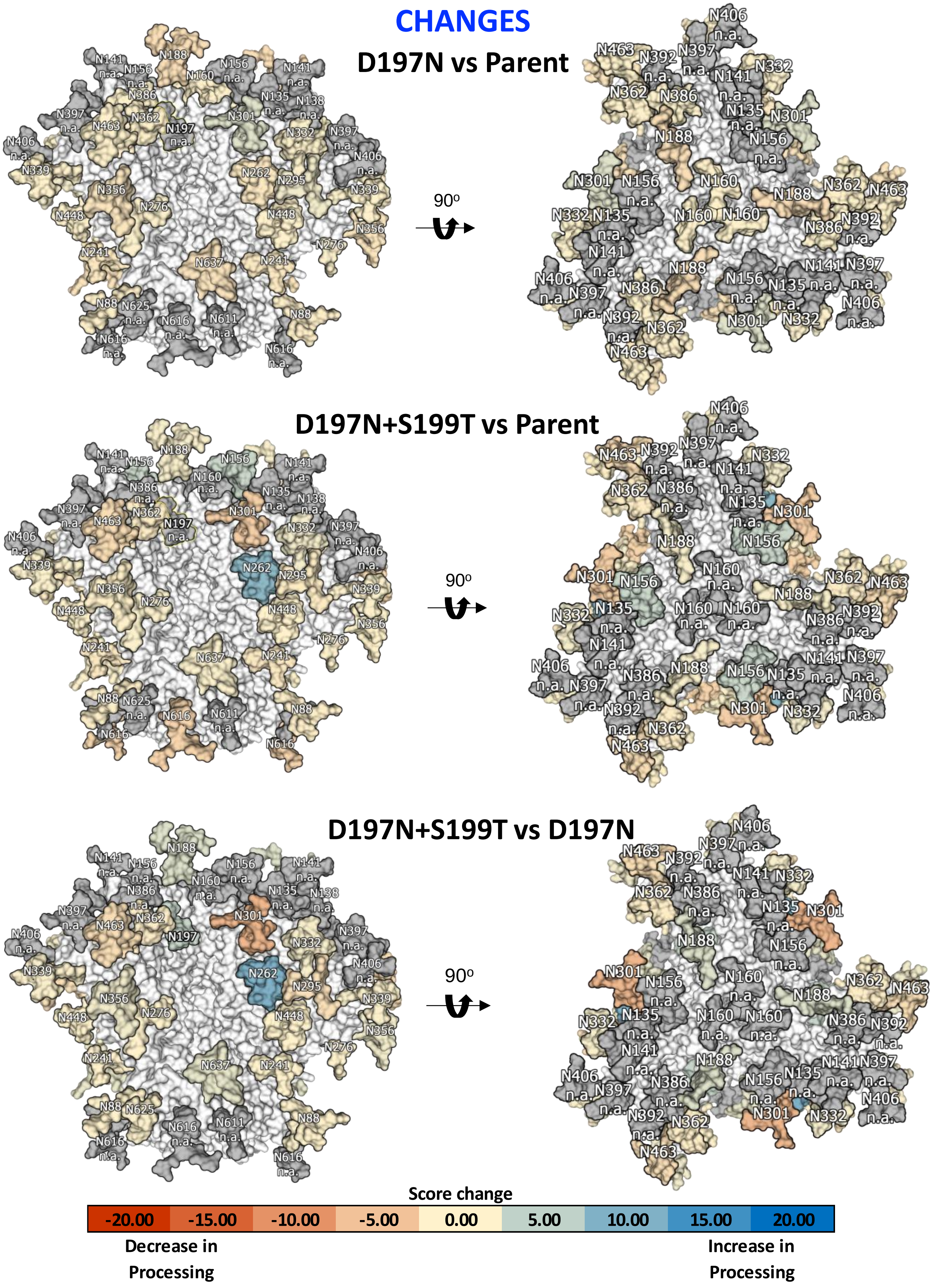

S6 Fig (page 4)

SCORE CHANGES

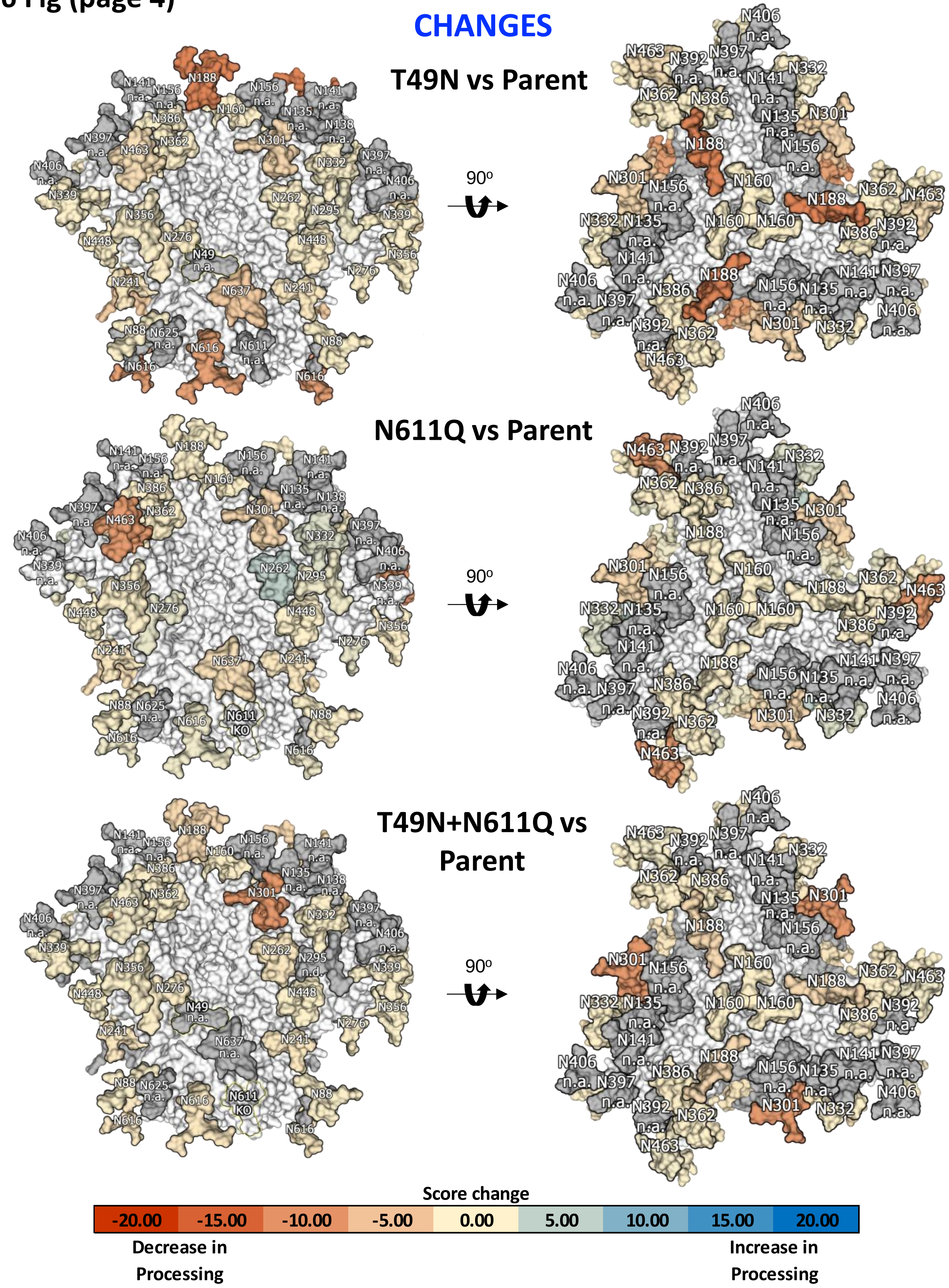

S6 Fig (page 5)

SCORE CHANGES

T49N+N611Q vs T49N

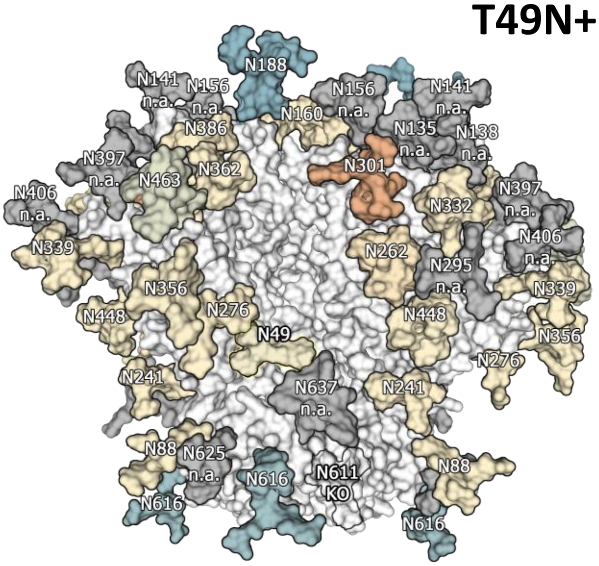

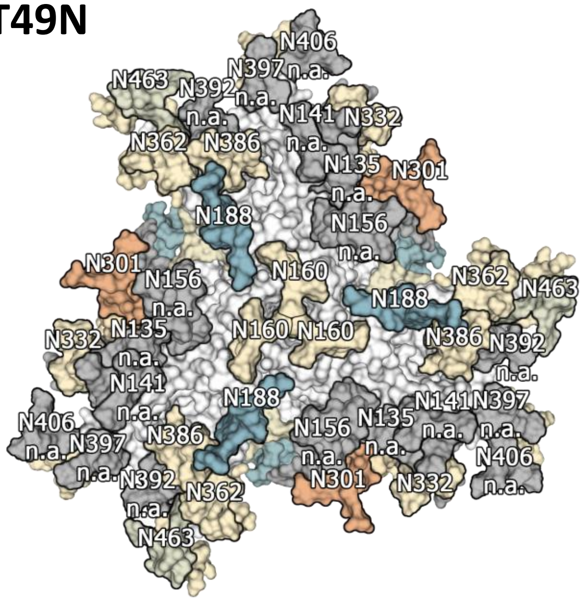

T49N+N611Q vs N611Q

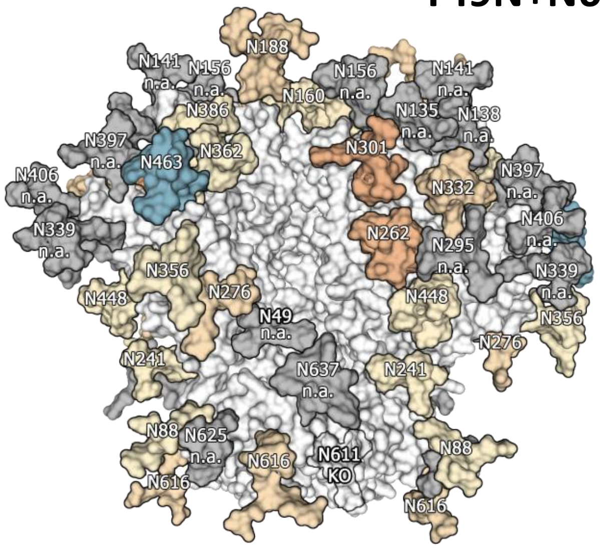

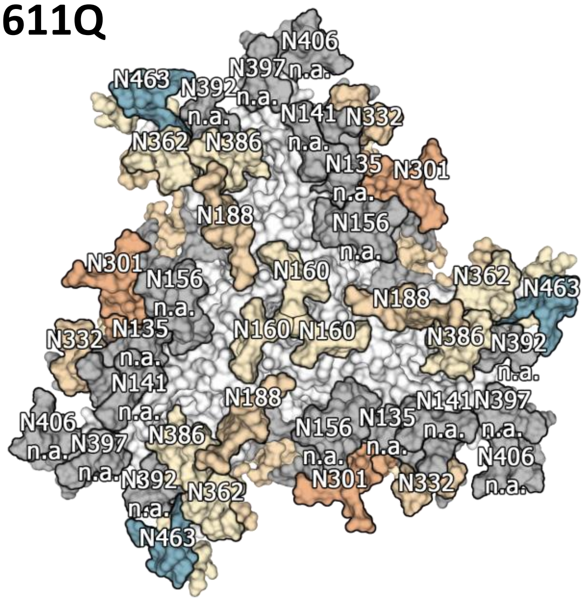

Score change

-20.00	-15.00	-10.00	-5.00	0.00	5.00	10.00	15.00	20.00

Decrease in Processing

Increase in Processing

S6 Fig (page 6)

SCORE CHANGES

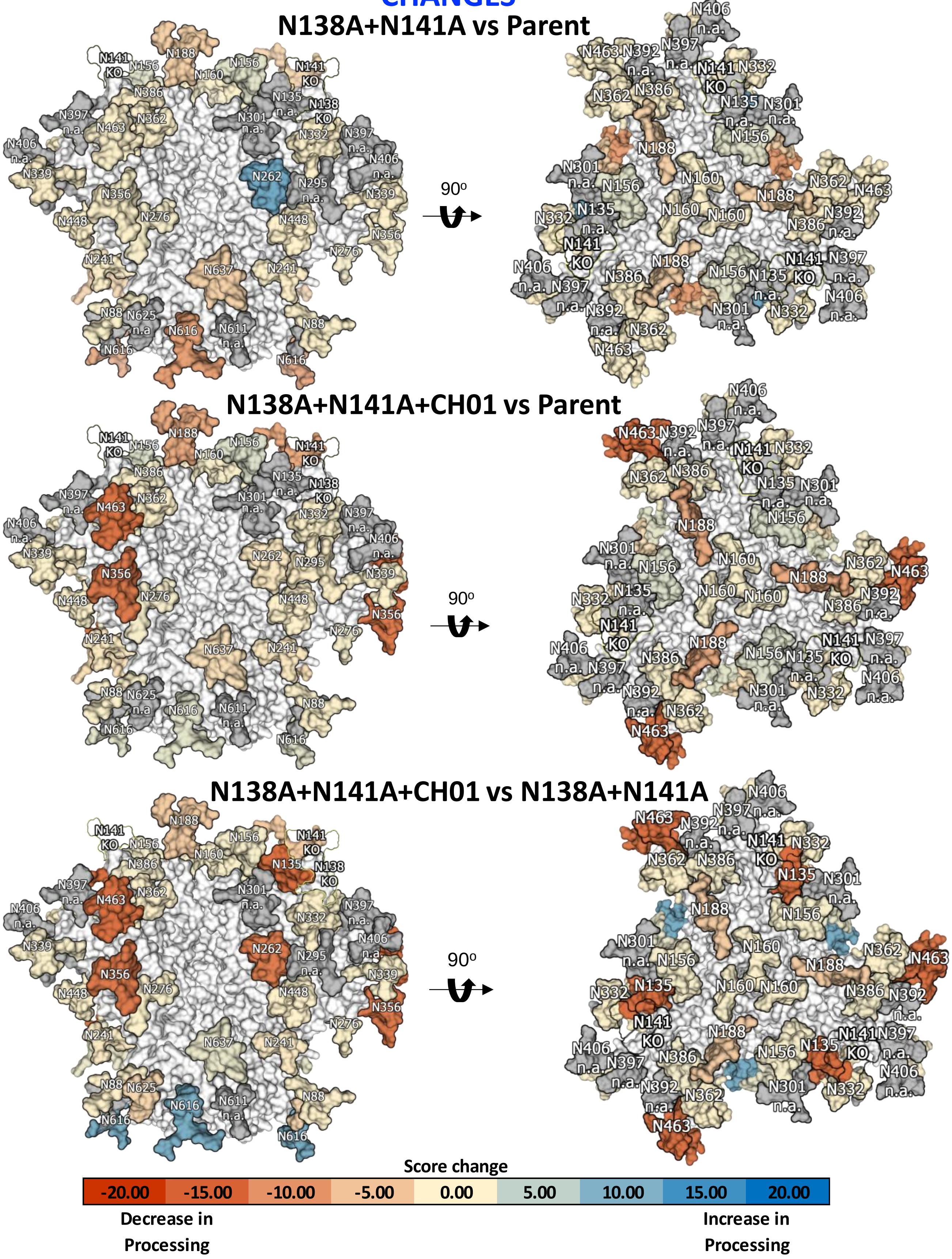

Supplement: S6 Fig — Related to Figs 5B and S5 and S1 Data and analysis. Trimer models depict glycan score differences between sample pairs. Increases in glycan maturation are depicted in progressively bolder hues of blue, while decreases in maturation are indicated in progressively bolder hues of red. Unchanged glycan scores are shown in yellow. Mutant locations are indicated as in S5 Fig. (PDF) [file ppat.1009807.s006.pdf]
