# Supplementary material for: Engineering well-expressed, V2-immunofocusing HIV-1 envelope glycoprotein membrane trimers for use in heterologous prime-boost vaccine regimens
Source: PLoS Pathog. 2021 Oct 22;17(10):e1009807. doi: 10.1371/journal.ppat.1009807 (PMC8565784; doi:10.1371/journal.ppat.1009807)
Supplement: S1 Text — (DOCX) [file ppat.1009807.s014.docx]

**S1 Text: Verifying V3 MAb epitopes to monitor misfolding in a post-sCD4 neutralization assay**

We checked trimers for overt sensitivity to V3 MAbs. First, we needed to check if V3 MAbs cross-react with each trimer. PVs were first mixed with soluble CD4 (sCD4), then we checked if V3 MAbs 14e and 39F inhibited the ability of PV-sCD4 complexes to infect CCR5-expressing cells by [1]. In this format, V3 mAbs neutralized 11 strains. V3 sensitivity of the other 5 strains of our panel could not be confirmed, as their infectivities were too low (CAP45, CM244, KER2018, BB201, X2278).

**A**

**B**

**Fig. V3 epitopes are exposed in the post-CD4 neutralization assay.** A) PV sensitivities to 39F and 14e were assessed in a post-sCD4 neutralization assay. B) 14e MAb titration against c1080 PVs in the standard and post-sCD4 formats.

1. Crooks ET, Moore PL, Richman D, Robinson J, Crooks JA, Franti M, et al. Characterizing anti-HIV monoclonal antibodies and immune sera by defining the mechanism of neutralization. Hum Antibodies. 2005;14(3-4):101-13.
