# Supplementary material for: Engineering well-expressed, V2-immunofocusing HIV-1 envelope glycoprotein membrane trimers for use in heterologous prime-boost vaccine regimens
Source: PLoS Pathog. 2021 Oct 22;17(10):e1009807. doi: 10.1371/journal.ppat.1009807 (PMC8565784; doi:10.1371/journal.ppat.1009807)
Supplement: S2 Text — (DOCX) [file ppat.1009807.s015.docx]

**S2 Text. Effects of JR-FL mutations on Env mobility and endo H sensitivity in SDS-PAGE.**

To rationalize the behavior of JR-FL mutants in Fig 4, we evaluated them in SDS-PAGE-Western blot. Notably, N49 mediated a slight gp41 mass decrease, along with the expected slight gp120 mass increase (Fig A, lanes 1 and 2). The gp41 mass decrease of the T49N mutant was less than that of the N611Q mutant that knocks out a gp41 glycan (Fig A, lanes 1, 3, 5 and 6). As expected, the D197N mutant increased gp120 mass, but unlike T49N, did not impact gp41 mass (Fig A, lanes 2 and 4). T49N+D197N led to a bigger gp120 mass increase than either of the single mutants, as glycans were added at both sites (Fig A, lanes 2, 4-6). Conversely, T49N+N611Q did not reduce gp41 mass further than N611Q alone (Fig A, lanes 5 and 6). Thus, the T49N-mediated reduction of gp41 mass is eliminated when combined with N611Q. V1 glycan knockout mutants revealed decreases in gp120 mass that were consistent with removing one or all 3 glycans (Fig A, lanes 7-10). However, gp41 mass did not change. Expression of these V1 mutants was somewhat weaker than the parent, as judged by the reduced gp41 staining (Figs A, lanes 7-10 and 4A).

We next tested the impact of the N49 glycan knock in on gp41 sensitivity to endoglycosidase H (endo H). As reported previously [1], parent JR-FL gp41 was endo H-resistant, consistent with complex glycans. However, T49N mutant exhibited a ladder of endo H-sensitive gp41 species, consistent with reduced glycan maturation (Fig B, lanes 2 and 4). While the N49 glycan is close to gp41 glycan N637 (Fig 5A), it is not close to N611 that clashes with VRC34. In one scenario, reduced N637 glycan maturation might allow greater N611 glycan flexibility, improving VRC34 binding.

**B**

**Endo H sensitivity**

**A**

**JR-FL mutants**


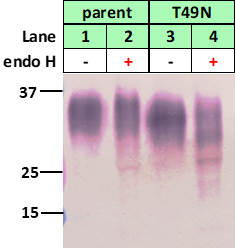

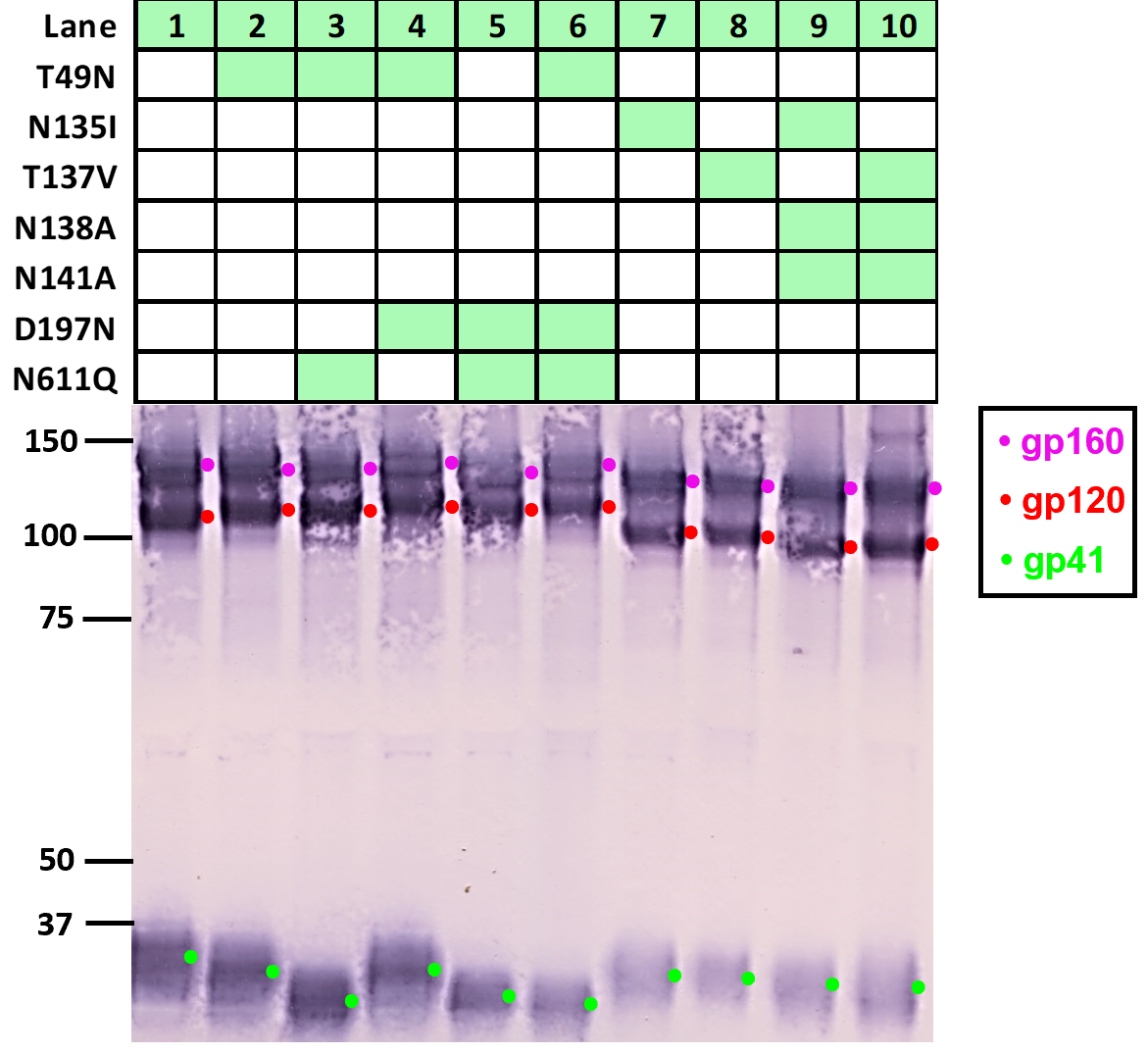


**Fig. Effects of JR-FL mutations on Env mobility and endo H sensitivity.** A) SDS-PAGE-Western blot analysis of various JR-FL VLP mutants reveals changes in gp120 and gp41 mobility. B) The effect of N49 glycan on gp41 endo H-sensitivity of denatured JR-FL VLP Env was probed by SDS-PAGE-Western blot.

1. Crooks ET, Grimley SL, Cully M, Osawa K, Dekkers G, Saunders K, et al. Glycoengineering HIV-1 Env creates 'supercharged' and 'hybrid' glycans to increase neutralizing antibody potency, breadth and saturation. PLoS Pathog. 2018;14(5):e1007024.
