# Supplementary material for: Engineering well-expressed, V2-immunofocusing HIV-1 envelope glycoprotein membrane trimers for use in heterologous prime-boost vaccine regimens
Source: PLoS Pathog. 2021 Oct 22;17(10):e1009807. doi: 10.1371/journal.ppat.1009807 (PMC8565784; doi:10.1371/journal.ppat.1009807)
Supplement: S3 Text — (DOCX) [file ppat.1009807.s016.docx]

**S3 Text: Attempts to improve gp160 processing**

**
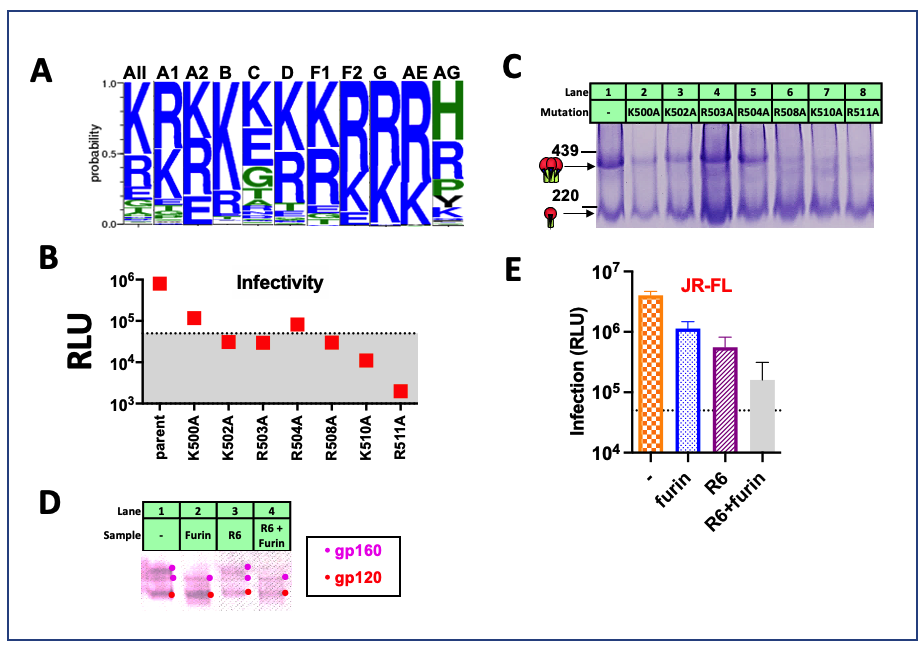
** All of our Env strains contain canonical primary furin cleavage sites REKR (residues 508-511 in S1 Fig). However, a second R/K-rich sequence at residues 500-504 (typically "KAKRR") may contribute to processing. Furin-mediated cleavage occurs in the trans Golgi and is constrained by heavy Env glycosylation. K500 is common, but there are clade-specific polymorphisms (Fig A). E500 is common in clade C (e.g., CNE58 and CAP45) and H500 is common in clade AG (e.g., T250) (Figs 1 and S1). JR-FL cleavage site mutants all showed reduced infectivity. Mutations at the primary site were more critical, especially R511, but those at the secondary site, particularly K500 and R504 had the least effect (Fig B). Expression of all mutants was lower than the parent (Fig C). 508-511 mutant trimers were nearly undetectable. 500 and 502 mutant expression was also lower (Fig C, lanes 2 and 3). Migration of the 502-504 mutant trimers was also modestly reduced, suggesting possible unfolding and/or reduced furin processing (Fig C). Mutants at positions 503-511 caused increased monomer, that consists of uncleaved gp160 precursor. Given the critical role of the secondary cleavage site in trimer folding, we infer that it may be useful to modify strains for K/R500 where necessary. Given that gp140 processing at the primary cleavage site is improved by RRRRRR (R6) mutation and by furin co-transfection [1], we tested if the same is true for membrane trimers. Furin improved gp160 processing, but the R6 mutant reduced expression (Fig D, lane 3). Infectivity of furin and R6-modified PVs were all lower than the parent (Fig E). Overall, both approaches led to reduced membrane trimer expression and infectivity.

**Fig. Impact of cleavage site mutations and furin co-transfection on gp160 processing.** A) Logo plot showing residue frequencies at position 500 of the secondary cleavage site in HIV-1 clades (see also S1 Fig). B) Infectivity of JR-FL SOS mutants carrying the cleavage site mutations indicated. C) BN-PAGE-Western blot of the same mutants in part B. D) SDS-PAGE Western blot showing the effect of furin and the R6 mutation. E) Effect of furin and R6 mutation on JR-FL infectivity.

1. Binley JM, Sanders RW, Master A, Cayanan CS, Wiley CL, Schiffner L, et al. Enhancing the proteolytic maturation of human immunodeficiency virus type 1 envelope glycoproteins. J Virol. 2002;76(6):2606-16.
