# Supplementary material for: Engineering well-expressed, V2-immunofocusing HIV-1 envelope glycoprotein membrane trimers for use in heterologous prime-boost vaccine regimens
Source: PLoS Pathog. 2021 Oct 22;17(10):e1009807. doi: 10.1371/journal.ppat.1009807 (PMC8565784; doi:10.1371/journal.ppat.1009807)
Supplement: S4 Text — (DOCX) [file ppat.1009807.s017.docx]

**S4 Text: Engineering strains c1080 and 6101**

**c1080**

c1080 is a well-expressed group 1 clade AE strain (Figs 3B and 1). Gp160 truncation (gp160ΔCT WT) led to a loss of PG9 sensitivity, which was partly restored by the SOS mutant, albeit with modest V3-sensitivity (Fig A). D49N markedly improved expression (Fig B) but led to overt 14e-sensitivity. This may be because the SOS parent is already partially V3-sensitive, like T250 SOS parent (S10 Fig). In contrast, D49N had little effect on PG9 or CH01 sensitivities (Fig A), again showing that increases in V3 sensitivity can occur without the loss of V2 sensitivities.

The unusual H375 residue of c1080 and other AE strains could impact trimer compactness and/or CD4 sensitivity [1]. H375S reversion did not improve CH01 sensitivity (Fig A). Moreover, similar to D49N, it was overtly 14e-sensitive (Fig A). The D49N and H375S mutants are more V3-sensitive than they are to V2 MAbs. Therefore, any further mutants should not be combined with either D49N or H375S. Since the parent virus is partially V3-sensitive, a strategy akin to T250 may be effective (i.e., avoiding D167N, removing clashing glycans). However, we did not pursue c1080 further, as our T250 mutant suffices as a late shot - and is also CH01 UCA-sensitive (Fig 8B, lane 5).


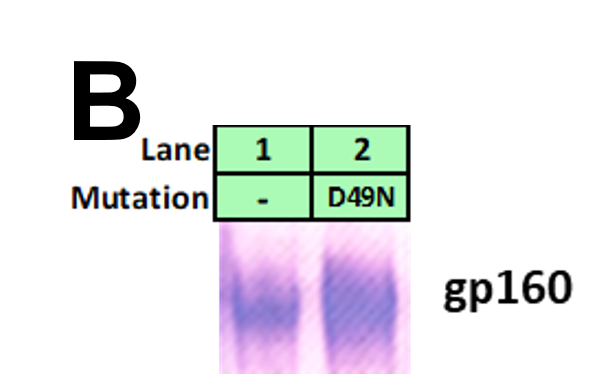

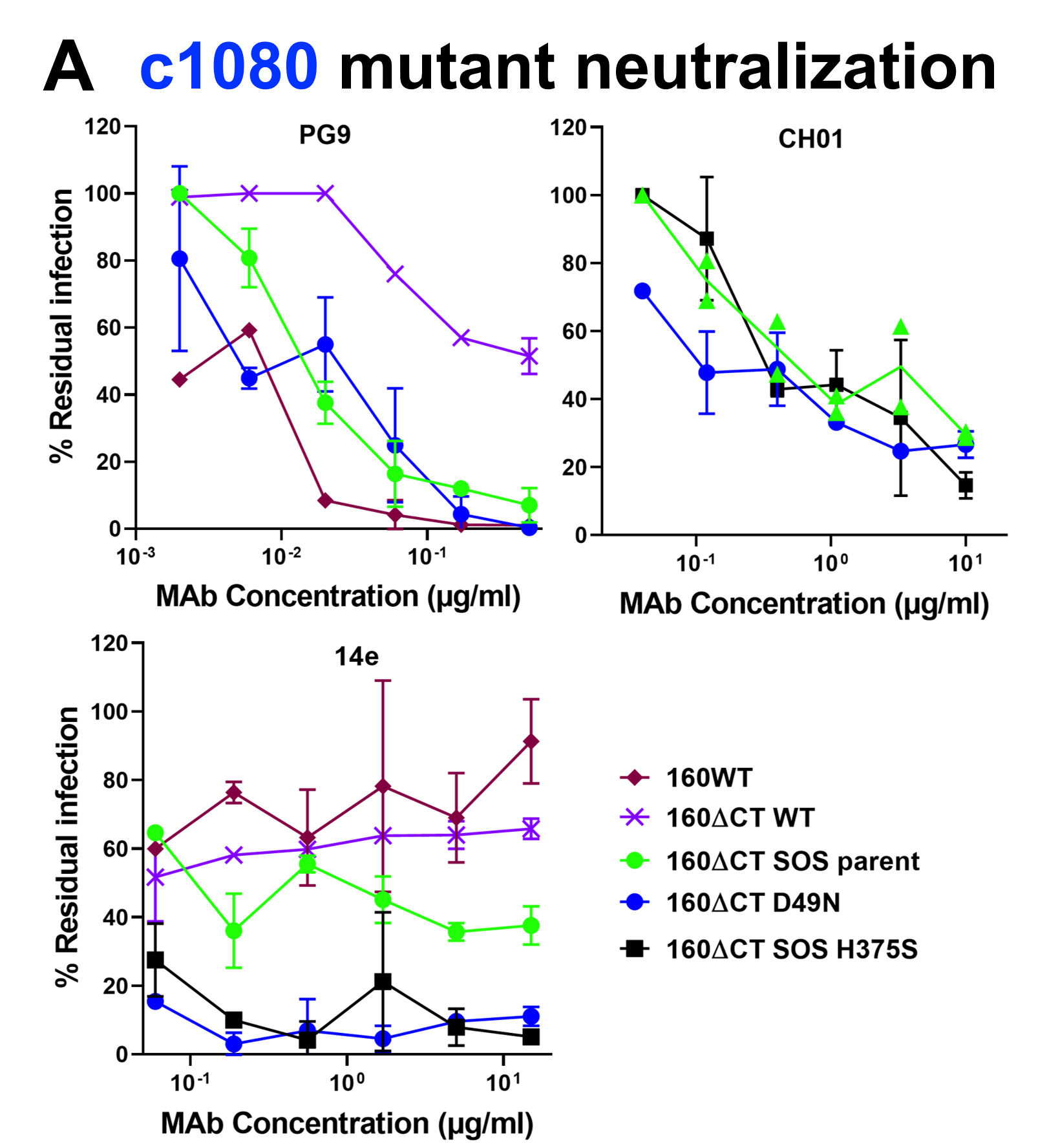


**Fig. Effects of mutations on c1080 pseudovirus MAb sensitivities and expression.** A) We compared various versions of the c1080 strain, with or without gp41 truncation, SOS mutation, D49N glycan knock in and H375S mutation for sensitivity to PG9, CH01 and 14e. B) Comparison of SOS gp160ΔCT Env expression with or without D49N mutations by SDS-PAGE-Western blot.

**6101**

Of the group 2 strains, 6101 is the weakest expressing (Fig 3B-D) and lacks V2-sensitivity (Fig 1). Nevertheless, we attempted repairs by using mutant combinations, including T49N, N130H, D160N, DK166-167RD, T171K, D177Y, GG269-269E, and 355G to optimize V2 sensitivity and resolve insertions and deletions. Although some mutants were infectious with RLUs >500,000, neutralization data was difficult to interpret so we did not pursue this strain further.

1. Li H, Wang S, Kong R, Ding W, Lee FH, Parker Z, et al. Envelope residue 375 substitutions in simian-human immunodeficiency viruses enhance CD4 binding and replication in rhesus macaques. Proc Natl Acad Sci U S A. 2016;113(24):E3413-22.
