# Supplementary material for: Engineering well-expressed, V2-immunofocusing HIV-1 envelope glycoprotein membrane trimers for use in heterologous prime-boost vaccine regimens
Source: PLoS Pathog. 2021 Oct 22;17(10):e1009807. doi: 10.1371/journal.ppat.1009807 (PMC8565784; doi:10.1371/journal.ppat.1009807)
Supplement: S5 Text — (DOCX) [file ppat.1009807.s018.docx]

**S5 Text: Other engineering approaches.**


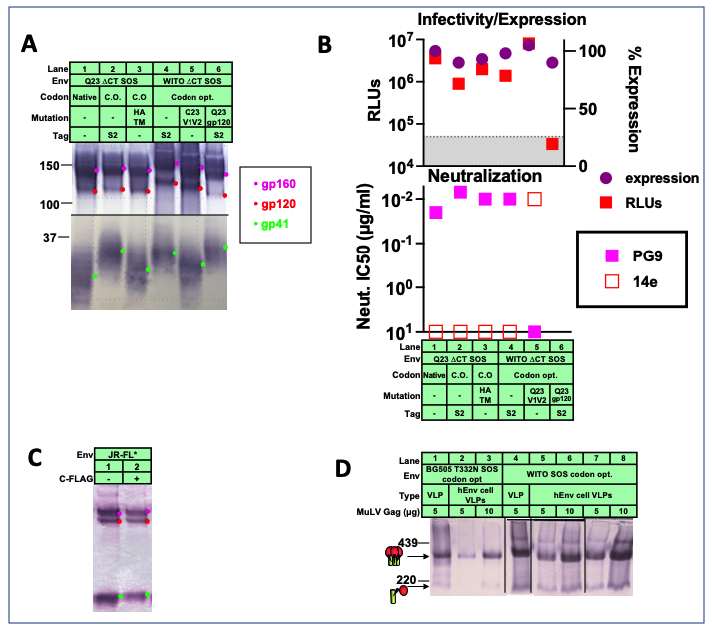
We evaluated i) codon optimization, ii) modified leader peptides, iii) chimeras in which gp120, V1V2 loops or transmembrane domains were swapped with well-expressed alternatives, iv) Env-transduced cell lines and v) C-terminal truncations and tags. In many cases, to accelerate screening, we combined more than one approach, reasoning that if either or all approaches succeeded, we would see an effect. In the examples below, we compared expression, infectivity and V3 sensitivity of several Q23 and WITO clones (see Fig A and B). We found that Q23 codon optimization, adding an S2 tag and CD5 leader resulted in lower gp160 and gp120 expression, and an expected increase in gp41 band size due to the tag (Fig A, compare lanes 1 and 2). Replacing the natural Q23 TM (AA 684 to 705) with that of influenza HA with a truncated cytoplasmic tail (ACQKG) did not impact expression (Fig A, compare lanes 1 and 3), contrasting with previous findings [1], where uncleaved Envs were expressed in insect cells. This suggests that the expression benefit of TM swaps is context-dependent. A codon-optimized WITO chimera with a Q23 V1V2 (AA 130 to 196) expressed well, perhaps in part due to a lack of S2 tag. In contrast, WITO with a Q23 gp120 graft (AA 34 to 497) [2-4] expressed poorly (Fig A, lanes 4-6).

We next checked the infectivity and MAb sensitivity of these clones. All but the gp120 chimera were functional. The V1V2 chimera was overtly V3-sensitive and lost PG9 sensitivity. Conversely, the other mutants retained PG9 sensitivity and 14e-resistance (Fig B).

Above, it appeared that mutants with an S2 tag (Fig A, lanes 2, 4, 6) expressed somewhat less effectively than mutants lacking a tag (Fig A, lanes 1, 3, 5). However, the other differences between these clones render it difficult to definitively conclude that the tag reduces expression. Therefore, we compared JR-FL clones with or without a C-terminal FLAG tag (DYKDDDDK). The tag slightly reduced expression (Fig C, compare lanes 1 and 2), supporting the idea that tags reduce Env expression and that the loss in expression is sequence-independent. In further studies, we found that truncating JR-FL Env further upstream than residue 708 did not improve Env expression.

**Fig. Other engineering approaches to improve membrane Env expression.** Combination mutants were made to investigate the effects of other strategies on trimer expression. Example mutants of Q23 and WITO were analyzed A) by SDS-PAGE Western blot, and B) for infectivity in the pQC-Fluc assay and V3-sensitivity. C) The effect of a C-terminal FLAG tag alone on JR-FL SOS E168K+N189A expression was checked by SDS-PAGE-Western blot. D) Comparison of trimer expression of VLPs made by our conventional Gag+Env transfection, as compared to transfecting Env-expressing hEnv cells made by transduction and 2G12 sorting. hEnv cells were transfected with different dose of MuLV Gag, as indicated.

Finally, we evaluated lentiviral transduction to make Env-expressing cell lines, as described previously [5]. In the examples in Fig D, we made cell lines by cloning and growing single “hEnv” cells, selected by flow cytometry using mAb 2G12. These cells were transfected with Gag only to make hEnv VLPs that were then compared to regular VLPs made by transfecting Gag+Env plasmids. Env expression was generally equivalent in hEnv VLPs made by transfecting hEnv cell lines with a high dose of Gag (Fig D, lanes 3, 6 and 8), compared to their regular VLP counterparts (Fig D, lanes 1 and 4). The lack of clearly improved expression, the labor of sorting and making cell lines and, moreover, the inability to make Env sequence adjustments, led us to abandon this approach. Overall, these additional approaches did not clearly improve membrane trimer expression and, in some cases, did the opposite.

1. Wang BZ, Liu W, Kang SM, Alam M, Huang C, Ye L, et al. Incorporation of high levels of chimeric human immunodeficiency virus envelope glycoproteins into virus-like particles. J Virol. 2007;81(20):10869-78.

2. Joyce MG, Georgiev IS, Yang Y, Druz A, Geng H, Chuang GY, et al. Soluble Prefusion Closed DS-SOSIP.664-Env Trimers of Diverse HIV-1 Strains. Cell Rep. 2017;21(10):2992-3002.

3. Gorman J, Soto C, Yang MM, Davenport TM, Guttman M, Bailer RT, et al. Structures of HIV-1 Env V1V2 with broadly neutralizing antibodies reveal commonalities that enable vaccine design. Nat Struct Mol Biol. 2016;23(1):81-90.

4. Andrabi R, Voss JE, Liang CH, Briney B, McCoy LE, Wu CY, et al. Identification of Common Features in Prototype Broadly Neutralizing Antibodies to HIV Envelope V2 Apex to Facilitate Vaccine Design. Immunity. 2015;43(5):959-73.

5. Stano A, Leaman DP, Kim AS, Zhang L, Autin L, Ingale J, et al. Dense Array of Spikes on HIV-1 Virion Particles. J Virol. 2017;91(14):e00415.
