# Supplementary material for: Engineering well-expressed, V2-immunofocusing HIV-1 envelope glycoprotein membrane trimers for use in heterologous prime-boost vaccine regimens
Source: PLoS Pathog. 2021 Oct 22;17(10):e1009807. doi: 10.1371/journal.ppat.1009807 (PMC8565784; doi:10.1371/journal.ppat.1009807)
Supplement: S6 Text — (DOCX) [file ppat.1009807.s019.docx]

**S6 Text SUPPLEMENTAL INFORMATION - Vesicle Flow Cytometry detailed methods**

**Vesicle Flow Cytometry.** Single vesicle flow cytometry (Fig A) was performed using a commercial assay kit (vFC EV Analysis Kit, Cellarcus Biosciences; Table A) and flow cytometer (CellStream, Luminex). Sample staining was performed in 96-well v-bottom plates as directed in the vFC^T^ Protocol. A fresh 10× vFRed stain solution was prepared from the 100× stock in vesicle Staining Buffer. Staining reactions consisted of 5 µl diluted sample optimal dilution (determined in preliminary measurements), 5 µl vFRed 10x stain solution, and 5 µL 10× FL-marker (FL-antibody) added to a total volume of 50 µL in vesicle Staining Buffer. Samples were incubated for 1 h at ambient temperature, followed by a post-stain dilution (200-fold) for analysis on the CellStream.

| **Table A. Reagents for EV flow cytometric assays** | | |
| --- | --- | --- |
| Reagent [clone] | Source | Catalog number |
| vFC EV Analysis Assay kit | Cellarcus Biosciences | CBS4 |
| Anti-HIV-1 gp120 [PGT121] | NIH AIDS Reagents Program | ARP-12343 |

**Fig A. Schematic of VFC workflow.** Vesicle flow cytometry (vFC) is a homogeneous assay in which a cell-free sample, prepared by centrifugation, is stained with a fluorogenic membrane stain and one or more additional fluorescence probes then analyzed by flow cytometry with detection triggered by membrane fluorescence. The size distribution of a synthetic vesicle standard (Lipo100), determined by NTA, is used to calibrate membrane fluorescence in terms of vesicle surface area, while fluorescence intensity and antibody capture standards are used to calibrate fluorescence intensity and MAb binding in units of MESF (mean equivalent soluble fluorochromes) or ABV (Abs bound per vesicle).

| **Table B. CellStream detector configuration** | | | |
| --- | --- | --- | --- |
| Detector | Laser-Filter | Stain | Power |
| A1 | 775 - 773/56 |  | 0 |
| A2 | 405 - 456/51 |  | 100 |
| A3 | 405 - 528/46 |  | 100 |
| A4 | 405 - 583/24 |  | 100 |
| A5 | 405 - 611/31 |  | 100 |
| A6 | 405 - 702/87 |  | 100 |
| B1 | 642 - 773/56 |  | 100 |
| B3 | 642 - 528/46 |  | 100 |
| B4 | 642 - 583/24 |  | 100 |
| B5 | 642 - 611/31 |  | 100 |
| B6 | 642 - 702/87 | Alexa647 | 100 |
| C1 | 488 - 773/56 |  | 100 |
| C2 | no laser - 456/51 |  |  |
| C3 | 488 - 528/46 | GFP | 100 |
| C4 | 488 - 583/24 |  | 100 |
| C5 | 488 - 611/31 |  | 100 |
| C6 | 488 - 702/87 | vFRed | 100 |
| D1 | 561 - 773/56 |  | 100 |
| D2 | FSC - 456/51 |  | 0 |
| D3 | no laser - 528/46 |  |  |
| D4 | 561 - 583/24 | PE | 100 |
| D5 | 561 - 611/31 |  | 100 |
| D6 | 561 - 702/87 |  | 100 |

**Flow cytometer configuration and operation.** The CellStream flow cytometer was used in its standard configuration (Table B) with the FSC and SSC lasers turned off and its Small Particle mode activated. The instrument fluorescence response was calibrated using hard-dyed calibration beads (nanoRainbow, Cellarcus) that were cross-calibrated against MESF (Mean Equivalent Soluble Fluorochromes; PE QuantiBrite beads, BD Biosciences #340495; Quantum FITC MESF beads, Bangs Labs #555) and antibody capture beads (units of Antibodies Bound per Vesicle, ABV; vCal™ AbCap, Cellarcus #CBS7-MS). Vesicle surface area was estimated using a vFRed™-stained vesicle standard whose size distribution was characterized using NTA (Lipo100™, Cellarcus). For vFC™, sample was introduced at the Slow sample flow rate (3.66 µL/min, confirmed using vCal™ nanoRainbow beads) and each sample well was analyzed for 120 seconds.

**Vesicle gating and data analysis.** Data were analyzed using a standardized data analysis layout created in FCS Express version 7 (De Novo Software). Data were subjected to a Time gate (Fig BI), to remove data associated with any fluidic anomalies (e.g., clogs or surges), vFRed Object Area (Fig BII) to remove events recorded objects imaged over more than four pixels, which would be inconsistent with single EVs). These events were further gated (Vesicle gate, Fig BIII) to include events with membrane fluorescence in the blue-excited, red-emission channels of vesicles and to exclude low intensity low intensity background events. Presented are data from buffer (+vFRed, left), Lipo100 Vesicle Size Standard (center), and a VLP preparation (right). The vFRed fluorescence of Vesicle-gated events (Fig BIV) was used to estimate vesicle size and events counts in the Vesicle gate in 3.66 ul were used to estimate the EV concentrations in the original sample, after accounting for the pre-stain and post-stain dilutions.

**Fig B. VFC gating.** I) Flow cytometry data is gated on time (to exclude data marred by any fluidic disturbances), II), vFRed object area, (to exclude objects larger than four pixels, which would be inconsistent with single EVs), III) and by a gate (Vesicles), IV) on the characteristic vFRed fluorescence in the blue-excited red emission channels. Gating is illustrated for Buffer (left) Lipo100 vesicle standard (center), and VLPs (right) stained according to the schematic in Fig A.

**Vesicle size calibration.** Vesicle size was estimated using the fluorescence from a vesicle size standard (Lipo100™, Cellarcus) whose size distribution was measured by nanoparticle tracking analysis (NTA, NanoSight, Malvern) and resistive pulse spectroscopy (RPS, nCS-1, Spectradyne). The Lipo100 standard was stained and measured under the same conditions as samples, and the relationship between population surface area (estimated by NTA, Fig CI) and membrane fluorescence (measured by FC, Fig CII) was used to calculate the surface area per fluorescence intensity unit (Fig CIII). This factor was used to calibrate the membrane fluorescence axis in units of surface area (Fig CIII) and diameter (Fig CIV), assuming spherical particles. The vesicle size detection limit, estimated from the calibrated trigger threshold surface area and diameter detection, is ~75 nm.

**Fig C. Vesicle size estimation.** Vesicle size was estimated from the relationship between fluorescence intensity and vesicle surface area, as determined by the staining and analysis of a well-characterized synthetic vesicle size standard (Lipo100, Cellarcus Biosciences). The Lipo100 diameter distribution measured by NTA (I) was used to calculate the surface area distribution which, when compared to the fluorescence intensity distribution after staining with vFRed (II), showed a linear relationship (III) that was used to convert the arbitrary vFRed fluorescence intensity into units of estimated equivalent surface area and diameter (IV).

**Controls for single vesicle analysis.** The specificity of single vesicle analysis was evaluated via several control measurements. Buffer plus vFRed showed low levels of background events (~2000 in ~120 seconds of gated data; Fig DI), while detergent treatment (0.1% Empigen added before the post-stain dilution) resulted in lysis of >90% of gated events, indicating that most detected events were detergent-labile, as expected for EVs. Serial dilution of sample (Fig DII) showed the expected proportional decrease in the number of detected events (Fig DIII) with minimal change in the brightness of those events (Fig DIV), consistent with the measurement of single EVs.

**Fig D. VFC detergent and dilution controls.** The vesicular nature of the measured events was demonstrated by treatment with 0.1% triton X100 (I), which eliminated them. The working sample dilution range was determined by serial dilution (II-IV), which also demonstrated no discernable coincidence/swarm.

**Vesicle immunofluorescence calibration, controls, and reporting.**  Immunofluorescence negative controls included the Lipo100 vesicle standard, which bears no antigen (Fig EI), WITO VLPs (Fig EII) and bald VLPs (Fig EIII). EV immunofluorescence was calibrated in ABV (antibodies bound per vesicle) units for the anti-gp120 [PGT121] AlexaFluor647, using calibrated antibody capture beads (Fig EIV) and cross-calibrated nanoRainbow beads (Fig EV), as described above (Table B). Because the PGT121 clone is human, and does not bind to mouse immunoglobulin capture beads, we used beads stained with a similarly-labeled AlexaFluor647 mouse IgG. The median fluorescence intensity of the entire positive EV population, the number of positive EVs with fluorescent intensity higher than the upper threshold of an unstained sample (<0.5% “positive”), and the MFI of positive EVs were calculated using standardized data analysis layouts created using FCS Express version 7.

**Fig E. Immunofluorescence staining specificity.** The specificity of the labeled anti-Env antibody was assessed using (I) Lipo100™, which bears no antigen, (II) WITO VLPs, and (III) Bald VLPs. Calibration was performed using IV) antibody capture beads and V) nanoRainbow beads.
